# Structures of protein folding intermediates on the ribosome

**DOI:** 10.1038/s41594-026-01814-7

**Published:** 2026-06-16

**Authors:** Sammy H. S. Chan, Julian O. Streit, Tomasz Włodarski, Alkistis N. Mitropoulou, Anaïs M. E. Cassaignau, Ivana V. Bukvin, Christopher A. Waudby, Lisa D. Cabrita, John Christodoulou

**Affiliations:** 1 Institute of Structural and Molecular Biology, Department of Structural and Molecular Biology, University College London, London, UK; 2 Institute of Biochemistry and Biophysics, Polish Academy of Sciences, Warsaw, Poland; 3 Department of Pharmaceutical and Biological Chemistry, School of Pharmacy, University College London, London, UK; 4 School of Natural Sciences, Birkbeck College, London, UK

**Keywords:** Solution-state NMR, Ribosome

## Abstract

The ribosome biases the conformations sampled by nascent polypeptide chains along folding pathways toward biologically active states. A hallmark of the cotranslational folding (coTF) of many proteins constitutes highly stable folding intermediates that are absent or only transiently populated off the ribosome, yet persist during translation well beyond complete emergence of the domain from the ribosome exit tunnel. Despite the importance of intermediates for folding fidelity, their structures have remained elusive and cannot be predicted by machine learning methods. Here we obtained structures of two folding intermediates of an immunoglobulin-like domain on the ribosome by developing comprehensive ^19^F nuclear magnetic resonance analyses using chemical shifts by rational design, paramagnetic relaxation enhancement and protein engineering, integrated with extensive molecular dynamics simulations. The resulting intermediate structures reveal native-like folded cores distinguished by nonnative termini, permitting distinct binding to a molecular chaperone and suggesting parallel folding pathways. The structures of these intermediates are conserved within the protein domain family, in contrast to their in vitro refolding mechanisms. Our detailed structural ensembles of partially folded nascent proteins on the ribosome highlight the diversity of conformations sampled during coTF, providing the ribosome with a passive means to promote efficient protein folding and maintain cellular proteostasis.

## Abstract (web summary)

Atomistic structural ensembles of protein folding intermediates on the ribosome are resolved by comprehensive ^19^F nuclear magnetic resonance analyses integrated with molecular dynamics simulations, providing insights into cotranslational folding pathways.

## Main

During biosynthesis, nascent proteins emerge from the ribosome and can begin to acquire their native biologically active structures^1^. The ribosome itself is thought to regulate cotranslational folding (coTF) by modulating the nascent polypeptide chain’s thermodynamic stability^2–8^ and (un)folding rates^2,6,9^, through vectorial emergence^10,11^, steric restriction of the available conformational space^12^, interactions with its highly charged surface^5,13–15^ and with specific ribosomal proteins^16–18^ and the recruitment of molecular chaperones^19–22^. In particular, the physical chemistry of the ribosome results in the structural expansion and entropic destabilization of unfolded nascent proteins^3^ and enthalpic destablization of negatively charged folded states by long-range repulsive electrostatic forces^23^. Consequently, for negatively charged proteins (~50% proteome^24^), thermodynamically stable folding intermediates are the predominant conformer outside the ribosome exit tunnel^2,3,23^, where most tertiary folding occurs. Accordingly, CoTF contrasts with studies measuring the refolding of full-length denatured protein, where intermediates are only transiently populated^3^ and folding outcomes differ with generally higher propensities to misfold or aggregate; coTF is, therefore, a cellular mechanism to minimize kinetic misfolding traps^25–28^. Detailed descriptions of coTF pathways are absent, although structures of folding intermediates would provide mechanistic insights into how the ribosome biases nascent chain conformations away from hazardous states^27,29,30^ and toward conformations that are biologically active^3^ or accessible for cotranslational assembly^31,32^ or chaperoning processes^19^.

CoTF intermediates have generally been reported to be structurally compact compared to their unfolded states^33–35^, with some observations of native-like structure formation^2,3^ within ensemble conformations^15,36,37^. However, unlike most mature proteins, nascent polypeptides inherently interconvert between multiple conformational states with similar thermodynamic stabilities^2,38^, presenting a technical challenge to their structural characterization. Solution-state nuclear magnetic resonance (NMR) spectroscopy of ribosome–nascent chain complexes (RNCs) has enabled residue-level measurements of different nascent protein conformations by using specific isotopic labeling schemes^3,5,13,16,39–41^, providing experimental data to guide the determination of accurate structures of unfolded^3,13^ and folded^23^ RNCs from all-atom molecular dynamics (MD) simulations. The direct observation and resolution of all conformations, including multiple partially folded coTF intermediates, has uniquely been enabled by ^19^F NMR^2,3,16^, which has the potential to extract structural information^42,43^.

Here, we developed an array of generalizable ^19^F NMR analyses for structural biology and obtained direct structural contacts and intramolecular distances within two distinct coTF intermediate conformations populated by an immunoglobulin-like domain on the ribosome. These ^19^F NMR experiments unambiguously and quantitatively validate atomistic structural models of the coTF intermediates, obtained using biased folding simulations and extensive unbiased MD simulations, and show their conservation across immunoglobulin folds.

## CoTF folding intermediates identified by ^19^F NMR

**Fig. 1:**
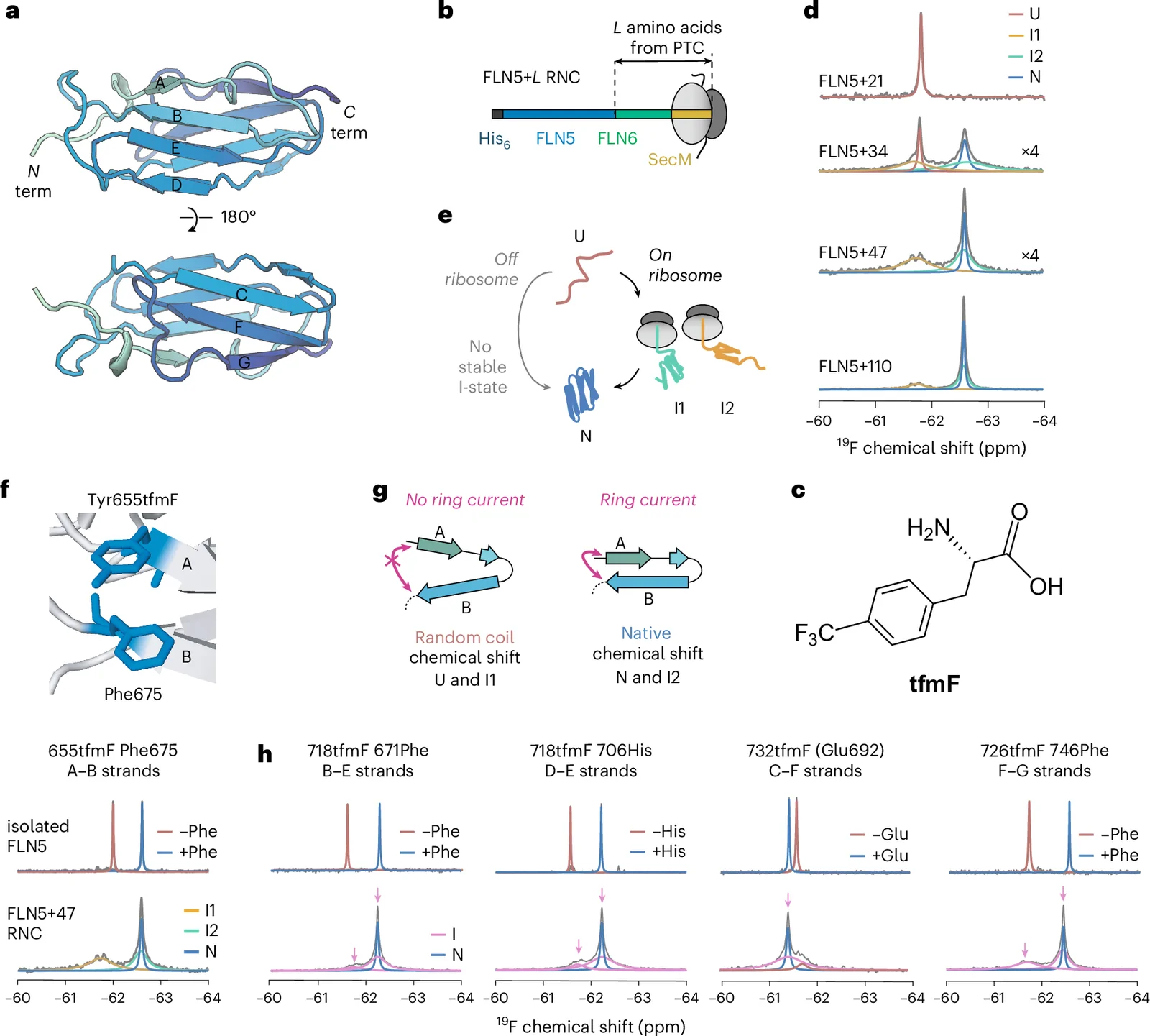
Structural characterization of FLN5 coTF intermediates using ^19^F NMR chemical shifts. **a**, Crystal structure of FLN5 (PDB 1QFH) with β-strands labeled. **b**, Schematic of FLN5 RNCs of varying *L* lengths. PTC, peptidyl transferase center. **c**, Chemical structure of tfmF. **d**, The 1D ^19^F NMR spectra of different lengths of 655tfmF-labeled FLN5 RNCs. Observed spectra are shown in gray. Native (N), unfolded (U) and intermediate state (I1, I2) resonances are indicated and were fitted to Lorentzian lineshapes (Methods). The magnification of spectra by a scaling factor is indicated. **e**, Folding reactions of FLN5 on and off the ribosome. **f**, Top, magnified view of Tyr655 and Phe675 in the FLN5 crystal structure. Bottom, ^19^F NMR spectrum of 655tfmF-labeled isolated wild-type and Phe675Ala mutant FLN5 (top) and FLN5+47 RNC (bottom). **g**, Schematic depicting ring current between two residues on adjacent β-strands and their corresponding ^19^F chemical shifts. **h**, Top, ^19^F NMR spectra of isolated FLN5 with ^19^F-label pair, overlaid with that of tfmF-labeled isolated FLN5 without accompanying aromatic or charged residue (left to right, FLN5 718tfmF 671Phe, FLN5 718tfmF 706His, 732tfmF and 726tfmF 746Phe). Bottom, ^19^F NMR spectra of FLN5+47 RNC with different ^19^F-label pairs. Arrows indicate chemical shifts of intermediate states. Observed spectra are shown in gray. The assignment of resonances is colored accordingly. Spectra were fitted to Lorentzian lineshapes (Methods). All spectra were recorded at 500 MHz and 298 K.

**Extended Data Fig. 1:**
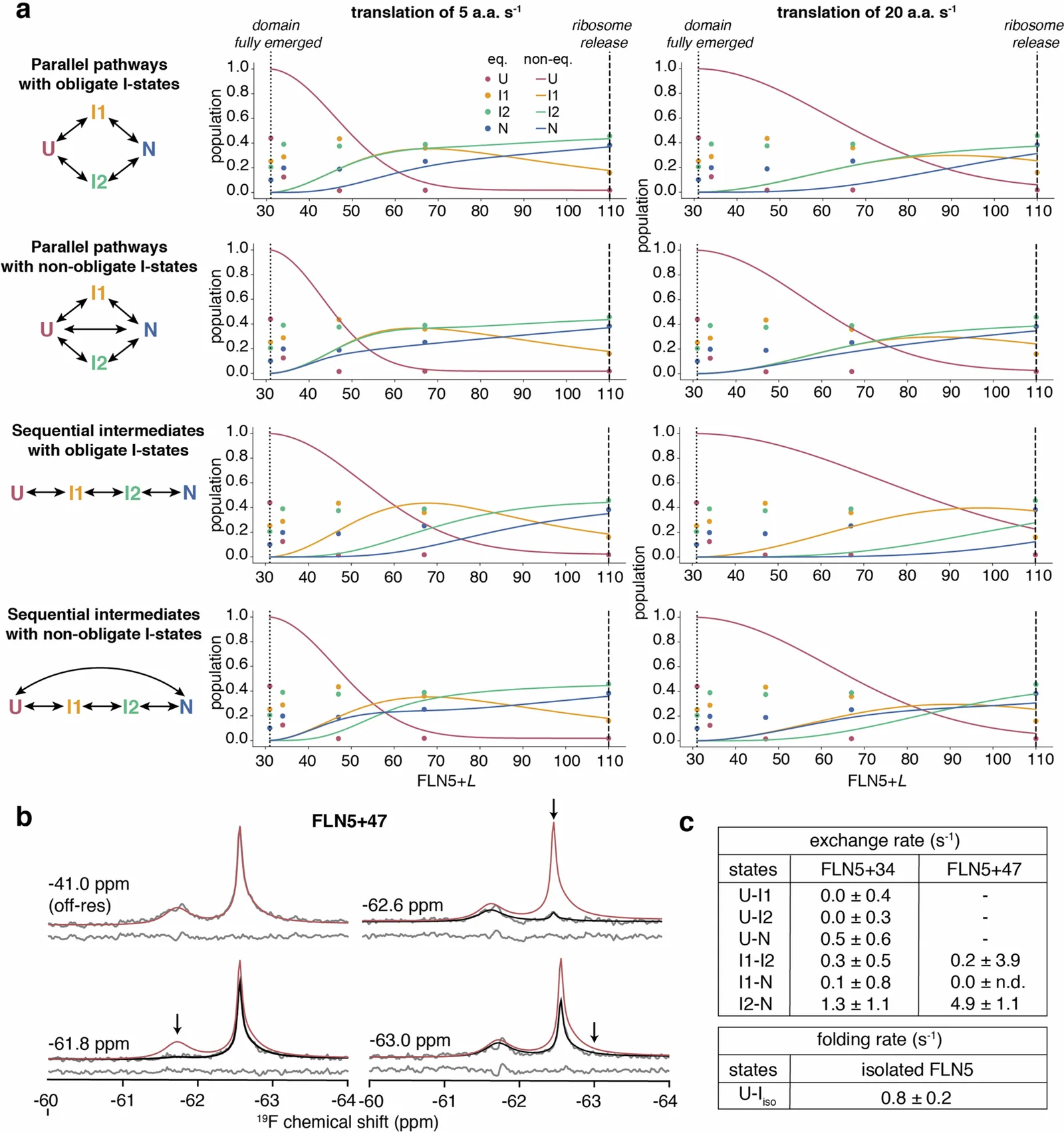
Kinetics of coTF of FLN5. **a**, Kinetic models showing predicted populations of unfolded, intermediate and native states during real-time biosynthesis with a translation rate of 5 and 20 amino acids per second and experimental estimates of folding rates (see Methods). The solid lines represent the predicted values while dots show the experimentally measured equilibrium populations (this work and refs. ^2,3^). The schematics on the left illustrate the interconversions considered in the model, ranging from parallel pathways to sequentially formed intermediates. Simulations start at FLN5+31 (the first linker length at which the entire domain has emerged from the tunnel and begins to fold at equilibrium^39^) and terminate at FLN5+110 at which point the NC is released from the ribosome. **b**, ^19^F CEST measurements of FLN5+47 RNC. Given sensitivity limitations, we selected four frequencies at which to irradiate (of which one was off-resonance from all NMR peaks and shown in red, arrows indicate remaining irradiation frequencies) with the 15-Hz B1 field. Observed spectra (grey) were fitted (black), with residuals shown below, by analysing in the time domain using the Bloch-McConnell equations, as previously described^2^. Spectra were recorded at 298 K, 500 MHz. **c**, Exchange rates between FLN5+34 and FLN5+47 nascent chain states determined by CEST measurements in ref. ^2^ and this work respectively, using an estimated *R*_1_ of 1.1 s^−1^ for all RNC states. Error determined from data fits. The folding rate of isolated FLN5 (truncated variant FLN5∆6) was previously reported^11^. Source data

We previously mapped the folding free energy landscapes of the fifth filamin domain of the *Dictyostelium* *discoideum* gelation factor, FLN5, both off and on the ribosome, using NMR spectroscopy^2,3,5,11,39^. FLN5 is an immunoglobulin-like domain with two sandwiched antiparallel β-sheets (Fig. 1a). In isolation, the full-length protein is a two-state folder under denaturing conditions^2^ and only weakly populates an intermediate when truncated at its C terminus^2,11^. However, when translation-stalled on the ribosome at specific nascent chain lengths (Fig. 1b) and fully emerged from the tunnel^39^, FLN5 predominantly populates two stable coTF intermediates (I1 and I2; Fig. 1d,e) in slow exchange (on the NMR timescale) with the unfolded and natively folded states (Extended Data Fig. 1b,c). The intermediates are detected as broad NMR resonances (because of ribosome surface interactions^2,3^) with site-specific ^19^F-labeling using 4-trifluoromethyl-L-phenylalanine^2^ (tfmF; Fig. 1c) and invisible by other isotopic labeling schemes^11^. Measurement of ^19^F NMR signal integrals showed that the coTF intermediates are up to 5 kcal mol^−1^ more stable than the (truncated) one found off the ribosome (relative to their respective unfolded states) and persist at long nascent chain lengths during translation^2^ (Fig. 1d), despite complete availability of the domain for native folding to occur^39^. Indeed, an analysis of multiple high concentration (20 µM, ~48 mg ml^−1^) purified samples of FLN5+110 RNC, the final biosynthetic snapshot of the entire FLN5-6 protein, reveals high (~62%) populations of the intermediates (Fig. 1d and Supplementary Tables 1 and 2). While these data were obtained under equilibrium conditions, we used a kinetic model to assess whether the I1 and I2 states would also be expected to be formed during real-time translation. By considering the equilibrium populations together with the kinetics of translation and conservative, lower-bound estimates of folding, we anticipate large populations of I1 and I2 during real-time biosynthesis before translation termination (Extended Data Fig. 1).

## Structural contacts revealed by ^19^F NMR chemical shifts

The coTF intermediates adopt a native-like hydrophobic core, evidenced by their complete destabilization upon substitution of the natively buried residue Tyr719 (ref. ^2^); however, further details of their structures are sparse. To begin to extract structural information by ^19^F NMR spectroscopy^44^, we examined ^19^F chemical shifts and their relationship to protein structure, exploiting our recent development of solvent-exposed tfmF labels experiencing ring-current effects^42^, which are distance and angle dependent on nearby aromatic residues. Residue 655 on the A strand directly contacts the aromatic ring of Phe675 on the neighboring B strand; its tfmF chemical shift is consequently shielded from the random coil value by ~0.8 ppm (Fig. 1f)^2^ and correlated to the ring-current effect as shown by the Phe675Ala substitution^42^ (Fig. 1f). The tfmF655 chemical shift is, thus, a direct reporter of the interresidue 655–675 side-chain van der Waals contact^42^ comprising a precise, short-range (4–6 Å) distance and angular preference, as quantifiable by structural modeling^42^. The observed broad I2 resonance of tfmF655-labeled FLN5 RNCs shares its chemical shift with the native state and, thus, demonstrates a native interresidue 655–675 tertiary contact and complete A–B strand pairing within this intermediate structure. Conversely, the random coil tfmF655 chemical shift of I1 (−61.8 ppm) indicates the absence of the native A–B strand contact (Fig. 1f,g).

**Extended Data Fig. 2:**
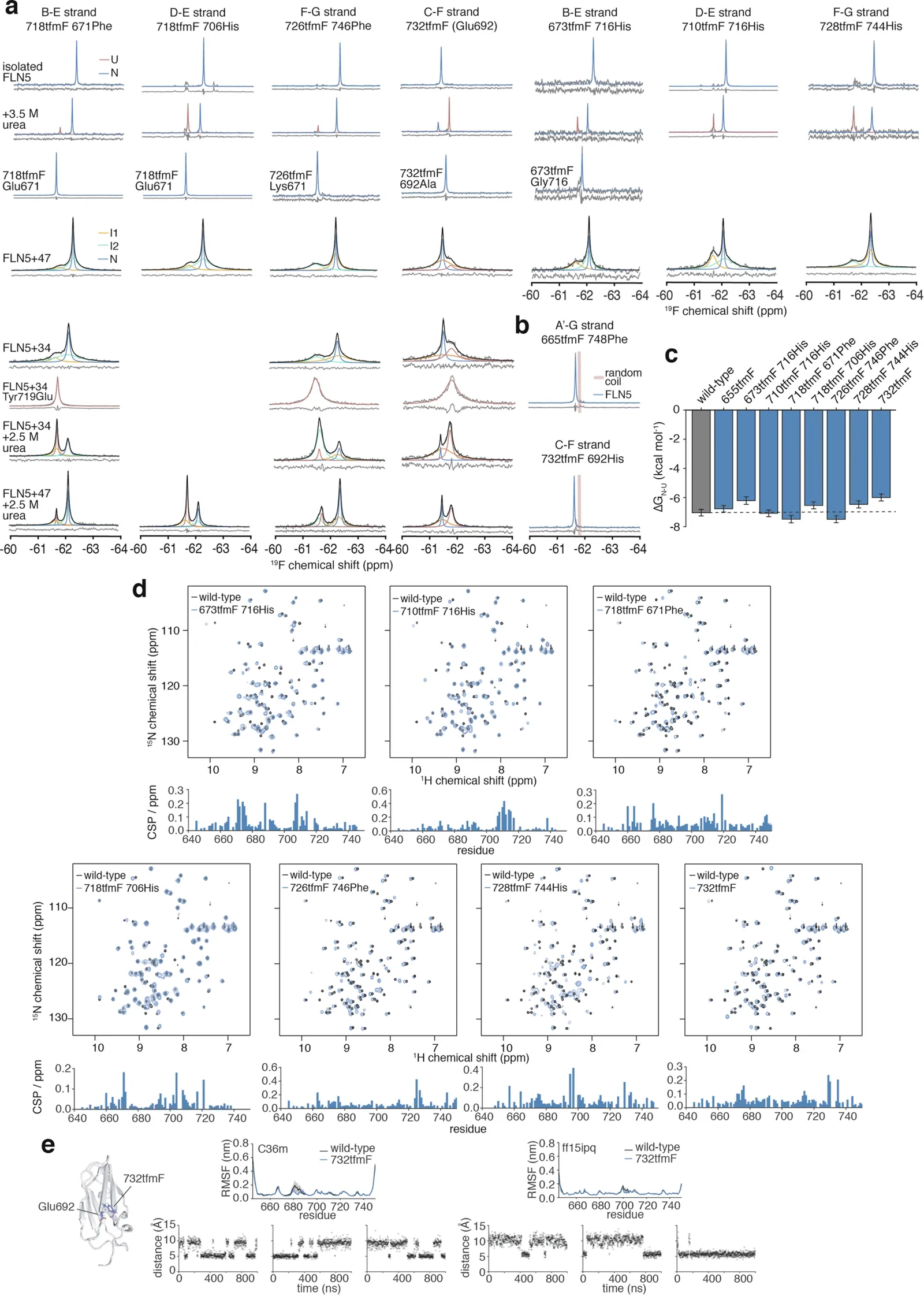
Characterisation of solvent-exposed tfmF-labelled variants of FLN5 on and off the ribosome. **a**, ^19^F NMR spectra of FLN5 tfmF-labelled at different positions, as isolated protein, with/without urea and with/without the accompanying aromatic label residue, and as RNC. Observed spectra shown in grey were fitted and assigned to their conformational states (coloured). Total fitted spectra are shown in black, and residual spectrum after fitting are shown below. Spectra were recorded at 500 MHz and 298 K. **b**, ^19^F NMR spectra of FLN5 tfmF-labelled at different positions. Red vertical line indicates random coil chemical shift range, and therefore the limited chemical shift dispersion of the variants. Observed spectra shown in grey were fitted (blue), with the residual spectrum after fitting shown below. Spectra were recorded at 500 MHz and 298 K. **c**, Gibb’s free energies of folding of different tfmF-labelled FLN5, determined by lineshape analyses of spectra shown in a. Errors were determined by bootstrapping of residuals from lineshape fits (mean ± s.e.m.). Values for wild-type and 655tfmF FLN5 were previously reported^2,11^. **d**, 2D ^1^H,^15^N-SOFAST HMQC spectra of tfmF-labelled FLN5 overlaid with that of wild-type FLN5. Chemical shift perturbations (CSPs) are shown below. Spectra were recorded at 500 MHz (wild-type at 800 MHz) and 298 K. **e**, Simulations of FLN5 732tfmF. Left shows a structural model highlighting the 732tfmF and Glu692 sidechains. Top middle/right shows average ( ± s.e.m.) C_α_-RMSF of wild-type and 732tfmF FLN5 simulated with C36m and ff15ipq parameters. Bottom middle/right shows distances between the centres of mass of tfmF CF_3_ and Glu692 carboxylic acid group, for 3 independent simulations using C36m and ff15ipq parameters. Source data

Additional naturally occurring ring currents are absent in FLN5 and tfmF labels at other solvent-exposed sites on FLN5 yield chemical shifts that are poorly resolved between the folded and unfolded (random coil) states^42^ and, thus, do not show clear correlations with (local) protein structure, limiting their use in structural studies. Using ColabFold^45^, AlphaFold3 (ref. ^46^) and all-atom MD simulations, we rationally designed^42^ five additional ^19^F-label pairs to engineer ring currents and probe specific side-chain contacts between pairs of β-strands, encompassing the B–E, D–E and F–G strands (Fig. 1h and Extended Data Fig. 2), akin to the introduction of (bulkier) Förster resonance energy transfer (FRET) or double electron–electron resonance probes. Efforts to generate label pairs for C–F and A′–G using the ring-current strategy resulted in more modest chemical shift dispersions insufficient to resolve broad RNC resonances (Extended Data Fig. 2b). The successful FLN5 label variants, in isolation of the ribosome, remained two-state folders and showed only mild changes to their folding free energies (∆∆*G*_N__–U_ < 0.8 kcal mol^−1^ relative to nonfluorinated; Extended Data Fig. 2c) and minimal perturbations to their ^1^H,^15^N-correlated NMR resonances (Extended Data Fig. 2d), being solvent exposed. Moreover, their mildly (de)stabilizing effect is expected to be largely suppressed on the RNC because of the ribosome’s mutation buffering effect (that is, changes in folding free energies are reduced on the ribosome; Supplementary Table 2)^3,29^. Their ^19^F chemical shifts were substantially (de)shielded (>0.2 ppm) from the random coil and highly sensitive to the presence of the introduced aromatic ring (Fig. 1h), as designed. Chemical shifts engineered using ring currents can, therefore, be interpreted as a residue-pair-specific van der Waals contact^42^.

We introduced each label into FLN5+47 RNC, the length at which the coTF intermediates are maximally populated^2^ (44% ± 3% and 38% ± 6% of I1 and I2, respectively, for FLN5+47 655tfmF; Fig. 1d). First, FLN5+47 RNC was labeled to probe the B–E strands (718tfmF 671Phe), yielding ^19^F NMR spectra with a sharp (~18-Hz linewidth) resonance at an identical chemical shift to that of the isolated protein (−62.2 ppm), corresponding to the native state as expected (Fig. 1d), and two additional broad resonances at the native and random coil (−61.8 ppm) chemical shifts attributable to intermediate states (Supplementary Note 1). The distinct chemical shifts, thus, indicate two populations of intermediates, one possessing and one lacking the native B–E strand contact. Similarly, labeling of FLN5+47 RNC at an alternative position across the B–E strands (673tfmF 716His) also produced two broad resonances, having native and random coil chemical shifts (Extended Data Fig. 2a) and suggesting that the presence or absence of native contacts persists across the interstrand network, respectively, thus completing secondary-structure formation.

When labeled to probe either the D–E (718tfmF 706His) or F–G (726tfmF 746Phe) strands, FLN5 RNCs also produced broad resonances attributable to two intermediate states (Fig. 1h). In each case, the broad resonances were found at both native and random coil chemical shifts, consistent with alternative label pairs positioned along the same strands (710tfmF 716His and 728tfmF 744His; Extended Data Fig. 2a and Supplementary Note 1). In addition to the findings for the A–B and B–E strands, native interstrand contacts across the D–E and F–G strands are, therefore, only found in one intermediate.

**Extended Data Fig. 3:**
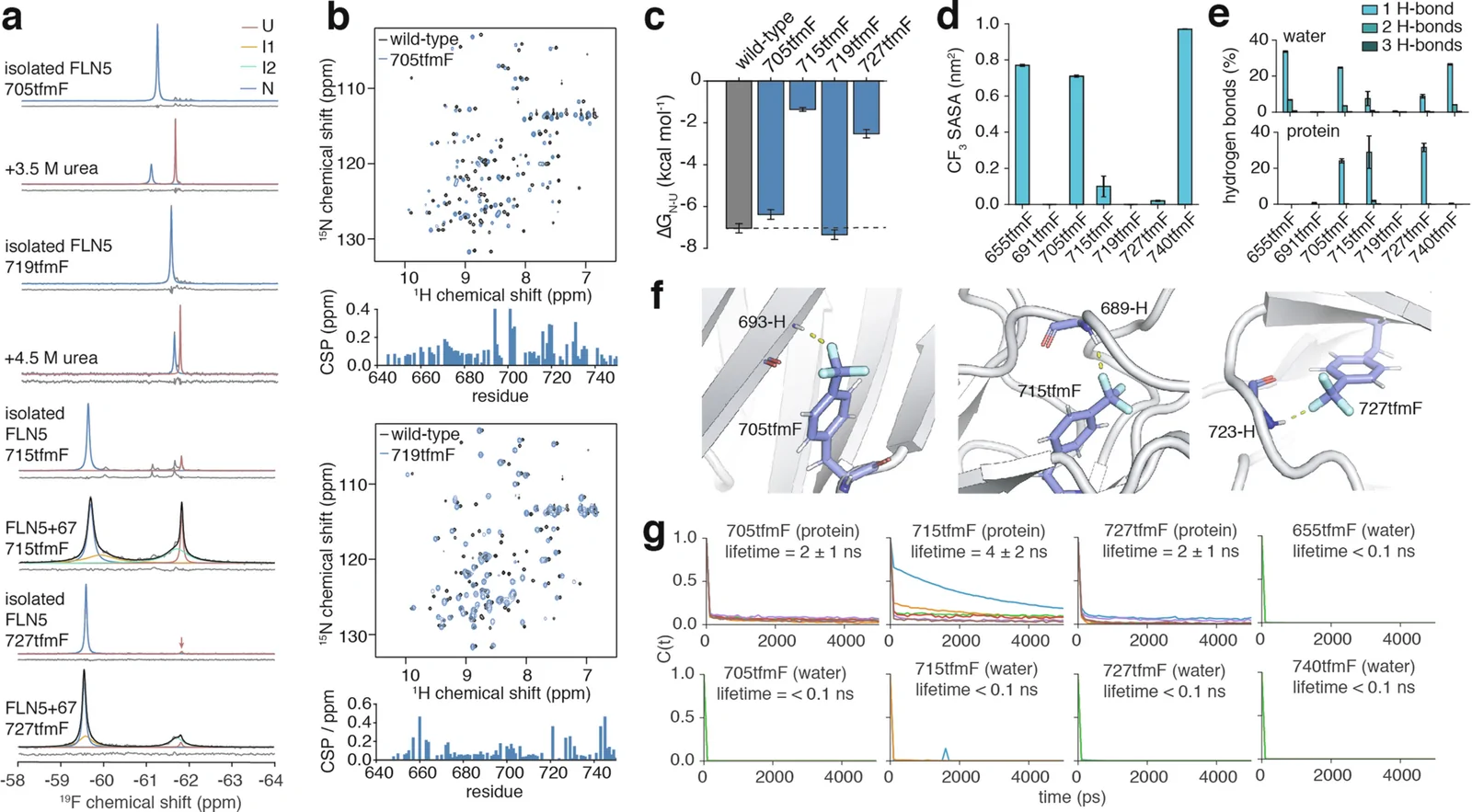
Characterisation of natively buried tfmF-labelled variants of FLN5 on and off the ribosome. **a**, ^19^F NMR spectra of FLN5 tfmF-labelled at different positions, as isolated protein, with/without urea and with/without the accompanying aromatic label residue, and as RNC. Observed spectra shown in grey were fitted and assigned to their conformational states (coloured). Total fitted spectra are shown in black, and residual spectrum after fitting are shown below. Spectra were recorded at 500 MHz and 298 K. **b**, 2D ^1^H,^15^N-SOFAST HMQC spectra of tfmF-labelled FLN5 overlaid with that of wild-type FLN5. Chemical shift perturbations (CSPs) are shown below. Spectra were recorded at 500 MHz (wild-type at 800 MHz) and 298 K. **c**, Gibb’s free energies of folding of different tfmF-labelled FLN5, determined by lineshape analyses of spectra shown in a. Errors were determined by bootstrapping of residuals from lineshape fits (mean ± s.e.m.). Value for wild-type FLN5 was previously reported^11^. **d-g**, Simulations and interactions of additional FLN5 tfmF sidechains with ff15ipq parameters. Solvent-exposed sites (655tfmF and 740tfmF) were used as controls. For wild-type, 655tfmF and 740tfmF, three independent simulations of 1 μs were performed. For 691tfmF, 705tfmF, 715tfmF, 719tfmF, and 727tfmF six simulations of 1 μs (and in three simulations water coordinates were saved for analysis). **d**, Solvent-accessible surface area of the CF_3_ group (mean ± s.e.m., n = 6) for all variants. **e**, Population of hydrogen bonds (H-bonds) between CF_3_ and water (n = 3), and between CF_3_ and protein atoms (n = 6), for all variants (mean ± s.e.m.). **f**, Most common hydrogen bond contacts formed by 705tfmF, 715tfmF and 727tfmF with amide groups of residues 693, 689 and 723, respectively. **g**, Averaged hydrogen bond existence autocorrelation functions and estimated lifetimes (mean ± s.e.m. or upper bound, from the area under the curve). Individual lines represent simulations replicates. Source data

The 732tfmF (F strand) chemical shift is highly sensitive to the electrostatic charge of the native neighboring residue Glu692 (C strand) in the isolated protein^42^ (Fig. 1h and Extended Data Figs. 1e and 2a). In addition to the native-state resonance, the ^19^F NMR spectrum of FLN5+47 732tfmF showed a single broad resonance (67% ± 11% population) observed at the same chemical shift that was attributable to an intermediate state (Extended Data Fig. 2a). Only a lowly populated (11% ± 4%) peak was observed at the random coil chemical shift, having a linewidth (Supplementary Table 1) and small population (Supplementary Table 2) consistent with an unfolded state, the latter as expected from the more destabilizing nature of the 732tfmF label (relative to other ^19^F-label pairs; Extended Data Fig. 2c), and its identity was confirmed by its observed increase in population in response to the addition of urea and comparison to an unfolded mutant RNC (Extended Data Fig. 2a). Similar results were found for the shorter FLN5+34 RNC (Extended Data Fig. 2a and Supplementary Note 1). These data indicate that both I1 and I2 intermediates possess a native 732–692 contact. Additional structural contacts in the hydrophobic core were measured with natively buried ^19^F labels (Extended Data Fig. 3).

Collectively, by using and engineering ^19^F chemical shifts that unambiguously probe specific intramolecular contacts, we resolved two distinct intermediate states in slow exchange with the natively folded state (Supplementary Note 1), both with native-like structure between the C–F strands and where additional native contacts across the remaining β-strand pairs and buried core are found in only one intermediate conformation.

## Engineering reversible crosslinks stabilizes I1

**Fig. 2:**
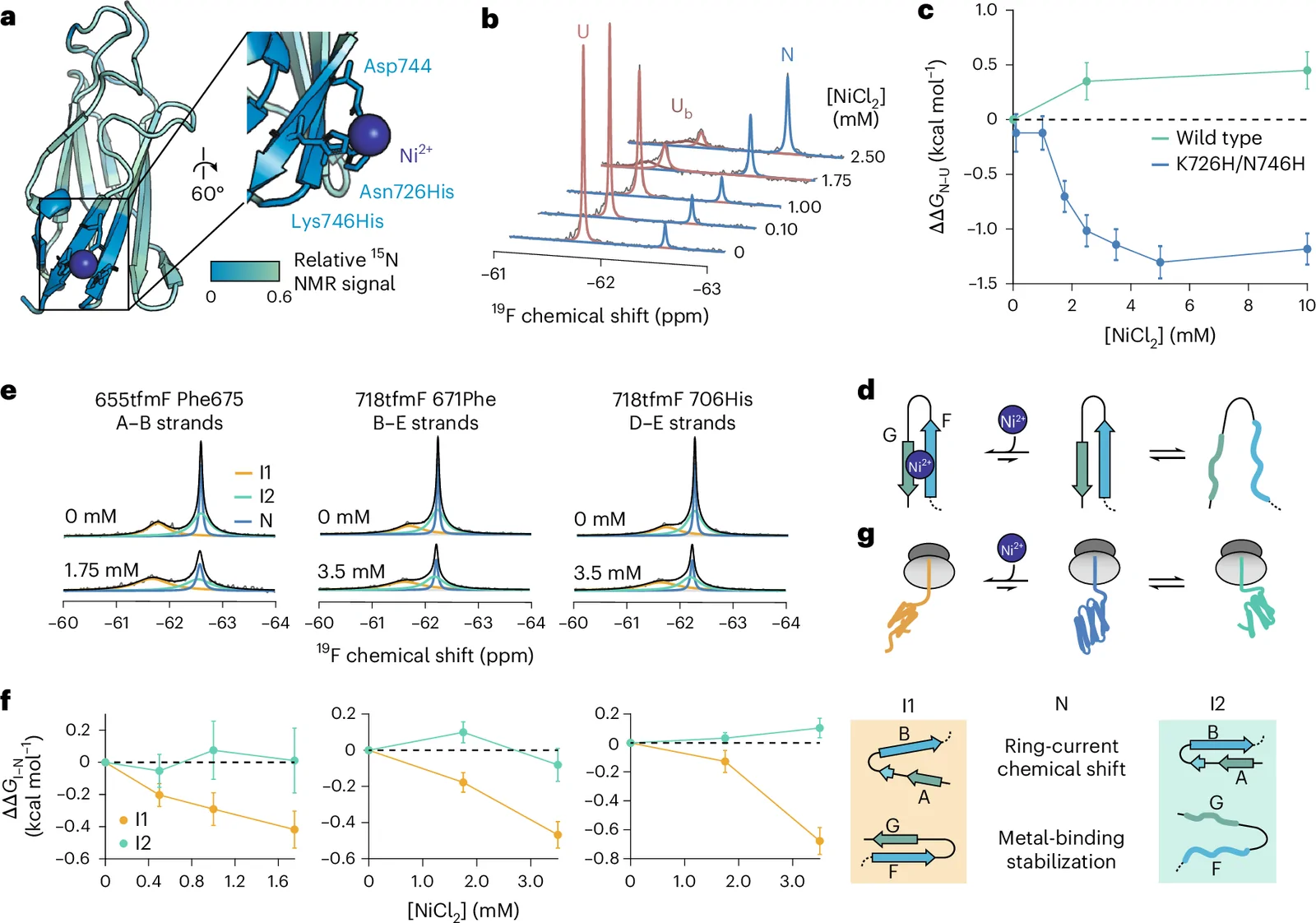
Selective stabilization of a specific coTF intermediate using an engineered ligand-binding site. **a**, AlphaFold3-predicted structure of FLN5 Asn726His Lys746His, highlighting metal ion coordination by the engineered histidine residues in the neighboring Asp744, and colored according to the ^1^H,^15^N-correlated NMR signal intensity in 2.5 mM NiCl_2_ relative to without NiCl_2_ (complete dataset in Extended Data Fig. 4c). **b**, ^19^F NMR spectra of 655tfmF-labeled FLN5 Asn726His Lys746His incubated in 2 M guanidine hydrochloride and varying NiCl_2_ concentrations. Observed and total fitted spectra are shown in gray and black, respectively. Unfolded (U), nickel-bound unfolded (U_b_; Extended Data Fig. 4g) and native (N) states are in color and indicated. **c**, Folding free energy changes of isolated wild-type and bihistidine FLN5 in varying NiCl_2_ concentrations (relative to without NiCl_2_), determined by lineshape analysis of spectra shown in **b** and Extended Data Fig. 4c. Errors were determined by bootstrapping of residuals from lineshape fits**. d**, Schematic of equilibrium reaction between unfolded and folded β-strand pairs titrated with Ni(II). **e**, ^19^F NMR spectra of tfmF-labeled FLN5+47 RNCs with Asn726His Lys746His, with and without 3.5 mM NiCl_2_ (complete titration data in Extended Data Fig. 4j). Observed and total fitted spectra are shown in gray and black, respectively. The assignment of resonances is colored accordingly. **f**, Changes in Gibbs free energies of intermediate relative to native state, determined by lineshape analysis of spectra shown in **e** and Supplementary Fig. 3j (complete titration data in Extended Data Fig. 4j). Errors were determined by bootstrapping of residuals from lineshape fits**. g**, Schematic of equilibrium reaction among I1, I2 and native states titrated with Ni(II). Nickel binding stabilizes I1 more than I2, inferring the presence and absence of the bihistidine-binding site, respectively. I1 and I2 are also distinguished by their ring-current-sensitive chemical shifts. All spectra were recorded at 500 MHz and 298 K. Unless stated otherwise, all values in the figure represent the mean ± s.e.m. propagated from NMR lineshape fits. Source data

**Extended Data Fig. 4:**
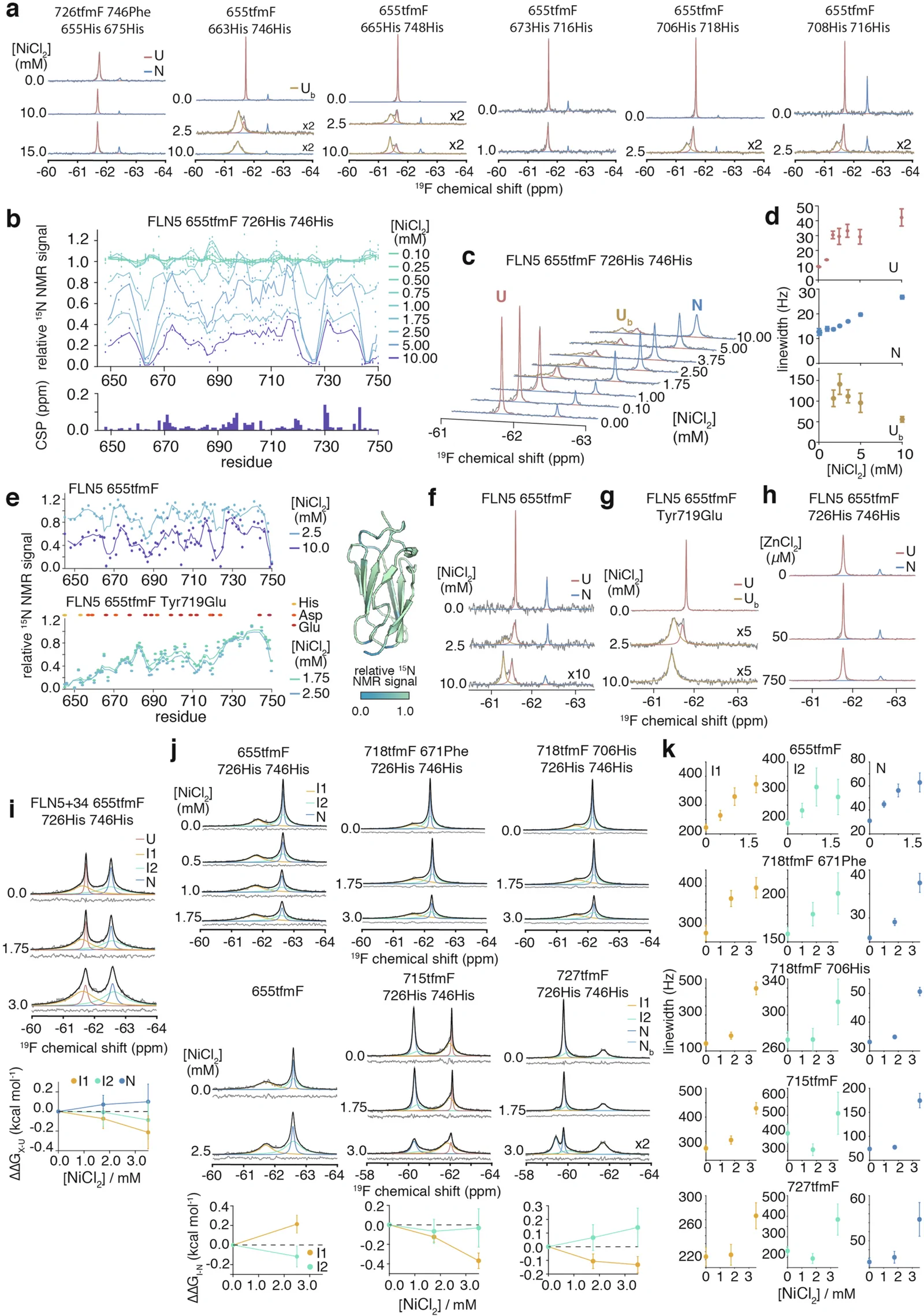
Engineering ligand binding sites to stabilise specific conformational states. **a**, ^19^F NMR spectra of isolated FLN5 with bi-histidine mutations at different positions (across different β-strands) and with varying concentrations of NiCl_2_. All show no substantial stabilisation of the natively folded state with NiCl_2_. **b**, Top shows residue-resolved ^1^H,^15^N-SOFAST HMQC signal intensities of FLN5 655tfmF 726His 746His in varying NiCl_2_ concentrations relative to without NiCl_2_. Bottom shows ^1^H,^15^N-chemical shift perturbations of FLN5 655tfmF 726His 746His in 10 relative to 0 mM NiCl_2_. **c**, ^19^F NMR spectra of isolated FLN5 655tfmF 726His 746His with varying concentrations of NiCl_2_. **d**, Linewidths determined by lineshape analysis of spectra shown in c. Errors were determined by bootstrapping of residuals from lineshape fits. **e**, Residue-resolved ^1^H,^15^N-SOFAST HMQC signal intensities of FLN5 655tfmF (without bi-histidine mutations, 2.5 mM dataset mapped onto the FLN5 crystal structure, PDB 1QFH, right) and FLN5 655tfmF Tyr719Glu (the latter unfolded^39^) in varying NiCl_2_ concentrations relative to without NiCl_2_. **f**, ^19^F NMR spectra of isolated FLN5 (without bi-histidine mutations) in varying NiCl_2_ concentrations. **g**, ^19^F NMR spectra of isolated FLN5 Tyr719Glu (without bi-histidine mutations, and unfolded^39^) in varying NiCl_2_ concentrations. **h**, 1D ^19^F NMR spectra of isolated FLN5 726His 746His in varying ZnCl_2_ concentrations. **i**, ^19^F NMR spectra of FLN5+34 tfmF655tfmF 726His 746His RNC in varying NiCl_2_ concentrations, bottom shows Gibb’s free energies calculated using lineshape analyses of spectra. Errors were determined by bootstrapping of residuals from lineshape fits. **j**, As in i, but for FLN5+47 with different ^19^F-labelled and non-fluorinated variants. **k**, Linewidths determined by lineshape analysis of spectra shown in j. Errors were determined by bootstrapping of residuals from lineshape fits. For all ^19^F NMR spectra, observed spectra shown in grey were fitted and assigned to their conformational states (coloured). Total fitted spectra are shown in black, and residual spectrum after fitting are shown below. All spectra were recorded at 500 MHz and 298 K. Unless stated otherwise, all values in the figure represent mean ± s.e.m. propagated from NMR lineshape fits. Source data

We next aimed to assign the broad resonances to either I1 or I2 to generate a dataset of chemical shifts (that is, structural contacts) specific to either coTF intermediate. Thus, we sought to selectively (de)stabilize one intermediate conformation using principles of psi-value analysis^47^, introducing reversible crosslinks by engineering bihistidine metal-ion-binding sites to stabilize specific β-strands. Of the seven mutants tested, one variant, 726His 746His (F–G strands), showed a progressive stabilization of up to −1.45 kcal mol^−1^ in its folding free energy with increasing concentrations of NiCl_2_ (Fig. 2a–c and Extended Data Fig. 4a–c). In the absence of NiCl_2_, its stability was similar to that of the wild-type protein (Fig. 2c). The metal-binding site was confirmed by ^1^H,^15^N NMR, where addition of the paramagnetic Ni(II) metal resulted in increased line broadening localized at the substitution sites (Fig. 2a and Extended Data Fig. 4b–h), in accordance with AlphaFold3 predictions (Fig. 2a).

Having designed a conformation-specific, concentration-dependent ligand-binding site (Fig. 2d), we next introduced the bihistidine substitutions into FLN5+47 RNCs (Fig. 1h and Extended Data Fig. 4i–k). FLN5+47 tfmF655 RNCs (probing A–B strands; Fig. 1f) yielded resonances corresponding to I1, I2 and native states as expected (Fig. 2e). Addition of increasing amounts of Ni(II) to the RNC sample resulted in increasingly broader resonances, as anticipated from the metal’s paramagnetic effect (Extended Data Fig. 4k), but critically also a progressive shift in the folding equilibrium toward the I1 state (Fig. 2e,f and Extended Data Fig. 4j) only in the presence of the bihistidine substitutions (Extended Data Fig. 4j). An analysis of the populations and Gibbs free energies of each state revealed that I1 was stabilized more than I2 (each relative to N; Fig. 2f). Given the distinct chemical shifts obtained for the F–G strand ^19^F labels (Fig. 1h and Extended Data Fig. 4a), this indicated the presence of a folded binding site across the F–G strands in I1 that was likely absent in I2 (Fig. 2g). Therefore, I1 possesses a native F–G strand conformation but lacks A–B strand contacts (Fig. 2g), whereas I2 exhibits the opposite.

The bihistidine site was introduced in FLN5+47 RNCs possessing the remaining ^19^F-label pairs, all resulting in only minimal perturbations in the thermodynamic stabilities of both intermediates (relative to the native state; ∆∆*G*(mut-wt, I1–N) = 0.3 ± 0.1 kcal mol^−1^ and ∆∆*G*(mut-wt, I2–N) = 0.0 ± 0.2 kcal mol^−1^) and their ^19^F chemical shifts at solvent-exposed (<0.02 ppm) and solvent-inaccessible (<0.15 ppm) sites. When incubated with Ni(II), all variants showed two distinct intermediate resonances (Fig. 1h and Extended Data Fig. 2a), with one intermediate (I1) consistently stabilized more than the other (I2) (Fig. 2e,f and Extended Data Fig. 4j). These data permitted us to assign the chemical shifts across all the tfmF labels to either the I1 or I2 intermediate (Fig. 1g and Extended Data Fig. 4j). Collectively, they show that I1 likely possesses a natively folded sheet 2 (CFG strands) with native contacts absent across sheet 1 (ABED strands). However, native-like strands are likely to be at least partially formed within sheet 1, as I1 is completely destabilized by the natively buried Tyr719Glu substitution in the core between the two β-sheets^2^. Meanwhile, on the basis of the ^19^F NMR contacts, I2 appears to closely resemble the intermediate found in isolated C-terminally truncated variants of FLN5 (ref. ^11^), comprising a natively folded A–F strand structure with a disordered C-terminal G strand; accordingly, native-like chemical shifts for the natively buried sites tfmF715 and tfmF727 also correspond to the I2 intermediate (attributed using the same bihistidine-binding approach; Extended Data Fig. 3a,j,k).

## Structures of folding intermediates on the ribosome

**Fig. 3:**
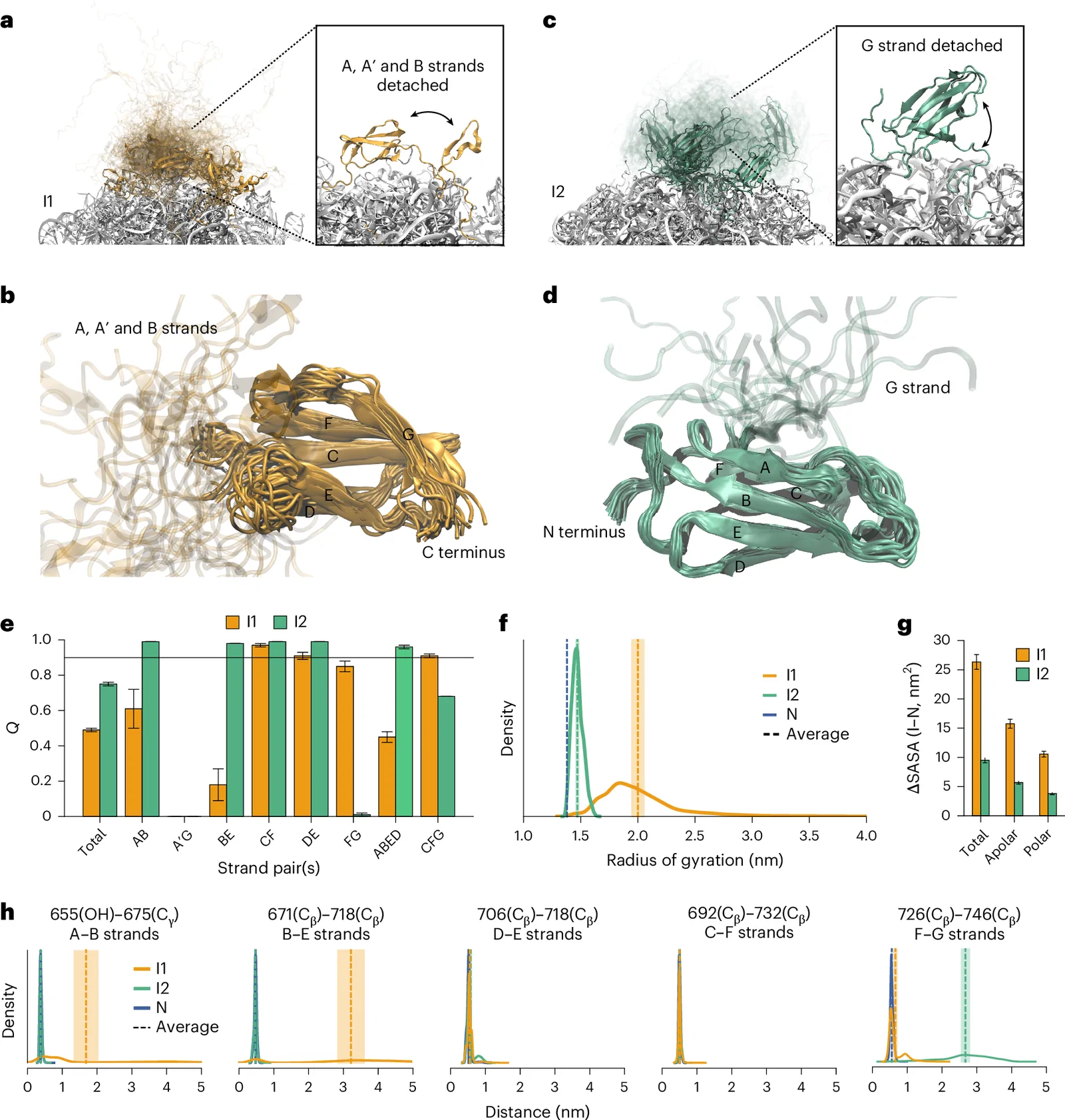
Atomistic structures of FLN5+47 coTF intermediates on the ribosome. **a**, Structural ensemble of FLN5+47 I1 on the ribosome. Inset, representative structure. **b**, Lowest-energy structural cluster of I1 (Supplementary Table 4) aligned against the common C–G strand core. **c**, Structural ensemble of FLN5+47 I2 on the ribosome. Inset, representative structure. **d**, Lowest-energy structural cluster of I2 (Supplementary Table 4) aligned against the common A–F strand core. **e**, Average fraction of native contacts (Q) of FLN5 and across different β-strand pairs and β-sheets for I1 and I2 (mean ± s.e.m. from *n* = 15 independent simulations of 1 µs). **f**, Radius of gyration probability distribution and mean ± s.e.m. of I1, I2 and native states. **g**, Difference in SASA (mean ± s.e.m.) of I1 and I2 (*n* = 15) relative to the native state (*n* = 8). **h**, Distance distributions and mean ± s.e.m. (*n* = 15) of residue side chains corresponding to ^19^F-label pairs calculated for structural ensembles in I1, I2 and native states. Source data

**Extended Data Fig. 5:**
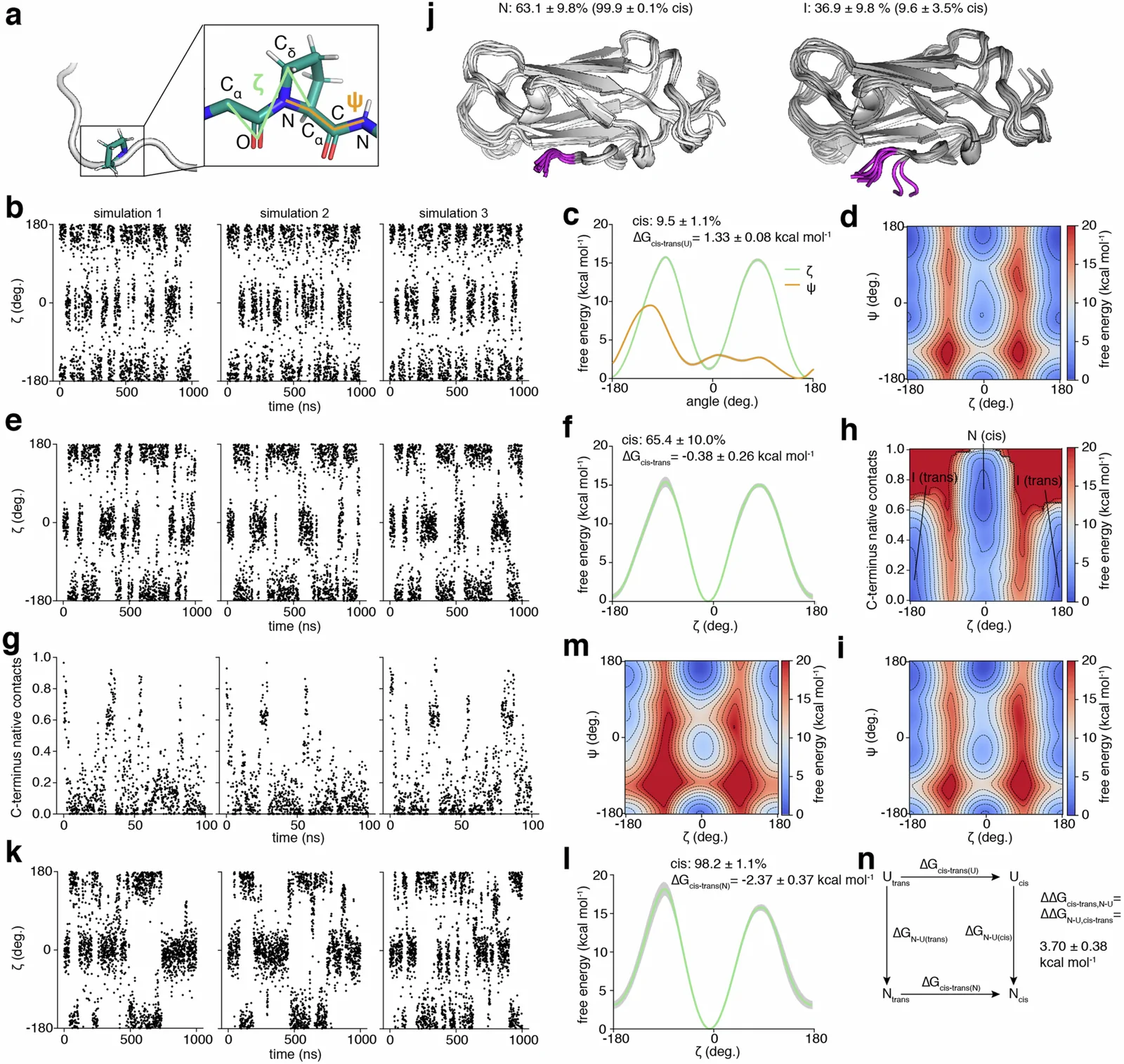
Structure-dependent proline isomerisation of FLN5 Pro742. **a**, Dihedral angles ζ and ψ used as collective variables for enhanced sampling simulations with WT-METAD. **b**, Sampling of ζ in three independent WT-METAD simulations of the unfolded peptide. **c**, Reweighted free energy landscape of Pro742 projected onto ζ and ψ, respectively, for the unfolded peptide (mean ± s.e.m., n = 3). **d**, Reweighted 2D free energy landscape of P742 projected onto ζ and ψ for the unfolded peptide. **e**, Sampling of ζ in three independent WT-METAD simulations of FLN5Δ6. **f**, Reweighted free energy landscape of P742 projected onto ζ for FLN5Δ6 (mean ± s.e.m., n = 3). **g**, Sampling of C-terminal native contacts (fraction of native contacts formed by residues I743 and D744) in three independent WT-METAD simulations of FLN5Δ6. **h**, Reweighted 2D free energy landscape of FLN5 Δ6 projected onto ζ and CTERM averaged across all three simulations. The energy minima corresponding to the native (N) and intermediate (I) state are annotated. **i**, Reweighted 2D free energy landscape of FLN5 Δ6 projected onto ζ and ψ averaged across all three simulations. **j**, Structural models (10 lowest free energy conformations) corresponding to FLN5Δ6 N and I states and their equilibrium populations predicted by WT-METAD including their P742 cis populations. The C-terminal residues Ile743 and Asp744 are shown in magenta. **k**, Sampling of ζ in three independent WT-METAD simulations of full-length, folded FLN5. **l**, Reweighted free energy landscape of full-length FLN5 projected onto ζ (mean ± s.e.m., n = 3). **m**. Reweighted 2D free energy landscape of full-length FLN5 projected onto ζ and ψ averaged across all three simulations. **n**. Thermodynamic cycle of the free energy changes of folding (ΔG_N-U_) and P742 isomerisation (ΔG_cis-trans_). The change in folding free energies (ΔΔG_N-U,cis-trans_) is equivalent to the change in isomerisation energies of the unfolded (U) and native (N) state (ΔΔG_N-U,cis-trans_). Using the value of the isomerisation free energies for U and N computed in panels c and l, we obtain a value of 3.70 ± 0.38 kcal mol^−1^. This is quantitatively consistent with the experimental value (ref. ^11^), 4.00 ± 0.10 kcal mol^−1^, for ΔΔG_N-U_ for the P742A mutant (with A742 in trans).

**Extended Data Fig. 6:**
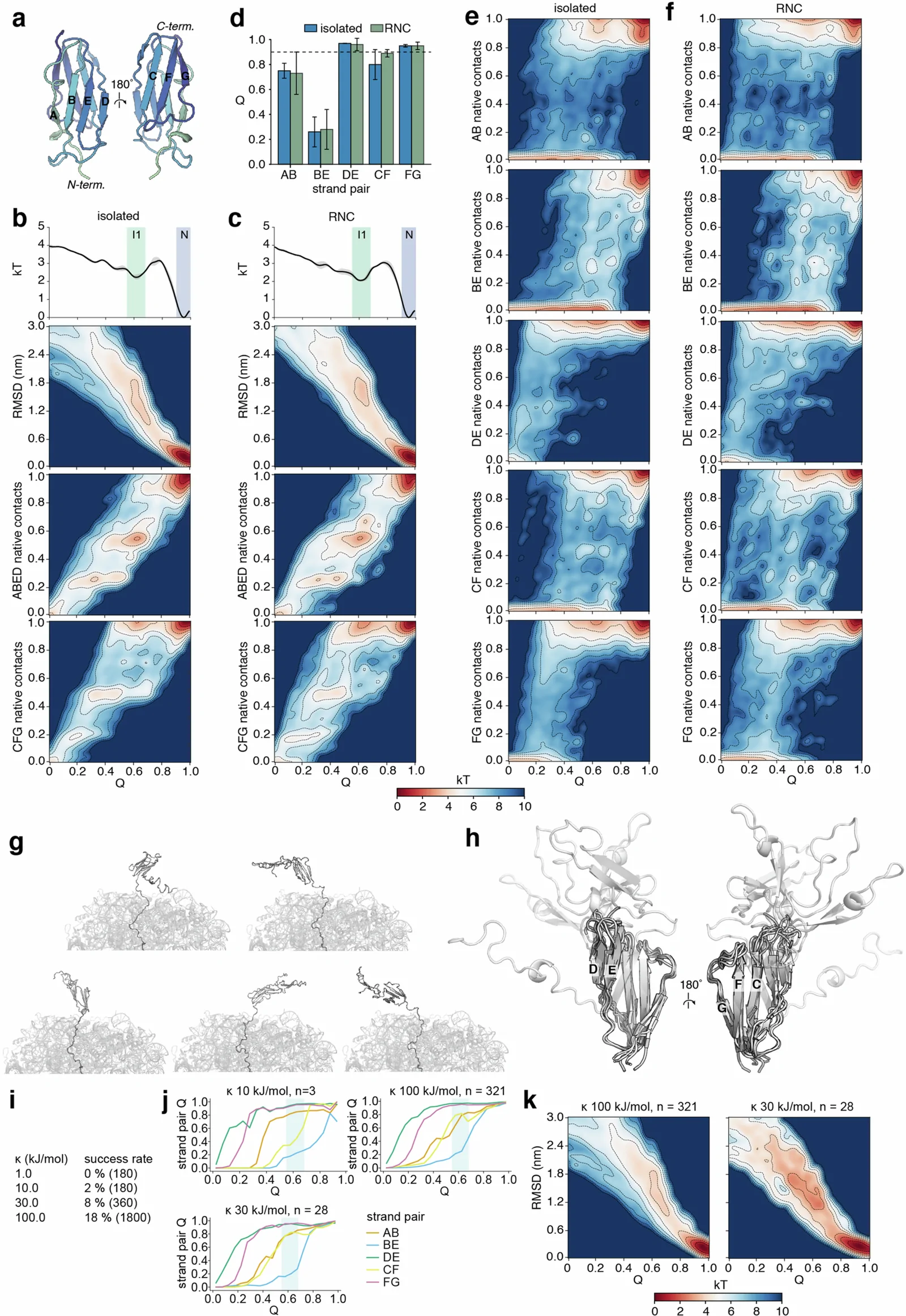
All-atom folding simulations of FLN5 using rMD. **a**, FLN5 crystal structure (PDB 1QFH) annotated with the main β-strands. **b-c**, Transition path free energy landscape of isolated FLN5 (b) and FLN5+47 RNC (c) as a function of the fraction of native contacts (Q), all-atom RMSD with respect to the native structure, and native contacts in sheet 1 (ABED) and 2 (CFG). The 1D energy plot shows the mean and error, estimated as the difference between the first and second half of the dataset (n = 2). 321 and 226 folding trajectories obtained from rMD simulations were used for the analysis of isolated FLN5 and FLN5+47, respectively. **d**, Average fraction of native contacts calculated for structures attributed to the folding intermediate, I1, energy minimum. Error bars were estimated as the difference between the first and second half of the dataset (n = 2). **e-f**, Transition path free energy landscape of isolated FLN5 (e) and FLN5+47 RNC (f) projected onto the native contacts of the main β-strand pairs. **g**, Five structural models of I1 on the ribosome, selected for unbiased MD simulations (see Methods). **h**, Aligned structural models of I1 using residues 685–750 (strands C to G) showing the folded core of I1 and disordered N-terminus (transparent, strands A to B). **i**, Table summarising the tested force constants (κ) and observed folding success rates for isolated FLN5 (total number of trial trajectories given in parentheses). **j**, Average fraction of strand pair contacts (strand pair Q) as a function of global folding progress (Q) for different force constants. Region of Q corresponding I1 (identified with κ = 100 kJ / mol, **b**) is shaded. **k**, Transition path free energy landscape of isolated FLN5 for two different force constants as a function of Q and the all-atom RMSD with respect to the native structure. Source data

**Extended Data Fig. 7:**
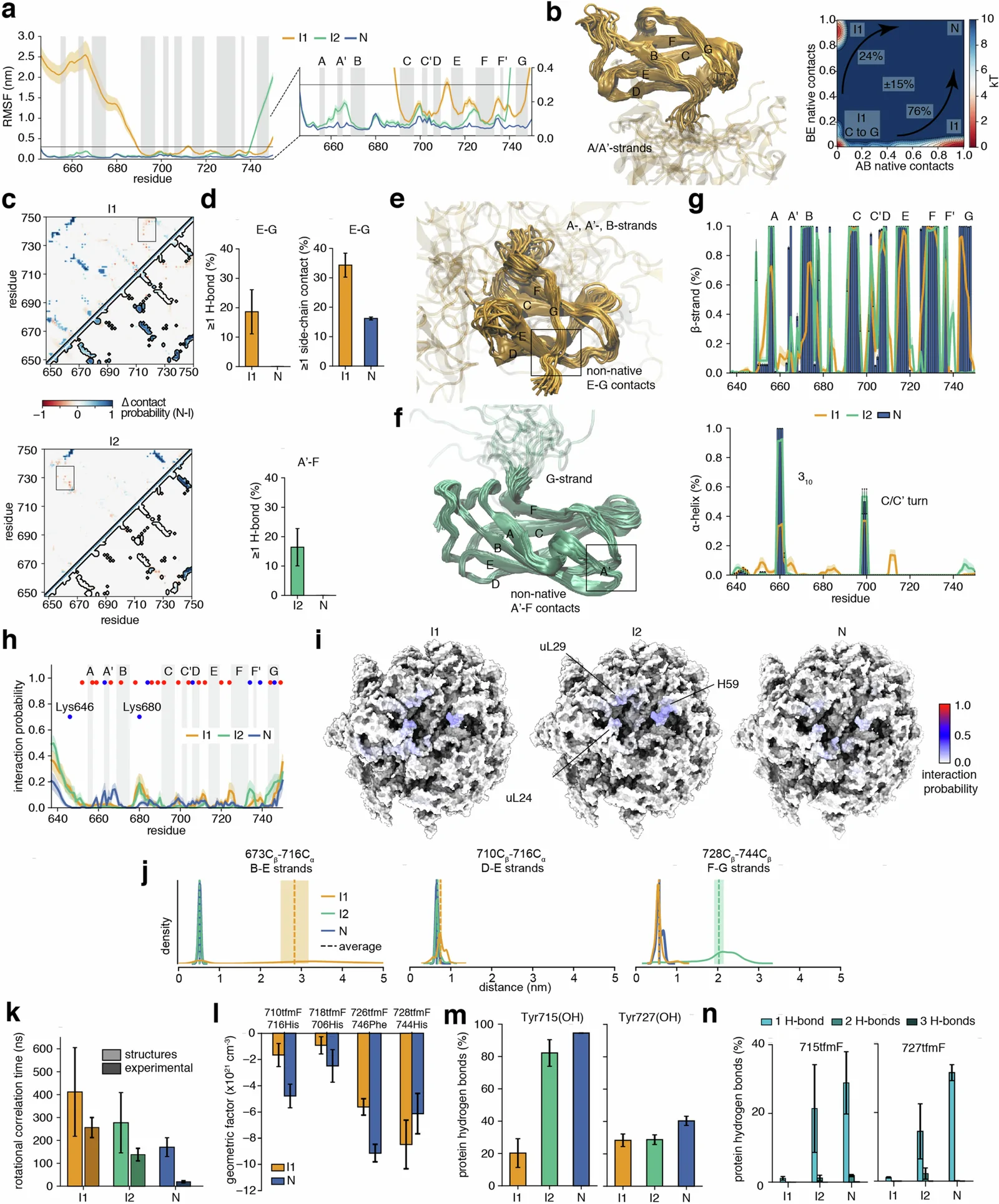
Structures, dynamics, and interactions of I1 and I2 coTF intermediates. **a**, Root mean square fluctuations (RMSF, measured at C_α_ atoms) across the sequence of FLN5 (mean ± s.e.m. from n = 15 independent simulations for I1/I2 and n = 8 independent simulations for N) and comparing all conformational states. Secondary structure regions (β-strands) of native FLN5 are annotated. **b**, (left) Structures of I1 from the lowest free energy structural cluster aligned using the common core of strands C to G where the B-strand is also associated with the core. (right) Energy landscape of I1 correlating native contacts between strands A-B (AB) and B-E (BE), showing two parallel folding routes towards the N state. The relative populations of these pathways were estimated from the rMD transition path ensemble (errors represent the difference between the first and second half of the dataset). **c**, Difference contact probability of I1 and I2 compared to N (N-I) highlighting loss of native (blue) and gain of non-native (red) contacts. The black contours represent the native contacts calculated from the crystal structure. Contacts were defined as heavy atom distances of less than 0.5 nm. Two regions in the box highlight non-native contacts (C-G strand contacts in I1 and A’-F strand contacts in I2). **d**, Probability (mean ± s.e.m.) of hydrogen bonds and sidechain contacts (0.35 nm heavy atom cut-off) between non-native structural elements (highlighted in c) compared to the native state. **e**, Representative structures of I1 showing non-native C-G strand contacts. **f**, Representative structures of I2 showing non-native A’-F strand contacts. **g**, Secondary structure propensity of coTF intermediates (lines) compared to the native state shown with bars (mean ± s.e.m.). **h**, Ribosome interaction probability (mean ± s.e.m.) shown along the sequence of FLN5, with native β-strands annotated. Negatively and positively charged residues are denoted with red and blue circles, respectively. **i**, FLN5 (residues 646–750) interactions mapped onto the ribosome surface for all conformational states. **j**, Distance distributions and mean ± s.e.m. of residue sidechains corresponding to ^19^F-label pairs calculated for I1, I2 and native state structural ensembles. **k**, Light bars show rotational correlation time of FLN5 calculated for all conformational states (mean ± s.e.m.), defined using the natively folded regions for I1 and I2. Dark bars show rotational correlation time calculated using experimental linewidths and taken from ref. ^3^. **l**, Ring current geometric factor (1–3cos^2^*θ*)/*r*^3^) (de)shielding contribution to the ^19^F chemical shift of probes reporting on strand pair contacts calculated from MD simulations of the native and I1 state using isolated proteins (mean ± s.e.m.). **m**, Tyr-OH hydrogen bonding probability calculated for all conformational state ensembles (mean ± s.e.m.). **n**, Population of hydrogen bonds (H-bonds) between CF_3_ and protein atoms calculated using isolated protein MD simulations (mean ± s.e.m.). Source data

Next, we aimed to convert our topological observations into detailed structures, by producing atomistic structural ensembles of the FLN5+47 RNC intermediate states. The structural similarity of contacts identified experimentally for I2 to the topology of isolated, truncated intermediate enabled us to produce a starting model of the RNC by attaching the previously determined structure of the latter^11^ to the ribosome (Supplementary Note 2). However, in the absence of any analogous structures to I1, we sought to simulate coTF from the unfolded to natively folded state using an all-atom (including ribosome, ions and solvent) ratchet-and-pawl MD (rMD) approach^48–50^ (Supplementary Note 2, Supplementary Videos 1 and 2 and Extended Data Figs. 5 and 6). The rMD simulations enabled us to successfully capture folding events, which ^19^F chemical exchange saturation transfer (CEST) NMR experiments show to likely occur on the timescale of >170 ms (Extended Data Fig. 1b,c), beyond the reach of conventional MD simulations, to produce a series of potential folding pathways. From the obtained coTF trajectories, we identified one on-pathway folding intermediate state (Extended Data Fig. 6), which, from our experimental dataset, appeared to possess structural features common to that of I1. To assess this further, we subsequently subjected the putative I1 and I2 models to extensive atomistic unbiased MD simulations (Extended Data Fig. 7), reducing the impact of rMD bias potentials on the final structural ensemble^51^. We found their folded regions to be stable on the microsecond timescale (Fig. 3 and Supplementary Videos 3 and 4) and, therefore, quantitatively compared the atomistic structural ensembles to those previously obtained for the natively folded RNC^23^.

The structures of the putative I1 state (Fig. 3a,b and Extended Data Fig. 7a–g) possess a native-like C–G strand folded core (Fig. 3e,f); the remaining N-terminal A, A′ and B strands are largely detached from the structure and form either a completely disordered tail (Supplementary Video 1) or a β-hairpin (Fig. 3e, Extended Data Fig. 7b and Supplementary Video 2). The loss of the A–B strands from the folded structure results in transient nonnative contacts, predominantly between the β-sheets at the exposed E–G and A′–F strand interfaces (~20–30%; Extended Data Fig. 7c–e). However, we also find a subpopulation (~24%) where the B strand folds onto the C–G core, with the A–A′ strands remaining disordered (Extended Data Fig. 7b); this conformational heterogeneity suggests multiple accessible pathways from I1 to the native state, as also reflected in our folding simulations (Extended Data Fig. 6), although these two I1–N routes cannot be resolved by our experiments. Overall, detachment of the N-terminal strands causes an expansion of the structural ensemble, with a larger radius of gyration and increased solvent-accessible surface area (SASA) compared to the native state (Fig. 3g). Structures of the putative I2 state (Fig. 3c,d and Extended Data Fig. 7a–g) adopt a more ordered and compact native-like conformation, with a smaller increase in SASA (Fig. 3f,g). Native contacts are found across the A–F strands (Fig. 3e), with a nonnative *trans*-Pro742 and disordered G strand (residues 743–747) detached from the rest of the domain permitting some nonnative interactions between A′ and F strands (Extended Data Fig. 7c–f). We note that energetic calculations show that native G strand folding is dependent on the *cis*–*trans* isomerization state of Pro742 (Supplementary Note 2 and Extended Data Fig. 5).

To assess whether the obtained structures faithfully represent our experimental data (Figs. 1 and 2), we examined their intramolecular contacts and compared them to the obtained ^19^F chemical shifts, reporting on precise, short-range interresidue distances^42^. We calculated distance distributions between the pairs of residues labeled for NMR analysis (Methods, Fig. 3h and Extended Data Fig. 7j) and found that all could account for the observed chemical shifts of I2; those with a native chemical shift showed short (~0.5 nm), native-like interresidue distances, consistent with the measured van der Waal’s contact, whereas residues labeling the F–G strands were distanced far (>2 nm) from each other, as reflected by their random coil chemical shifts (Fig. 1f,h and Extended Data Fig. 2a). Analyses for I1 also showed good agreement between interresidue distances and their ring-current-induced chemical shifts (Fig. 3d and Extended Data Fig. 7j). The exceptions were the two D–E strand labels, which could instead be resolved using more precise, explicit modeling of the ^19^F-label pair; the D–E strand pairs form native backbone contacts but lack side-chain interactions in line with the random coil chemical shifts found in this region (Supplementary Note 3 and Extended Data Fig. 7l). Likewise, such simulations entirely validate residue contacts in the core of the domain (Supplementary Note 3 and Extended Data Fig. 7m,n). In summary, our residue contact analyses show that both I1 and I2 structures quantitatively account for all of our ^19^F chemical shift data and, thus, that the rMD folding simulations captured an intermediate conformation corresponding to I1.

## Interactions and orientations of intermediates with the ribosome

**Extended Data Fig. 8:**
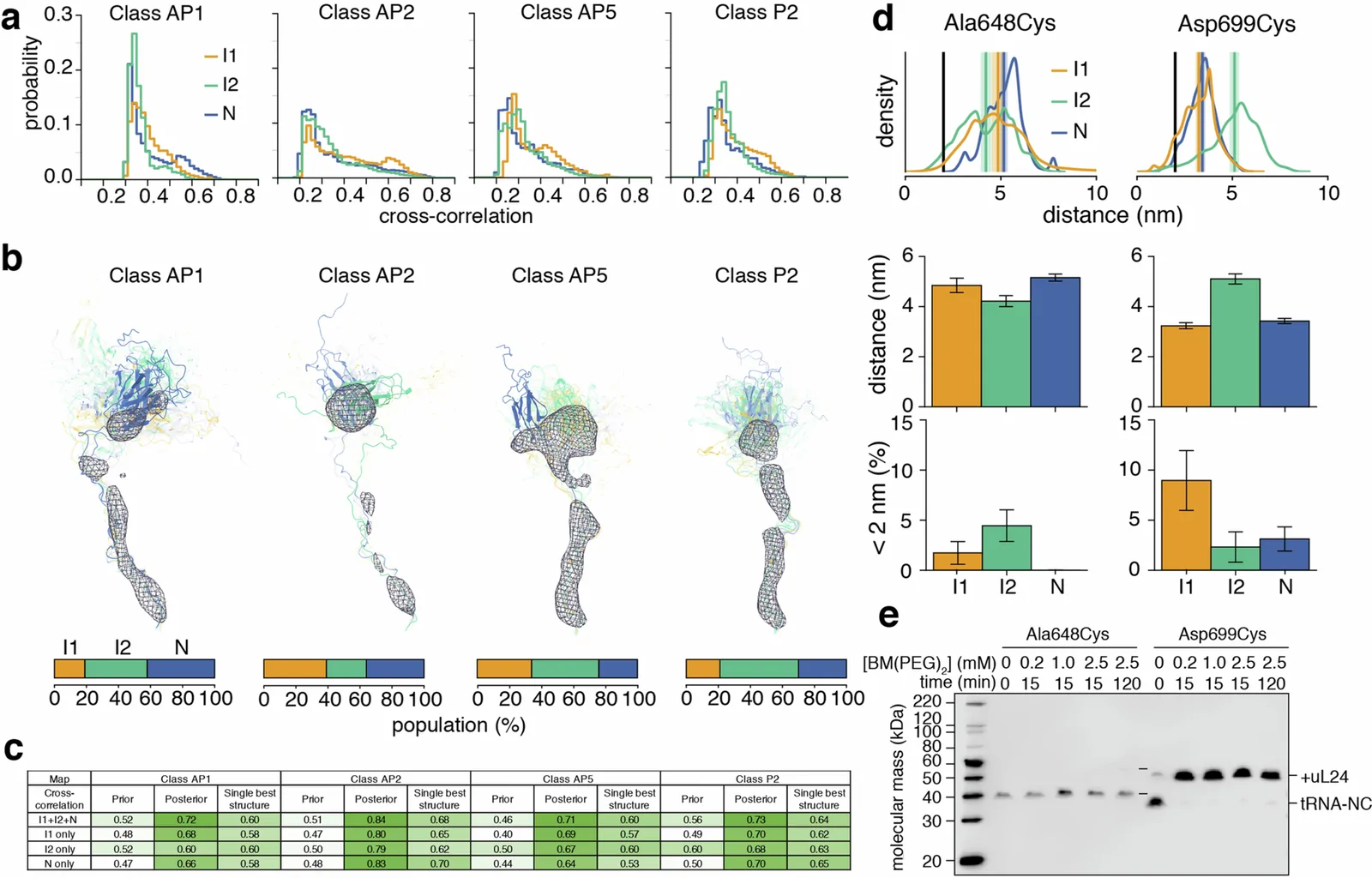
Validation of inter-molecular interactions and orientations of structural ensembles. **a-c**, Cryo-EM fitting and reweighting of structural ensembles. **a**, Probability distributions of the correlation coefficients obtained by fitting single conformations from all-atom MD trajectories of the native, I1, and I2 states to one of four cryo-EM maps, corresponding to the AP1, AP2, AP5 and P2 classes of FLN5+47 (Methods). **b**, Bayesian reweighting of the collective I1, I2 and N trajectories using the cryoEM maps of FLN5+47, obtained by cryoENsemble^52^. The reweighted structural ensembles are shown, coloured according to their conformational state, and their opacity set to their populations. Total posterior populations of each state are plotted in the bar chart. **c**, Cross-correlations obtained for cryoENsemble reweighting shown in b. We note that high cross-correlations of individual states do not necessarily correlate with their populations in the combined reweighting. States which agree only moderately with the cryo-EM map individually can still be in good agreement with the maps when averaged together. **d-e**, Cross-linking experiments of FLN5+47 RNC on uL24 Asn53Cys ribosomes. **d**, Top shows probability distributions and averages (mean ± s.e.m., vertical lines) for all distance pairs (uL24 Asn53 with cysteine residue on FLN5) and conformational states calculated from structural ensembles, middle shows averaged distances (mean ± s.e.m. from n = 15 independent simulations for I1/I2 and n = 8 independent simulations for N), and bottom shows probability of contacts using a cut-off of 2 nm. **e**, Anti-hexahistidine detected western blot of FLN5+47 cysteine variant (pseudo wild-type Cys747Val, Methods) cross-linked to uL24 Asn53Cys using BM(PEG)_2_ (Methods). Ribosome-bound nascent chain (covalently linked to its tRNA, labelled tRNA-NC) and same but covalently linked to uL24 are indicated. Source data

The intermediate-state structures show transient interactions with the ribosome surface, predominantly with ribosomal proteins uL24, uL29 and RNA H59 lining the vestibule of the exit tunnel and more favorably than the native state (Extended Data Fig. 7h,i). The dynamics of these interactions are consistent with line broadening of their ^19^F NMR resonances^2,3^ and the interaction sites and orientations were found to be in accord with both chemical crosslinking and ensemble-reweighting^52^ analyses of cryo-electron microscopy (cryo-EM) maps of FLN5+47 RNC^53^ (Extended Data Fig. 8 and Supplementary Note 4).

## ^19^F paramagnetic relaxation enhancement (PRE) NMR measurements validate intermediate structures

**Fig. 4:**
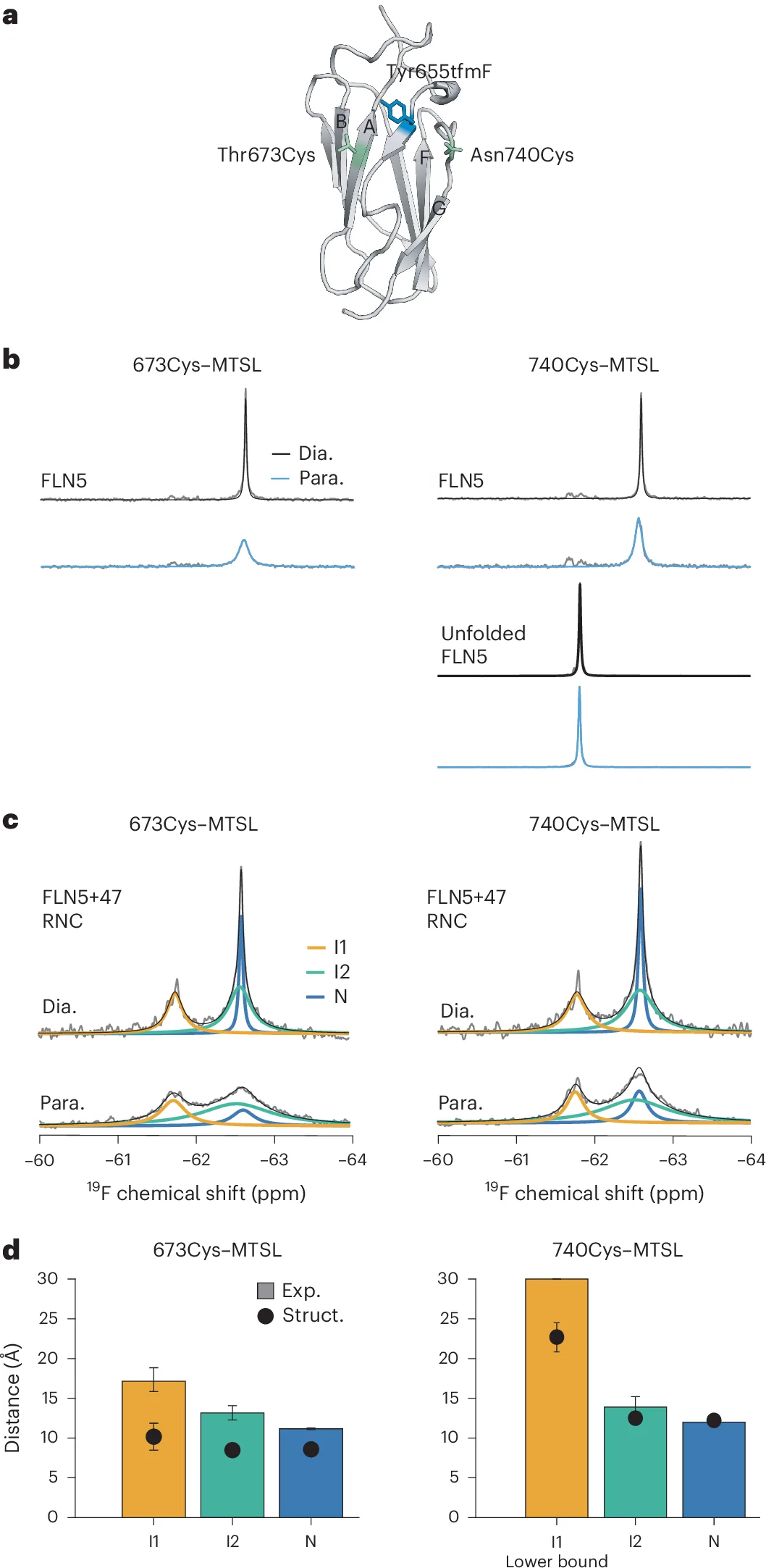
Validation of coTF intermediate structures by ^19^F PRE NMR. **a**, Location of tfmF and paramagnetic spin labels shown on the FLN5 crystal structure (PDB 1QFH). **b**, ^19^F NMR spectra of isolated 655tfmF-labeled FLN5 (pseudo-wild-type Cys747Val (Methods) and unfolded with destabilizing substitutions (A_3_A_3_)^39^, spin-labeled at positions 673Cys (left) and 740Cys (right). Observed spectra are shown in gray. **c**, ^19^F NMR spectra of 655tfmF-labeled FLN5+47 RNC (pseudo-wild-type Cys747Val), spin-labeled at positions 673Cys (left) and 740Cys (right). Observed and total fitted spectra are shown in gray and black, respectively. The assignment of resonances is colored accordingly**. d**, Distances between the ^19^F and spin labels at 673Cys (left) and 740Cys (right); bars show the experimental values determined by lineshape analysis (mean ± s.e.m.) of spectra shown in **c** (Methods), while dots show *r*^6^-averaged values (mean ± s.e.m.) from the intermediate structures. Errors of experimental values were determined by bootstrapping of residuals from lineshape fits. All spectra were recorded at 500 MHz and 298 K. Source data

**Extended Data Fig. 9:**
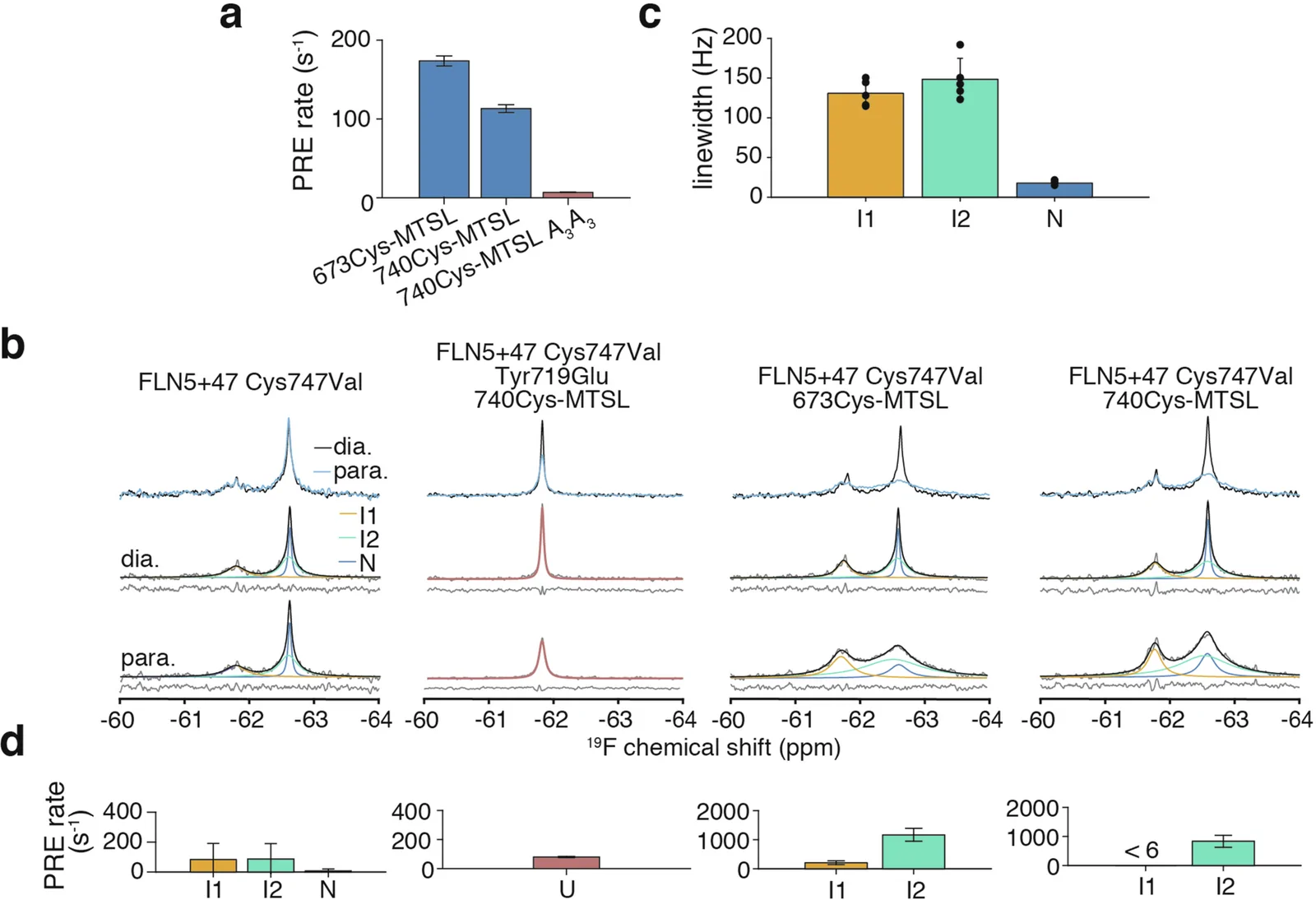
Validation of structural ensembles using ^19^F PRE measurements. **a**, PRE rates measured for isolated variants of FLN5 (pseudo wild-type Cys747Val, Methods), spectra shown in Fig. 4. Errors were determined by bootstrapping of residuals from lineshape fits (mean ± s.em.). **b**, ^19^F NMR spectra of FLN5+47 tfmF655 RNC variants under paramagnetic and diamagnetic conditions. Observed spectra shown at top, and also in grey were fitted and assigned to their conformational states (coloured). Total fitted spectra are shown in black, and residual spectrum after fitting are shown below. Spectra were recorded at 500 MHz and 298 K. **c**, Linewidths (mean ± s.d.) measured by lineshape analyses of diamagnetic spectra shown in **b**, used to calculate restraints for lineshape analyses of native state paramagnetic resonances and to determine rotational correlation times for conversion of PRE rates to distances (see Methods and Supplementary Note 5). **d**, PRE rates calculated from lineshape analyses of spectra shown in **b**. Errors were determined by bootstrapping of residuals from lineshape fits (mean ± s.e.m.).

We sought to complement the ^19^F chemical shift data by measuring long-range (10–30 Å) distances using ^19^F PRE NMR. Having identified detachment of the A–B strands as a key structural feature in I1 (Fig. 3a,b), we covalently linked paramagnetic spin labels on the adjacent B strand (Thr673Cys) and F–G loop (Asn740Cys) and measured their effect on the tfmF655 (A strand) resonance (Methods and Fig. 4a). As expected, the isolated, folded protein showed line broadening in the presence of either paramagnetic label but only marginal changes when the protein was fully unfolded (Fig. 4b and Extended Data Fig. 9a).

The native-state resonance was also broadened in paramagnetic spin-labeled FLN5+47 RNC. However, lineshape analysis of the resonances (Methods) revealed differential effects between the I1 and I2 linewidths and the two spin label sites (Fig. 4c and Extended Data Fig. 9b–d). We used the measured linewidths to calculate distances between the ^19^F and paramagnetic spin labels (Supplementary Note 5). Native-like distances between A–B strands and A-loop(FG) were evaluated for I2 (Fig. 4d), consistent with its determined structural ensemble (Fig. 3c,d). In contrast, larger distances were calculated for I1—in particular, a substantial increase (to >20 Å) for the 740Cys–MTSL spin label (measuring between A–B strands and A-loop(FG)) compared to the native state (Fig. 4d). These data agree with the structures (Fig. 3a,b), where the A strand remains detached from the C terminus across all I1 conformations, while the formation of a β-hairpin in subpopulations of I1 rationalizes the shorter ensemble-averaged distance between A and B strands.

Thus, collectively, the NMR chemical shifts (Figs. 1 and 2), crosslinking (Extended Data Fig. 8d), cryo-EM (Extended Data Fig. 8a–c) and PRE data (Fig. 4 and Extended Data Fig. 9) experimentally confirm that the MD-derived structural models of I1 and I2 (and their native-like character) are accurate.

## Folding modulated by a molecular chaperone

**Fig. 5:**
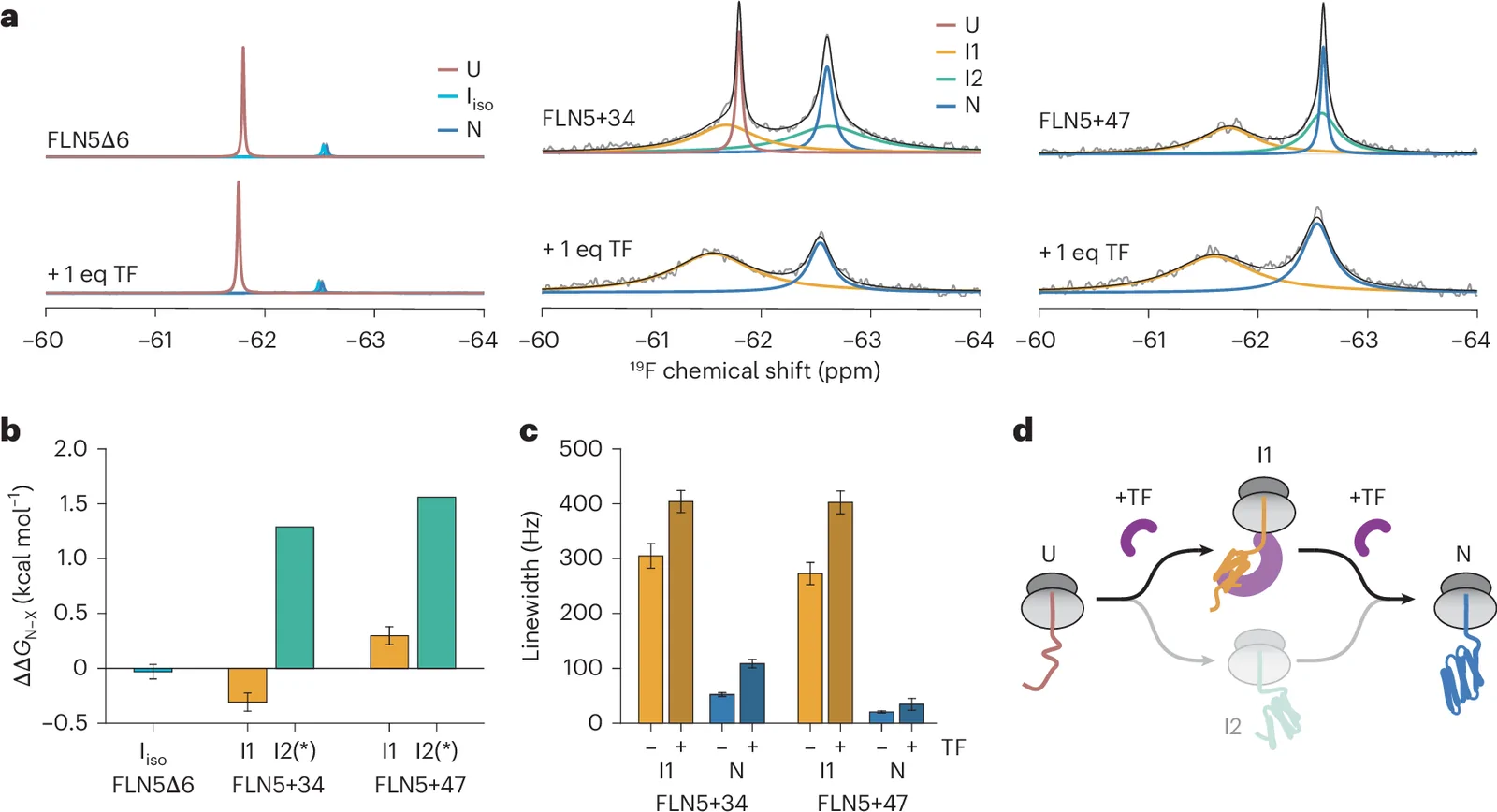
Effect of TF on coTF of FLN5. **a**, ^19^F NMR spectra of isolated FLN5∆6, FLN5+34 and FLN5+47 in the absence and presence of equimolar concentration of TF (all species were at 20 μM). Observed and total fitted spectra are shown in gray and black, respectively. The assignment of resonances is colored accordingly. All spectra were recorded at 298 K and 500 MHz. **b**, Changes in free energy differences between the intermediate and native states upon addition of TF, determined by analysis of spectra shown in **a**. The asterisk indicates the lower bound, assuming <5% population detection limit^2^. **c**, Linewidths of I1 and native states determined by lineshape analysis of spectra shown in **b. d**, Schematic depicting I1 and I2 along parallel pathways from unfolded to folded states and change in flux by TF. Unless stated otherwise, all values in the figure represent the mean ± s.e.m. propagated from NMR lineshape fits. Source data

We next examined possible interactions with the ribosome-associated molecular chaperone trigger factor (TF). In contrast to the isolated (destabilized) FLN5 variant (Fig. 5a)^11^, we observed substantial alterations in the ^19^F NMR spectra of FLN5 RNCs on addition of TF (Fig. 5a). Resonances of both I1 and the native states showed increases in their integrals and linewidths in the presence of equimolar TF, while no peaks corresponding to I2 or the unfolded states could be quantified (Fig. 5b,c). These measurements indicate selective binding of TF to I1 and the native state, increasing their populations, particularly at the shorter FLN5+34 length. This modulation by TF is most consistent with FLN5 folding through parallel (I1 or I2) rather than sequential (I1 to I2 to N) pathways (Fig. 5d), although this cannot be shown unambiguously with these experiments (Supplementary Note 6). Our data add to emerging evidence of the dependence on nascent chain length and selectivity of TF for partially folded, rather than unfolded, nascent proteins^20,54^.

## Conservation of intermediate structures across filamin domains

**Fig. 6:**
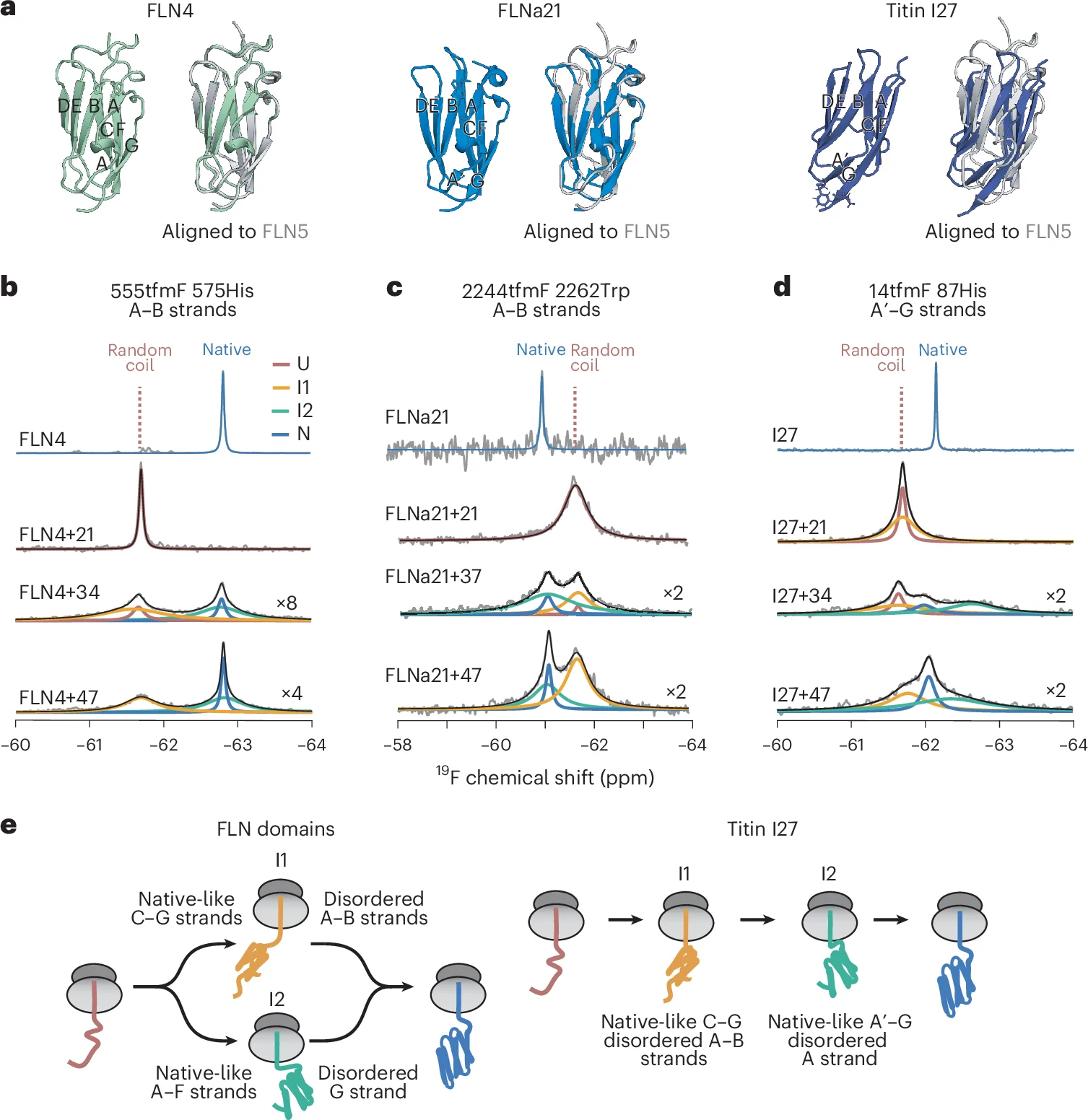
^19^F NMR of RNCs of immunoglobulin domains shows that coTF intermediates of filamins are conserved. **a**, Structures of FLN4 (AlphaFold3), FLNa21 (PDB 2W0P) and titin I27 (PDB 1TIT) and alignment with FLN5 structure (PDB 1QFH). **b**–**d**, ^19^F NMR spectra of 555tfmF 575His-labeled FLN4+*L*, 2244tfmF 2262Trp-labeled FLNa21+*L* and 14tfmF 87His-labeled I27+*L* RNCs, with linkers of *L* amino acids deriving from the subsequent domain. Top, spectra showing isolated protein spectra, with random coil value (−61.8 ppm) indicated. Observed and total fitted spectra are shown in gray and black, respectively. The assignment of resonances is colored accordingly. The magnification of spectra by a scaling factor is indicated. **e**, Summary of ^19^F NMR data of other labeling sites, shown as a schematic of coTF intermediates and pathways of the filamins (FLN4, FLN5 and FLNa21) and I27 (all NMR data shown in Extended Data Fig. 10a–c). All spectra were recorded at 500 MHz and 298 K.

Lastly, we sought to examine whether the intermediates of FLN5 are conserved across the coTF pathways of domains with a similar fold. Therefore, we studied the folding of three additional immunoglobulin-like domains (of a multidomain protein) on the ribosome (Fig. 6a): FLN4, the domain preceding FLN5 (47% identity relative to FLN5), FLNa21, the 21st domain of human filamin A (the human homolog of gelation factor; 31% identity), and I27, the 27th immunoglobulin-like domain of titin (24% identity), whose smaller size (89 versus 105 residues compared to FLN5) is accompanied by a subtly different topology. For each protein, we rationally designed and experimentally validated ^19^F-label pairs with ring currents in the natively folded state^42^, permitting us to assess interstrand contacts across each immunoglobulin-like fold.

**Extended Data Fig. 10:**
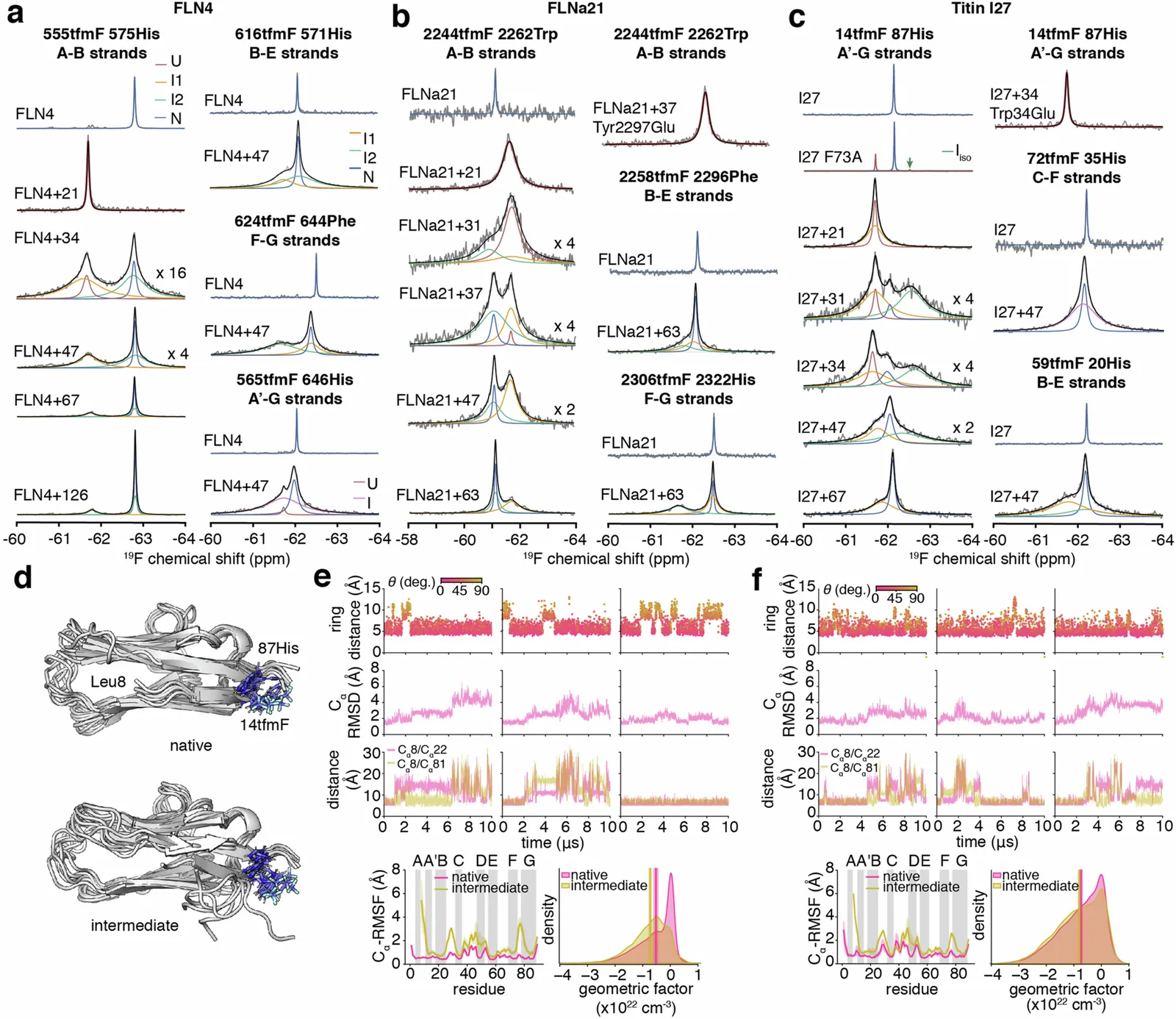
Characterisation of coTF intermediates of other immunoglobulin-like domains. **a**, ^19^F NMR spectra of isolated FLN4 and FLN4 RNC variants. **b**, ^19^F NMR spectra of isolated FLNa21 and FLNa21 RNC variants. The Tyr2297Glu mutation, analogous to Tyr719Glu in FLN5, was used to destabilise the domain. **c**, ^19^F NMR spectra of isolated titin I27 and I27 RNC variants. The Trp34Glu mutation was used to destabilise the domain^3^. Observed spectra shown in grey were fitted and assigned to their conformational states (coloured). Total fitted spectra are shown in black, and residual spectrum after fitting are shown below. Spectra were recorded at 500 MHz and 298 K. **d-f**, Structure, dynamics and aromatic ring currents of the titin I27 folding intermediate (titin I27 ΔA-strand) observed in MD simulations. **d**. Representative structures of the native and intermediate state of titin I27 ΔA. Native was defined as having C_α_-C_α_ distances of less than 10 Å for both Leu8-Glu22 and Leu8-Ala81, and intermediate was defined as having at least one of these distances above 10 Å. The sidechains of 14tfmF and 87His are shown in stick representation. **e**, From top to bottom, plot shows centre-of-mass distance between the 14tfmF CF_3_ and 87His aromatic ring coloured by the angle, θ, formed between the CF_3_-ring interaction vector and aromatic ring normal vector. Angles below and above 54.7° are considered perpendicular and in-plane CF_3_-ring interactions. Second plot shows C_α_-RMSD with respect to the starting structure across all three simulations. Third plot shows distances between the N-terminal C_α_ (residue Leu8) and C_α_ atoms of residues Glu22 and Ala81 calculated in three simulations. Bottom left shows average (mean ± s.e.m.) C_α_ -RMSF of full-length and ΔA titin I27 14tfmF 87His. The full-length simulations were analysed from previous work^42^. The location of β-strands is annotated along the sequence. Bottom right shows geometric factor (1-3cos^2^*θ*)/*r*^3^, *r* is the distance from panel in top left) quantifying the ring current effect experienced by 14tfmF due to 87His for the native and intermediate state. The distribution and average (mean ± s.e.m. from block averaging) are shown. **f**, Identical analyses as in e for titin I27 ΔA-strand 59tfmF (with wild-type His20).

To identify nascent chain conformations populated on the ribosome, we produced a library of RNCs varying in length *L* of the subsequent domain (domain + *L* RNC). FLN4 RNCs ^19^F-labeled at the A–B strands (555tfmF 575Phe) showed a sharp resonance with a random coil chemical shift at the shortest RNC length (FLN4 + 21), which reduced in population with increasing RNC length and was, thus, attributable to the unfolded state; concurrently, a sharp resonance with a native chemical shift progressively increased in signal intensity and was ascribed to the natively folded state (Fig. 6b and Extended Data Fig. 10a). Importantly, two broad resonances, maximally populated at intermediate RNC lengths (FLN4 + 34 and FLN4 + 47), were also found at each of the same chemical shifts (Fig. 6b and Extended Data Fig. 10a). Thus, identically to FLN5 (Fig. 1f), FLN4 populates two coTF intermediates on the ribosome, distinguished by the presence of A–B strand contacts. Indeed, analysis using other ^19^F-label pairs (B–E and F–G strands) indicated that the same interresidue contacts are found in FLN4 as in FLN5 coTF intermediates (Fig. 6e and Extended Data Fig. 10a). We also probed the A′–G strand using a ^19^F-label pair in FLN4 (565tfmF 646His; Extended Data Fig. 10a) and found the absence of contacts for either intermediate state; these data are consistent with the determined structures of FLN5 I1 and I2, having detached A′ and G strands respectively (Fig. 3a–d). Similar ^19^F NMR analyses of FLNa21 RNCs with analogous labeling pairs (Fig. 6c,e and Extended Data Fig. 10b) also concluded the population of two coTF intermediates with the same structural features as those of FLN5. Collectively, all the ^19^F chemical shifts measured for our filamin domain coTF intermediates show that these states contain substructures of the native topology. Given the identical contacts observed by ^19^F NMR analyses, the determined structures of FLN5 coTF intermediates appear to be remarkably conserved across the folding of filamin domains.

We previously identified two coTF intermediates of titin I27 on the ribosome^3^ and show here that their populations alter with RNC length, as expected (Fig. 6a and Extended Data Fig. 10c). However, in contrast to the filamin domains, ^19^F NMR spectra of I27 RNCs labeled across the A′–G strands (14tfmF 87His) reveal an intermediate-state resonance (I1) at the random coil chemical shift but, in contrast to the filamin domains, also at a notably more shielded, nonnative, chemical shift compared to that of the native state (I2). A weakly populated intermediate having the same chemical shift was also found in a destabilized variant of I27 (Phe73Ala) in isolation^3^ (Extended Data Fig. 10c), suggesting that its conformation was similar to the ribosome-bound I2 intermediate, whose structural features have previously been examined^55,56^. Indeed, all-atom MD simulations of the isolated I27 intermediate reconcile the observed chemical shifts and contacts of the I2 intermediate (Extended Data Fig. 10d–f and Supplementary Note 7). Overall, our experimental data suggest that the I27 coTF intermediates I1 and I2 are defined by detached A–B and A strands, respectively, from a common native-like β-sandwich core comprising β-strands C–G (Fig. 6e and Extended Data Fig. 10c). Thus, while the filamin I1 state is apparently conserved, I2 is subtly distinct to those adopted by filamin domains.

## Discussion

We developed a range of quantitative and direct ^19^F NMR analyses and used these together with MD simulations to determine atomistic structural ensembles of coTF intermediates on the ribosome. A particular strength of ^19^F NMR, crucial to obtaining direct, precise structural contacts, is its ability to resolve interconverting states^2^. We extracted short-range and long-range distances from ^19^F NMR chemical shifts (Fig. 1 and Extended Data Figs. 2 and 3) and PRE measurements (Fig. 4 and Extended Data Figs. 8 and 9), respectively, and obtained systematic structural analyses specific to each intermediate state using a psi-value-based analysis (Fig. 2 and Extended Data Fig. 4). Extensive MD simulations of the putative models of the partially folded intermediate-state conformations (Fig. 3) show striking and entire consistency with all experimental NMR, cryo-EM and biochemical datasets (Extended Data Fig. 9). Overall, our study advances the utility of ^19^F NMR in structural studies and coTF and establishes a generalizable approach to validate atomistic structures of large macromolecular complexes.

Our structural ensembles of I1 and I2 show a native-like, reduced β-sandwich core with strands C–G and A–F natively folded, respectively. Thus, while I1 and I2 are distinct in lacking their N-terminal (A–B strands) and C-terminal (G strand) structure, they share a common core comprising C–F strands. This latter feature is shared with the other immunoglobulin-like domains studied here. Given that the filamin intermediates have distinct subsets of native contacts at their domain termini, we suggest that they are likely populated along two parallel, rather than sequential, folding pathways. Although this cannot be shown unambiguously here with our equilibrium experimental data, this interpretation is supported by several other lines of indirect evidence (Supplementary Note 6)^57–63^. Moreover, G strand folding is coupled to the isomerization state of Pro742 (Extended Data Fig. 5)^11^, *trans* in I2 but *cis* in I1 and the native states (Fig. 3). The selective destabilization of I1 by the Pro742Ala substitution^2^ is also in line with an interpretation of parallel folding pathways and identifies this *cis*-proline as a conformational switch^11^. Notably, both the natively *cis*-Pro742 (ref. ^11^) and the coTF intermediate structures appear to be conserved across filamin domains (Fig. 6). In contrast, the I2 intermediate of I27 is structurally distinct, suggestive of a sequential coTF pathway (Supplementary Note 8).

The coTF intermediates appear to share structural similarities to those found transiently off the ribosome by FLN4 (I1-like, on-pathway and obligate for folding^56,64^), N-terminal fragments of FLN5 resembling I2 (also on-pathway^2,11^), C-terminal fragments of I27 (I2-like^55,56^) and those of refolding transition states of immunoglobulin-like domains^65^. On the basis of our experimental data, this observation suggests that isolated intermediate conformations are explored on the ribosome but additional conformational states not observed in isolation also become thermodynamically more accessible (Supplementary Note 9). This thermodynamic stabilization of intermediates on the ribosome persists for long durations of translation^2^, even after translation of the subsequent domain (Fig. 1d and Extended Data Fig. 10a). The prolonged lifetimes of intermediates, in contrast to highly transient populations off the ribosome, may provide an opportunity for chaperones^19–21^ (Fig. 5), cofactors^64^, enzymes and quality control machinery^66^ to survey, engage and modify^67^ specific nascent chain conformations or for recruitment of other nascent proteins during cotranslational assembly^31,32^. Intriguingly, filamin function is highly regulated by serine phosphorylation at consensus sequences in humans^68^. Strand detachment and a *trans*-proline in the I2 intermediate exposes a natively buried phosphorylation site, potentially implicating the formation of stable coTF intermediates to cotranslational modifications^69,70^.

Future detailed characterizations of nascent chain structure will permit mechanistic insights into folding pathways of other protein folds, misfolding^29,30,71,72^ and other cotranslational events.

## Methods

### Sample preparation

DNA constructs of FLN5, FLN4, FLNa21 and titin I27 were previously described^2,3,39,42^. Mutations and amber stop codons were introduced using standard site-directed mutagenesis procedures and constructs were verified by DNA sequencing. For PRE NMR experiments, the additional substitution Cys747Val was introduced to yield a cysteine-free background for site-specific spin labeling, as previously described^3^. Isolated proteins were expressed as His-tagged proteins (FLNa21 as GST-fused protein^42^), isotopically labeled in *Escherichia* *coli* BL21 DE3-Gold cells (using amber suppression for incorporation of tfmF) and purified as previously described^2,39,42^. RNC constructs comprised a His-tag for purification and an arrest-enhanced variant of the SecM stalling sequence, FSTPVWIWWWPRIRGPP, as previously described^2^. RNCs were expressed, isotopically labeled and purified as previously described^2^. For intermolecular crosslinking experiments, RNCs were expressed in CRISPR-modified *E*. *coli* BL21 strains with cysteine substitutions in uL24, as previously described^3^. MTSL spin labels for PRE NMR were introduced into purified samples, as previously described^3^. Sample purity and integrity were assessed using SDS–PAGE and western blot analyses with an anti-His_5_ horseradish-peroxide-conjugated antibody (Invitrogen, 1:5,000 dilution), as previously described^73^. Unlabeled TF was purified as previously described^74^.

### NMR spectroscopy

NMR samples were prepared in Tico buffer (10 mM HEPES, 30 mM NH_4_Cl, 12 mM MgCl_2_ and 1 mM EDTA), supplemented with 10% (v/v) D_2_O and 0.001% (w/v) DSS as a reference. NMR data were recorded using a 500-MHz (^19^F and ^1^H,^15^N NMR) or 800-MHz (^1^H,^15^N NMR) Bruker Avance III spectrometer, both equipped with a TCI cryoprobe and Topspin 3.5pl2, at 298 K, unless stated otherwise. Data were processed using NMRPipe (version 11.7)^75^, CCPN (version 2.4), MATLAB (R2017a, Mathworks) and Julia (version 1.5)^76^.

To obtain quantitative insights into the structure (short-range contacts) and populations of intermediates on the ribosome, one-dimensional (1D) ^19^F pulse-acquire experiments were recorded using a 350-ms acquisition time and 1.5-s recycle delay. Multiple short experiments were recorded in succession to monitor changes in RNC sample integrity and data indicating nascent chain release or sample degradation through changes in linewidths, signal intensities or chemical shifts were not used to produce the final summed spectrum, as previously described^2,3^. Processed spectra were baseline-corrected and peaks were fitted to Lorentzian lineshapes, with errors of the linewidths and integrals (populations) estimated using bootstrapping (200 iterations, calculating the s.e.m.), as previously described^2^. The time-domain ^19^F NMR spectra were multiplied with an exponential window function with a line broadening factor of 5 (isolated protein) or 10 Hz (RNC), unless stated otherwise, before Fourier transformation. Integrals were used to calculate folding free energies using ∆*G* = −*RT*ln(*K*_eq_). Two-dimensional (2D) ^1^H,^15^N band-selective optimized flip angle short transient definition (SOFAST) heteronuclear multiple-quantum correlation (HMQC) experiments^77^ were recorded using an acquisition time of 50 ms in the direct dimension and interscan delays of 100 ms. Cosine-squared window functions were used to process the spectra.

Long-range distance measurements were obtained using ^19^F PRE experiments, where PRE rates were measured using 1D pulse-acquire experiments with samples in the paramagnetic and diamagnetic state, the latter by addition of 2.5 mM (RNC) or 100× molar excess (isolated protein) sodium ascorbate. For PRE measurements of RNCs, spin-labeled samples were prepared and split into two halves; one half was immediately recorded by NMR at 298 K to produce the paramagnetic spectrum, while the other half was incubated at 277 K. Once the paramagnetic spectrum was acquired (typically 16–24 h), the latter half sample was reduced in sodium ascorbate and the diamagnetic spectrum acquired at 298 K (typically also 16–24 h). To improve the signal-to-noise ratio, 5–6 separate RNC samples were prepared and the datasets summed together (using only data corresponding to intact samples) to produce the final spectra. The spectra were fitted to Lorentzian lineshapes, as previously described^2^, which provide accurate measurements of transverse relaxation rates (*R*_2_) and identical to those obtained from direct ^19^F *R*_2_ measurements^3^. Line broadening resulting from the PRE effect resulted in highly overlapped I2 and native-state resonances, which were deconvolved by restraining the lineshape fit of the native state using the expected PRE rate bounds (Supplementary Note 5).

^19^F CEST measurements were performed and analyzed as previously described^2^. These experiments aimed to quantify the exchange rates between different conformational states. Experiments were recorded with an acquisition time of 200 ms and a recycle delay of 30 ms, with a weak B1 field of 15 Hz applied for a saturation time of 800 ms at saturation frequencies of −41, −61.8, −62.2 and −63.0 ppm.

### AlphaFold structure predictions

AlphaFold3 (https://alphafoldserver.com)^46^ was used to predict structures of FLN5 bihistidine mutants in complex with a metal ion, using Co(II) to replace Ni(II) that is otherwise unavailable. AlphaFold3 was also used to predict the structure of flexible regions of FLN4 that are absent in its crystal structure (PDB 1WLH). Default settings were applied.

### Crosslinking experiments

To probe RNC interactions site-specifically, crosslinks were introduced into FLN5+47 RNC using a similar approach as described for PRE spin labeling^3^. Samples were reduced overnight at 277 K in Tico supplemented with 2 mM TCEP. TCEP was then removed by buffer exchange into labeling buffer (50 mM HEPES, 12 mM MgCl_2_, 20 mM NH_4_Cl and 1 mM EDTA, pH 8.0). The RNC sample was then incubated with variable concentrations of the crosslinker 1,8-bismaleimido-diethyleneglycol (BM(PEG)_2_) at 298 K and the reaction was quenched with DTT. Crosslinking was assessed by a mass increase of ~16 kDa (that is, the mass of uL24) by western blot.

### Simulating coTF during real-time synthesis

To assess whether folding is expected to occur during real-time translation (compared to equilibrium measurements performed by ^19^F NMR), we considered a kinetic model. We simulated coTF during real-time synthesis for FLN5 using a system of ordinary differential equations (ODEs) representing a four-state model with unfolded, I1, I2 and native states. Folding was modeled as a function of nascent chain length during vectorial synthesis at fixed elongation rates of 5 and 20 aa per second (approximate eukaryotic and prokaryotic translation rates, respectively). Simulations were initiated at FLN5+31, corresponding to full emergence of the FLN5 domain from the ribosome^39^ and 100% unfolded state. Calculations were then performed until the maximum chain length the polypeptide achieves on the ribosome before release of full-length gelation factor (FLN5+110)^39^. We set a maximum folding rate, *k*_f_, of 0.8 s^−1^, corresponding to the apparent folding rate previously measured for isolated FLN5 experimentally^11^. As the ribosome has been shown to reduce folding rates at short NC linker lengths, we used a decrease in folding rate by two orders of magnitude (0.008 s^−1^) on the basis of experimental measurements^9^. We then linearly interpolated the folding rate between these two values as a function of chain length and synthesis time. Prior experimental studies have indicated that folding rates, in fact, accelerate more rapidly than expected by linear interpolation^34^; therefore, this represents a lower bound (that is, slowest folding rate feasible given experimental information). We assumed all forward rates to be equal to the length-dependent value of *k*_f_. This also represents a conservative choice of a lower bound for individual rates as apparent, measured folding rates are generally faster than conversion rates to and from intermediates. Experimentally determined equilibrium populations for unfolded, I1, I2 and native states at FLN5+31, FLN5+34, FLN5+47, FLN5+67 and FLN5+110 (refs. ^2,3^ and this study) were used to calculate length-dependent free energy differences (*ΔG*) between states and interpolated linearly between these discrete chain length measurements. Reverse (unfolding) rates (*k*_u_) were obtained from the NC length-specific values of *k*_f_ and *ΔG* using *k*_u_ = *k*_f_·exp(*ΔG*/*RT*). The ODE system was numerically integrated over time using solve_ivp (SciPy, version 1.3.1) with default settings and 1,000 evenly spaced time steps. Simulated folding populations were plotted as a function of chain length and compared to experimental equilibrium measurements. The following system of ODEs was used to model NC populations during real-time biosynthesis, where U and N denote the unfolded and native states:

$$\frac{\mathrm{dU}}{{\rm{d}}t}=-{k}_{\mathrm{UI}1}\bullet \left[{\rm{U}}\right]+{k}_{{\rm{I}}1{\rm{U}}}\bullet \left[{\rm{I}}1\right]-{k}_{\mathrm{UI}2}\bullet \left[{\rm{U}}\right]+{k}_{{\rm{I}}2{\rm{U}}}\bullet \left[{\rm{I}}2\right]-{k}_{\mathrm{UN}}\bullet \left[{\rm{U}}\right]+{k}_{\mathrm{NU}}\bullet \left[{\rm{N}}\right]$$

$$\frac{\mathrm{dI}1}{{\rm{d}}t}={k}_{\mathrm{UI}1}\bullet \left[{\rm{U}}\right]-{k}_{{\rm{I}}1{\rm{U}}}\bullet \left[{\rm{I}}1\right]-{k}_{{\rm{I}}1{\rm{I}}2}\bullet \left[{\rm{I}}1\right]+{k}_{{\rm{I}}2{\rm{I}}1}\bullet \left[{\rm{I}}2\right]-{k}_{{\rm{I}}1{\rm{N}}}\bullet \left[{\rm{I}}1\right]+{k}_{\mathrm{NI}1}\bullet \left[{\rm{N}}\right]$$

$$\frac{\mathrm{dI}2}{{\rm{d}}t}={k}_{\mathrm{UI}2}\bullet \left[{\rm{U}}\right]-{k}_{{\rm{I}}2{\rm{U}}}\bullet \left[{\rm{I}}2\right]+{k}_{{\rm{I}}1{\rm{I}}2}\bullet \left[{\rm{I}}1\right]-{k}_{{\rm{I}}2{\rm{I}}1}\bullet \left[{\rm{I}}2\right]-{k}_{{\rm{I}}2{\rm{N}}}\bullet \left[{\rm{I}}2\right]+{k}_{\mathrm{NI}2}\bullet \left[{\rm{N}}\right]$$

$$\frac{\mathrm{dN}}{{\rm{d}}t}={k}_{\mathrm{UN}}\bullet \left[{\rm{U}}\right]-{k}_{\mathrm{NU}}\bullet \left[{\rm{N}}\right]+{k}_{{\rm{I}}1{\rm{N}}}\bullet \left[{\rm{I}}1\right]-{k}_{\mathrm{NI}1}\bullet \left[{\rm{N}}\right]-{k}_{\mathrm{NI}2}\bullet \left[{\rm{N}}\right]+{k}_{{\rm{I}}2{\rm{N}}}\bullet \left[{\rm{I}}2\right]$$

To model different scenarios of intermediates being populated along parallel pathways or sequentially along a common pathway, we set the rate constants of ‘forbidden’ transitions to 0 s^−1^.

### Proline free energy calculations

To quantitatively assess the regulatory role of P742 isomerization on the stability and folding of FLN5, we used well-tempered metadynamics^78^ (WT-METAD) simulations and calculated the free energy landscapes capturing the isomerization state of FLN5 proline P742. All-atom simulations were run using the DES-Amber force field and TIP4P-D water model^79,80^. The calculations were prepared and performed with GROMACS (version 2020)^81^ and PLUMED (version 2.6)^82,83^. Three different models were used to investigate the effect of local structure on P742 isomerization energetics: an unfolded peptide with P742 (underlined) in the middle of the sequence corresponding to FLN5 residues N736-I748 (Ac-NPIKNMPIDVKCI-NMe), a C-terminally truncated FLN5 construct, FLN5 Δ6, populating a folded and disordered C terminus at equilibrium^11^, and full-length, folded FLN5 (PDB 1QFH, residues K646–G750)^84^. Simulations of the unfolded peptide were initiated from a linear chain built in PyMOL 2.3 (Schrödinger). All systems were initially centered in a dodecahedral simulation box at least 1.2 nm away from the box edge, solvated and neutralized with MgCl_2_. Energy minimization of the solvated systems was performed using the steepest descent algorithm using a tolerance of <1,000 kJ mol^−1^ nm^−1^. Equilibration was then performed for 1 ns in the NVT and 1 ns in the NPT ensemble, where the temperature (298 K) was maintained using velocity rescaling^85^ with a coupling constant of 0.1 ps and the pressure (1 bar) was regulated with the Berendsen barostat^86^ a compressibility of 4.5 × 10^−5^ bar^−1^ and coupling constant of 2 ps. During equilibration all protein heavy atoms were position-restrained using a force constant of 1,000 kJ mol^−1^ nm^−2^. Position restraints were then removed and pressure coupling switched to the Parrinello–Rahman method^87^ with the same compressibility and coupling constant. A time step of 2 fs was used together with the leapfrog integrator and the LINCS algorithm^88^ to constrain all bonds involving hydrogen atoms. Production simulations with WT-METAD were run in triplicates from different initial velocities for 1 μs each. Van der Waals interactions were computed up to a cutoff distance of 0.9 nm and short-range electrostatics were computed up to 1 nm. Long-range electrostatics were calculated using the particle mesh Ewald method^89^ with cubic interpolation and a grid spacing of 0.125 nm.

As collective variables for WT-METAD, we used two dihedral angles, ζ (Cα_*i*__−1_–O_*i*__−1_–Cδ_I_–Cα_*i*_) and ψ (N_*i*_–Cα_*i*_–C_*i*_–N_*i*+1_), where *i* corresponds to P742. ζ describes the isomerization state (~0 for *cis* and ~±*π* for *trans* in radians) and ψ describes the the C-terminal amide orientation. Previous work has shown this combination to be efficient for simulating proline isomerization with WT-METAD^90^. Gaussians were deposited every 1 ps with a width of 0.07 rad for each dihedral angle. The Gaussian height and bias factor were set to 0.8 kJ mol^−1^ and 10, respectively. We used the method described by Tiwary et al. to reweight all snapshots to correct for the WT-METAD bias potential (saved every 5 ps)^91^, discarding the first 200 ns (20%) of each trajectory. All ensemble properties including *cis*/*trans* populations were then calculated using these weights, with the mean and s.e.m. calculated from the three independent WT-METAD simulations initiated with different starting velocities. For full-length FLN5 and FLN5 Δ6, we used a collective variable to quantify the fraction of native contacts (Q) formed by the two downstream residues I743 and D744 to correlate C-terminal structure with P742 isomerization. SMOG2 (ref. ^92^) was used to obtain a list of native heavy atom contacts and Q was calculated using the following switching function^93^:

$${\boldsymbol{Q}}\left({\boldsymbol{X}}\,\right)=\frac{{\bf{1}}}{{\boldsymbol{N}}}\mathop{\sum }\limits_{{\boldsymbol{ij}}}\frac{{\bf{1}}}{{\bf{1}}+{{\boldsymbol{e}}}^{\left({\boldsymbol{\beta }}\left({{\boldsymbol{r}}}_{{\boldsymbol{i}},\,{\boldsymbol{j}}}-{\boldsymbol{\lambda }}{{{\boldsymbol{r}}}^{{\bf{0}}}}_{{\boldsymbol{i}},\,{\boldsymbol{j}}}\right)\right)}}$$

The distance between two heavy atoms is defined as *r*_*i*,*j*_, whereas *r*^0^_*i*,*j*_ corresponds to the reference distance. *β* and λ were set to 50 nm^−1^ and 1.8, respectively. WT-METAD simulations of full-length FLN5 were restrained to have a C-terminal *Q* > 0.8 using a lower wall restraint in PLUMED (force constant = 10,000 kJ mol^−1^) to maintain the native structure of FLN5 during the simulations.

### All-atom simulations of protein folding

With the approach described here, we aimed to sample starting structures and models of coTF intermediates. We used rMD simulations^48,51,94,95^ to sample folding transition paths starting from unfolded conformations. For isolated FLN5, we used SMOG2 (ref. ^92^) to generate a simple structure-based model without any contacts (that is, no nonbonded attractive potentials) and unfold FLN5. A total of 50 random unfolded conformations were then picked for rMD simulations. Using the DES-Amber force field and TIP4P-D water model^79,80,96^, these structures were then energy-minimized and equilibrated using an equivalent protocol as detailed above at 350 K. For the FLN5+47 RNC, we performed thermal unfolding simulations starting from 50 different conformations of folded FLN5+47 on the ribosome obtained in previous work^23^. The RNC was simulated with using the DES-Amber force field and TIP4P-D water model^79,80,96^, as we showed that this force field results in good agreement of folded FLN5 simulations with NMR data^23^. For thermal unfolding and subsequent relaxation runs, we used hydrogen mass repartitioning^97^ and a 4-fs timestep with LINCS. GROMACS 2020 and PLUMED 2.6 were used for these simulations. Temperature and pressure coupling settings were identical to the production runs used for the proline free energy calculations described above. All ribosome heavy atoms (and C-terminal nascent chain residue) were position-restrained at all times using a force constant of 1,000 kJ mol^−1^ nm^−1^. Unfolding was performed at 400 K and 1 bar for 25 ns in the presence of lower wall restraints on the radius gyration calculated from residues 646–750 Cα atoms at 3 nm with a force constant of 10,000 kJ mol^−1^. The total fraction of native contacts, as defined in the previous section, was restrained to be below 0.02 using a force constant of 10,000 kJ mol^−1^. To ensure all starting structures had a *trans* peptide bond for P742 (P742 is in the *cis* conformation in the native structure), we additionally restrained the absolute value of the ζ dihedral angle to be above 2.62 rad (~150°). These unfolded structures were then relaxed at 350 K and 1 bar for 1 ns in the absence of wall restraints (but with ribosome position restraints).

Enhanced sampling is achieved with the rMD method (adiabatic-bias MD in PLUMED). We used GROMACS 2020 and PLUMED 2.6 for rMD simulations. In this approach, simulations proceed in the absence of a biasing potential when spontaneous progression toward a predefined target state (that is, the folded state of FLN5) occurs. However, a soft biasing potential is applied when the system moves in the opposite direction^48,94^:

$${F}_{\mathrm{rMD}}=\left\{\begin{array}{l}\frac{\kappa }{2}{\left(\,\rho \left(t\right)-{\rho }_{m}\left(t\right)\right)}^{2},\,\rho \left(t\right) > {\rho }_{m}\left(t\right)\\ 0,\,\rho \left(t\right)\le {\rho }_{m}\left(t\right)\end{array}\right\}$$

$$\rho \left(t\right)={\left(\mathrm{CV}\left(t\right)-{\mathrm{CV}}_{\mathrm{target}}\right)}^{2}$$

$${\rho }_{m}\left(t\right)=\mathop{\min }\limits_{0\to t}\left(\mathrm{CV}\right)$$

The force constant, *κ*, controls the magnitude of the bias potential, CV(*t*) is the instantaneous value of a defined collective variable at time *t* and CV_target_ is its value in the target (folded) state. Therefore, rMD effectively promotes transitions from an initial (unfolded) state to the final (native state) using a reaction coordinate. In our rMD simulations, we set *κ* and CV_target_ to 100 kJ mol^−1^ and 0, respectively. As a collective variable, we used the difference contact map between a given conformation and the native state^48,94^, defined by heavy atom native contacts, *j*, within a 0.6-nm distance cutoff obtained the shadow algorithm implemented in SMOG2 (ref. ^92^):

$$\mathrm{CV}=\sqrt{{\sum }_{j=1}^{n}{\left({C}_{j}\left(X\,\right)-{C}_{j}\left({X}_{\mathrm{native}}\right)\right)}^{2}}$$

$${C}_{j}\left(X\,\right)=\frac{1-{\left(\frac{{r}_{j}}{{r}_{0}}\right)}^{6}}{1-{\left(\frac{{r}_{j}}{{r}_{0}}\right)}^{10}}$$

Here, *r*_*j*_ is the native contact distance for atom pair *j* and *r*_0_ was set to 0.5 nm. For FLN5, this approach includes 1,009 heavy atom contacts calculated using the energy-minimized crystal structure of FLN5. For all 50 starting structures, we ran 36 independent rMD simulations starting from randomly assigned initial velocities for a time of 5 ns, totaling 1,800 trajectories and 9 µs of biased sampling. Simulations were run in the NPT ensemble at 350 K, using the same temperature and pressure coupling parameters as described above. The integration time step was 2 fs. For the RNC, position restraints on the heavy atoms of the ribosome and C-terminal NC residue at the peptidyl transferase center were kept at 1,000 kJ mol^−1^ nm^−1^. We then calculated the root-mean-square deviation (r.m.s.d.) over Cα atoms of residues 646–750 for all trajectories with respect to the energy-minimized crystal structure of FLN5. Trajectories reaching an r.m.s.d. of less than 0.2 nm were defined as successful folding transition paths, resulting in 321 and 226 successful folding transitions for isolated FLN5 and FLN5+47 RNC, respectively. These trajectories were analyzed further and we calculated the total fraction of native contacts and native contacts between specific structural elements of the protein to project the transition path landscape onto these variables to investigate the folding pathway and intermediate states.

### Unbiased all-atom simulations of coTF intermediates

Models of coTF intermediates were subjected to unbiased MD simulations to assess the stability of their structures and their dynamic ensembles. To select starting structures of the I1 coTF intermediate, we considered all potential structures belonging to the local free energy minimum observed in rMD simulations between a fraction of a global *Q* (0.55 < Q < 0.68; Extended Data Fig. 6b–f). As this intermediate free energy minimum corresponds to structures with a folded G strand (Extended Data Fig. 6f) and our proline free energy calculations show that P742 is in the *cis* configuration in this case (Extended Data Fig. 5), we only selected structures with P742 in *cis*. To ensure the selection of physically reasonable structures, we sorted the structures by ascending distance r.m.s.d. (relative to the energy-minimized native-state crystal structure) of all heavy atoms belonging to residues F691–G750. These residues correspond to the natively folded region of this intermediate state predicted by rMD (Extended Data Fig. 6c). The top 100 structures were then clustered using the GROMOS algorithm^98^ implemented in gmx cluster, using all-atom r.m.s.d. of the entire nascent chain without alignment and a cutoff of 0.9 nm to select structures with different orientations on the ribosome. The top five cluster centroids (Extended Data Fig. 6g) were then used for unbiased MD simulations.

As rMD simulations only sampled I1 (Extended Data Fig. 6) and because of the structural similarity between the I2 coTF intermediate and an intermediate previously characterized for isolated FLN5 Δ6 (refs. ^3,11,41^), we randomly selected five structures from the lowest free energy cluster of the NMR-derived structural ensemble of isolated FLN5 Δ6 (ref. ^11^). We then built the C-terminal residues of FLN5 in an extended conformation and joined these models with the NC linker for FLN5+47 used in previous work^23^. Unbiased MD simulations were then initiated from these structural models.

For all input structures of I1 and I2, we placed these complexes in the center of a dodecahedron box and added solvent atoms and MgCl_2_ to neutralize the charge of the system. The box size was determined by gmx editconf using minimum distances of 3 and 2 nm from the box edge for I1 and I2, respectively. This resulted in system sizes of 1.2–1.7 million atoms. After energy minimization, we first gently heated the simulation boxes from 0 to 298 K over a 500 ps using a time step of 0.5 fs and LINCS constraints for all bonds involving hydrogen atoms. The systems were then equilibrated at 298 K in the NVT ensemble (velocity rescaling thermostat with a coupling constant of 0.1 ps) for 500 ps using a 2-fs time step, followed by a 1-ns equilibration step in the NPT ensemble at 1 bar (Berendsen barostat with a time constant of 2 ps). During the equilibration protocol, all RNA and protein heavy atoms were position-restrained (1,000 kJ mol^−1^ nm^−2^). These restraints were then removed for all NC atoms (except the C-terminal residue at the peptidyl transferase center) for a further 1-ns equilibration simulation (NPT, Parrinello–Rahman barostat, 2-ps coupling constant) and production simulations. We performed three unbiased simulations starting from different initial velocities for each starting structure lasting 1 μs, resulting in a total of 15 μs for each coTF intermediate state.

Root-mean-square fluctuation (r.m.s.f.) and SASA analyses were performed with gmx rmsf and sasa^81^, respectively. Alignment before r.m.s.f. calculation was performed using Cα atoms of residues in the folded region of the intermediate state, F691–G750 (C strand to C terminus) and K646–L733 (N terminus to F strand) for I1 and I2, respectively. The fraction of native contacts (*Q*) was calculated using PLUMED as described above. The radius of gyration was computed using all Cα atoms of the FLN5 domain (residues K646–G750) with PLUMED to compare the compactness of the coTF intermediates to the native state. Contact maps, ribosome interactions, distances and secondary-structure content were calculated using the MDTraj^99^ and MDAnalysis^100^ Python libraries. The effective rotational correlation times of the coTF intermediates were calculated as previously described^23^. The rotation of the domain was defined using backbone heavy atoms belonging to the same regions as defined for the r.m.s.f. analysis of I1 and I2. Structural analyses were compared to the native-state ensemble of FLN5 previously obtained^23^ with the DES-Amber force field. All ensemble properties were averaged over the entire 15-μs ensemble and errors are reported as the s.e.m. obtained from the 15 independent 1-μs trajectories.

The structural ensembles were clustered using for the purpose of structural visualization using gmx cluster^81^ and the GROMOS algorithm^98^. Alignment and r.m.s.d. calculations for clustering were performed using the same residue ranges as for the r.m.s.f. analysis and all backbone heavy atoms. Similarity cutoffs of 0.2 and 0.1 nm were chosen for I1 and I2, respectively, because of these values resulting in similar cluster populations (Supplementary Table 4). Structures from the most populated cluster were then used for visualization in the main text figure (Fig. 3a–d).

### Simulations of fluorinated protein variants in isolation

Additional MD simulations of fluorinated protein variants were used to help reconcile experimental observations of ^19^F NMR chemical shifts and the origins of (de)shielding effects in different conformational states. We used the CHARMM36m (ref. ^101^) and ff15ipq (refs. ^102,103^) force fields to simulate the following isolated FLN5 variants labeled with tfmF: 655tfmF, 691tfmF, 705tfmF, 715tfmF, 719tfmF, 727tfmF, 732tfmF and 740tfmF. We used an identical simulation protocol as described previously^42^. Production simulations were run for 1 μs, saving coordinates for analysis every 0.1 ns. Titin I27 variants lacking the A strand (residues 1–7 deleted) were simulated with the ff15ipq (ref. ^102^) force field for 10 μs in triplicate to sufficiently sample the folding intermediate. GROMACS tools gmx sasa, rmsd and rmsf were used for general analyses of the CF_3_ group (SASA) and protein structure and dynamics (rmsd and rmsf). Hydrogen bonds between fluorine (acceptor) and donor atoms were analyzed with gmx hbond. To estimate hydrogen-bond lifetimes, we calculated the area under the averaged autocorrelation function from all hydrogen-bond existence functions^104,105^. Distances were calculated with in-house Python scripts using MDAnalysis^100^ and CF_3_–aromatic interactions were analyzed with an open-source Python script (https://github.com/julian-streit/RingCurrents19F).

Simulations isolated FLN5 fragments mimicking coTF intermediates I1 and I2 were performed using the ff15ipq force field^102^ and minimal constructs comprising the folded core of each intermediate. We used residues Ac-A683–G750-NMe and S645–D744 for I1 and I2, respectively. Three different starting structures were taken from the same rMD-generated models as used for the unbiased RNC simulations (I1) and FLN5 Δ6 structural ensemble^11^ (I2). Independent simulations were performed using the same protocol as for full-length fluorinated proteins from these starting structures with random initial velocities to obtain three replicates where the I1/I2 topology was maintained during the 1-μs simulations. This was assessed by having at least one ^19^F NMR residue pair with a native-like distance per β-strand pair (within 0.1 nm of the average distance calculated for the FLN5+47 native state^23^. The native β-strand pairs measured by ^19^F NMR for I1 were CF/FG and AB/BE/DE/CF for I2.

### ^19^F PRE distance calculations

^19^F NMR PRE rates *Γ*_*2*_ were converted to distances, *r*, using the Solomon–Bloembergen equation^106^, where *ω*_F_ and *τ*_c_ are the Larmor frequency of fluorine-19 and rotational correlation time, respectively:

$$r={\left(\frac{K}{{\Gamma }_{2}}\times \left(4{\tau }_{{\rm{c}}}+\frac{3{\tau }_{{\rm{c}}}}{1+{\omega }_{{\rm{F}}}^{2}{{\tau }_{{\rm{c}}}}^{2}}\right)\right)}^{1/6}$$

*K* (1.092 × 10^−44^ m^6^ s^−2^) is defined as a product of physical constants including the vacuum permeability (*μ*_*0*_), gyromagnetic ratio of fluorine-19 (*γ*_F_) electron *g* factor, Bohr magneton (*μ*_B_) and the spin number of fluorine-19 (*S*).

$$K=\frac{1}{15}{\left(\frac{{\mu }_{0}}{4\pi }\right)}^{2}{\gamma }_{{\rm{F}}}^{2}{g}^{2}{\mu }_{{\rm{B}}}^{2}S\left(S+1\right)$$

Values of *τ*_c_ were determined in previous studies^40^ and those for RNCs were determined from the linewidths using an empirical relationship recently determined^3^. Therefore, to extrapolate the expected PRE of native FLN5 on the ribosome, we used the measured PRE distance in isolation and RNC-specific *τ*_c_:

$${\Gamma }_{2}=\frac{K}{{r}^{6}}\times \left(4{\tau }_{{\rm{c}}}+\frac{3{\tau }_{{\rm{c}}}}{1+\,{\omega }_{{\rm{F}}}^{2}{{\tau }_{{\rm{c}}}}^{2}}\right)$$

### Cryo-EM fitting

To orthogonally assess whether our structural models are compatible with existing cryo-EM data, we fitted the ribosome model used in the MD simulations to each cryo-EM map representing the four FLN5+47 dataset classes (AP1, AP2, AP5 and P5)^53^. The obtained rotation–translation matrix was applied in Python using the MDAnalysis library to fit the NC structure from each frame of the MD trajectory (native, I2 and I1 states) into the NC density. Next, we used scripts from cryoENsemble^52^ to generate synthetic cryo-EM maps for each MD frame at 8-Å resolution. The correlation coefficient between each synthetic map and the corresponding experimental cryo-EM map was then calculated following the definition of map correlation by ChimeraX^107^.

As an alternative approach, we also used the cryo-EM maps to reweight the combined trajectories corresponding to native, I2 and I1 states using the cryoENsemble iterative strategy^52^. Before reweighting, we clustered each trajectory using the GROMOS algorithm in gmx cluster with a 1-nm cutoff. The obtained clustered trajectory, comprising 2,294 conformations (1,091 from I1, 706 from I2 and 497 from native state), was used in four cryoENsemble reweighting runs in each of the cryo-EM maps of FLN5+47, following the previously described protocol^52^.

### Reporting summary

Further information on research design is available in the Nature Portfolio Reporting Summary linked to this article.

## Online content

Any methods, additional references, Nature Portfolio reporting summaries, source data, extended data, supplementary information, acknowledgements, peer review information; details of author contributions and competing interests; and statements of data and code availability are available at https://doi.org/10.1038/s41594-026-01814-7.

## Supplementary information

Supplementary Information Supplementary Notes 1–9 and Tables 1–5.

Reporting Summary

Peer Review file

Supplementary Video 1 Exemplar rMD trajectory of FLN5+47 RNC showing folding sequence through I1 conformation with disordered A–B strands.

Supplementary Video 2 Exemplar rMD trajectory of FLN5+47 RNC showing folding sequence through I1 conformation with a detached A–B β-hairpin.

Supplementary Video 3 Exemplar unbiased MD trajectory (1 µs) of the FLN5+47 RNC I1 state with a detached A–B β-hairpin.

Supplementary Video 4 Exemplar unbiased MD trajectory (1 µs) of the FLN5+47 RNC I2 state with a detached G strand.

## Source data

Source Data Figs. 2–5 and Extended Data Figs. 1–4 and 6–8 Statistical source data.

## Data Availability

The structural ensembles of FLN5+47 I1 and I2 are available from Zenodo (https://doi.org/10.5281/zenodo.16601045)^108^. The NMR data are available from Zenodo (https://doi.org/10.5281/zenodo.19210765)^109^. This study made use of public datasets deposited to the Protein Data Bank under accession codes 1QFH (FLN5), 1TIT (titin I27) and 2W0P (FLNa21). Source data are provided with this paper.

## Code availability

Pulse sequences used for the acquisition of NMR data and Matlab and Julia codes used to fit the ^19^F NMR data are available from Zenodo (https://doi.org/10.5281/zenodo.15169089)^110^. Python scripts used to predict aromatic ring interactions for fluorinated protein variants are available from GitHub (https://github.com/julian-streit/RingCurrents19F) and Zenodo (https://doi.org/10.5281/zenodo.15173797)^111^.

## References

[1] Waudby, C. A., Dobson, C. M. & Christodoulou, J. Nature and regulation of protein folding on the ribosome. *Trends Biochem. Sci* **44**, 914–926 (2019). 10.1016/j.tibs.2019.06.008 PMC7471843 31301980

[2] Chan, S. H. S. et al. The ribosome stabilizes partially folded intermediates of a nascent multi-domain protein. *Nat. Chem.* **14**, 1165–1173 (2022). 10.1038/s41557-022-01004-0 PMC7613651 35927328

[3] Streit, J. O. et al. The ribosome lowers the entropic penalty of protein folding. *Nature* **633**, 232–239 (2024). 10.1038/s41586-024-07784-4 PMC11374706 39112704

[4] Samelson, A. J., Jensen, M. K., Soto, R. A., Cate, J. H. & Marqusee, S. Quantitative determination of ribosome nascent chain stability. *Proc. Natl Acad. Sci. USA* **113**, 13402–13407 (2016). 10.1073/pnas.1610272113 PMC5127326 27821780

[5] Cassaignau, A. M. E. et al. Interactions between nascent proteins and the ribosome surface inhibit co-translational folding. *Nat. Chem.* **13**, 1214–1220 (2021). 10.1038/s41557-021-00796-x PMC8627912 34650236

[6] Jensen, M. K., Samelson, A. J., Steward, A., Clarke, J. & Marqusee, S. The folding and unfolding behavior of ribonuclease H on the ribosome. *J. Biol. Chem.* **295**, 11410–11417 (2020). 10.1074/jbc.RA120.013909 PMC7450101 32527724

[7] Liu, K., Rehfus, J. E., Mattson, E. & Kaiser, C. M. The ribosome destabilizes native and non-native structures in a nascent multidomain protein. *Protein Sci.* **26**, 1439–1451 (2017). 10.1002/pro.3189 PMC5477528 28474852

[8] Wruck, F. et al. The ribosome modulates folding inside the ribosomal exit tunnel. *Commun. Biol.* **4**, 523 (2021). 10.1038/s42003-021-02055-8 PMC8100117 33953328

[9] Kaiser, C. M., Goldman, D. H., Chodera, J. D., Tinoco, I. Jr. & Bustamante, C. The ribosome modulates nascent protein folding. *Science* **334**, 1723–1727 (2011). 10.1126/science.1209740 PMC4172366 22194581

[10] Duran-Romaña, R. et al. Native fold delay and its implications for co-translational chaperone binding and protein aggregation. *Nat. Commun.* **16**, 1673 (2025). 10.1038/s41467-025-57033-z PMC11830000 39955309

[11] Waudby, C. A. et al. Systematic mapping of free energy landscapes of a growing filamin domain during biosynthesis. *Proc. Natl Acad. Sci. USA* **115**, 9744–9749 (2018). 10.1073/pnas.1716252115 PMC6166796 30201720

[12] Bhushan, S. et al. α-Helical nascent polypeptide chains visualized within distinct regions of the ribosomal exit tunnel. *Nat. Struct. Mol. Biol.* **17**, 313–317 (2010). 10.1038/nsmb.1756 20139981

[13] Deckert, A. et al. Common sequence motifs of nascent chains engage the ribosome surface and trigger factor. *Proc. Natl Acad. Sci. USA* **118**, e2103015118 (2021). 10.1073/pnas.2103015118 PMC8719866 34930833

[14] Knight, A. M. et al. Electrostatic effect of the ribosomal surface on nascent polypeptide dynamics. *ACS Chem. Biol.* **8**, 1195–1204 (2013). 10.1021/cb400030n 23517476

[15] Tan, R. et al. Folding stabilities of ribosome-bound nascent polypeptides probed by mass spectrometry. *Proc. Natl Acad. Sci. USA* **120**, e2303167120 (2023). 10.1073/pnas.2303167120 PMC10438377 37552756

[16] Ahn, M. et al. Modulating co-translational protein folding by rational design and ribosome engineering. *Nat. Commun.* **13**, 424310 (2022). 10.1038/s41467-022-31906-z PMC9307626 35869078

[17] Kudva, R. et al. The shape of the bacterial ribosome exit tunnel affects cotranslational protein folding. *Elife* **7**, e36326 (2018). 10.7554/eLife.36326 PMC6298777 30475203

[18] Guzman-Luna, V., Fuchs, A. M., Allen, A. J., Staikos, A. & Cavagnero, S. An intrinsically disordered nascent protein interacts with specific regions of the ribosomal surface near the exit tunnel. *Commun. Biol.* **4**, 1236 (2021). 10.1038/s42003-021-02752-4 PMC8556260 34716402

[19] Deuerling, E., Gamerdinger, M. & Kreft, S. G. Chaperone interactions at the ribosome. *Cold Spring Harb. Perspect. Biol.* **11**, a033977 (2019). 10.1101/cshperspect.a033977 PMC6824243 30833456

[20] Roeselova, A. et al. Mechanism of chaperone coordination during cotranslational protein folding in bacteria. *Mol Cell* **84**, 2455–2471 (2024). 10.1016/j.molcel.2024.06.002 38908370

[21] Kramer, G., Shiber, A. & Bukau, B. Mechanisms of cotranslational maturation of newly synthesized proteins. *Annu. Rev. Biochem.* **88**, 337–364 (2019). 10.1146/annurev-biochem-013118-111717 30508494

[22] Oh, E. et al. Selective ribosome profiling reveals the cotranslational chaperone action of trigger factor in vivo. *Cell* **147**, 1295–1308 (2011). 10.1016/j.cell.2011.10.044 PMC3277850 22153074

[23] Streit, J. O. et al. Long-range electrostatic forces govern how proteins fold on the ribosome. Preprint at *bioRxiv* 10.1101/2025.02.10.637539 (2025).

[24] Requiao, R. D. et al. Protein charge distribution in proteomes and its impact on translation. *PLoS Comput. Biol.* **13**, e1005549 (2017). 10.1371/journal.pcbi.1005549 PMC5460897 28531225

[25] Braselmann, E., Chaney, J. L. & Clark, P. L. Folding the proteome. *Trends Biochem. Sci* **38**, 337–344 (2013). 10.1016/j.tibs.2013.05.001 PMC3691291 23764454

[26] To, P., Whitehead, B., Tarbox, H. E. & Fried, S. D. Nonrefoldability is pervasive across the *E*. *coli* proteome. *J. Am. Chem. Soc.* **143**, 11435–11448 (2021). 10.1021/jacs.1c03270 PMC8650709 34308638

[27] Evans, M. S., Sander, I. M. & Clark, P. L. Cotranslational folding promotes β-helix formation and avoids aggregation in vivo. *J. Mol. Biol.* **383**, 683–692 (2008). 10.1016/j.jmb.2008.07.035 PMC2597226 18674543

[28] Frydman, J., Erdjument-Bromage, H., Tempst, P. & Hartl, F. U. Co-translational domain folding as the structural basis for the rapid de novo folding of firefly luciferase. *Nat. Struct. Biol.* **6**, 697–705 (1999). 10.1038/10754 10404229

[29] Plessa, E. et al. Nascent chains can form co-translational folding intermediates that promote post-translational folding outcomes in a disease-causing protein. *Nat. Commun.* **12**, 6447 (2021). 10.1038/s41467-021-26531-1 PMC8576036 34750347

[30] Alexander, L. M., Goldman, D. H., Wee, L. M. & Bustamante, C. Non-equilibrium dynamics of a nascent polypeptide during translation suppress its misfolding. *Nat. Commun.* **10**, 2709 (2019). 10.1038/s41467-019-10647-6 PMC6586675 31221966

[31] Bertolini, M. et al. Interactions between nascent proteins translated by adjacent ribosomes drive homomer assembly. *Science* **371**, 57–64 (2021). 10.1126/science.abc7151 PMC7613021 33384371

[32] Shiber, A. et al. Cotranslational assembly of protein complexes in eukaryotes revealed by ribosome profiling. *Nature* **561**, 268–272 (2018). 10.1038/s41586-018-0462-y PMC6372068 30158700

[33] Liutkute, M., Maiti, M., Samatova, E., Enderlein, J. & Rodnina, M. V. Gradual compaction of the nascent peptide during cotranslational folding on the ribosome. *Elife* **9**, e60895 (2020). 10.7554/eLife.60895 PMC7593090 33112737

[34] Liu, K., Maciuba, K. & Kaiser, C. M. The ribosome cooperates with a chaperone to guide multi-domain protein folding. *Mol. Cell* **74**, 310–319 (2019). 10.1016/j.molcel.2019.01.043 PMC6481645 30852061

[35] Holtkamp, W. et al. Cotranslational protein folding on the ribosome monitored in real time. *Science* **350**, 1104–1107 (2015). 10.1126/science.aad0344 26612953

[36] Wales, T. E. et al. Resolving chaperone-assisted protein folding on the ribosome at the peptide level. *Nat. Struct. Mol. Biol.* **31**, 1888–1897 (2024). 10.1038/s41594-024-01355-x PMC11638072 38987455

[37] Pellowe, G. A. et al. The human ribosome modulates multidomain protein biogenesis by delaying cotranslational domain docking. *Nat. Struct. Mol. Biol.* **32**, 2296–2307 (2025). 10.1038/s41594-025-01676-5 PMC12618258 40973728

[38] Chen, X., Rajasekaran, N., Liu, K. & Kaiser, C. M. Synthesis runs counter to directional folding of a nascent protein domain. *Nat. Commun.* **11**, 5096 (2020). 10.1038/s41467-020-18921-8 PMC7547688 33037221

[39] Cabrita, L. D. et al. A structural ensemble of a ribosome–nascent chain complex during cotranslational protein folding. *Nat. Struct. Mol. Biol.* **23**, 278–285 (2016). 10.1038/nsmb.3182 PMC5405865 26926436

[40] Burridge, C. et al. Nascent chain dynamics and ribosome interactions within folded ribosome-nascent chain complexes observed by NMR spectroscopy. *Chem. Sci.* **12**, 13120–13126 (2021). 10.1039/d1sc04313g PMC8513902 34745542

[41] Chan, S. H. S., Waudby, C. A. & Christodoulou, J. NMR snapshots of nascent chains emerging from the ribosome during biosynthesis. Preprint at *chemRxiv*10.26434/chemrxiv-2022-0lmsp (2022).

[42] Streit, J. O., Chan, S. H. S., Daya, S. & Christodoulou, J. Rational design of ^19^F NMR labelling sites to probe protein structure and interactions. *Nat. Commun.* **16**, 4300 (2025). 10.1038/s41467-025-59105-6 PMC12062419 40341366

[43] Huang, Y. et al. Environmentally ultrasensitive fluorine probe to resolve protein conformational ensembles by ^19^F NMR and cryo-EM. *J. Am. Chem. Soc.* **145**, 8583–8592 (2023). 10.1021/jacs.3c01003 PMC10119980 37023263

[44] Jackson, J. C., Hammill, J. T. & Mehl, R. A. Site-specific incorporation of a ^19^F-amino acid into proteins as an NMR probe for characterizing protein structure and reactivity. *J. Am. Chem. Soc.* **129**, 1160–1166 (2007). 10.1021/ja064661t 17263397

[45] Mirdita, M. et al. ColabFold: making protein folding accessible to all. *Nat. Methods* **19**, 679–682 (2022). 10.1038/s41592-022-01488-1 PMC9184281 35637307

[46] Abramson, J. et al. Accurate structure prediction of biomolecular interactions with AlphaFold 3. *Nature* **630**, 493–500 (2024). 10.1038/s41586-024-07487-w PMC11168924 38718835

[47] Krantz, B. A. & Sosnick, T. R. Engineered metal binding sites map the heterogeneous folding landscape of a coiled coil. *Nat. Struct. Biol.* **8**, 1042–1047 (2001). 10.1038/nsb723 11694889

[48] Camilloni, C., Broglia, R. A. & Tiana, G. Hierarchy of folding and unfolding events of protein G, CI2, and ACBP from explicit-solvent simulations. *J. Chem. Phys.* **134**, 045105 (2011). 10.1063/1.3523345 21280806

[49] Marchi, M. & Ballone, P. Adiabatic bias molecular dynamics: a method to navigate the conformational space of complex molecular systems. *J. Chem. Phys.* **110**, 3697–3702 (1999).

[50] Paci, E. & Karplus, M. Forced unfolding of fibronectin type 3 modules: an analysis by biased molecular dynamics simulations. *J. Mol. Biol.* **288**, 441–459 (1999). 10.1006/jmbi.1999.2670 10329153

[51] Ahn, M. et al. Amyloid forming human lysozyme intermediates are stabilized by non-native amide–π interactions. *Adv. Sci.* **12**, e03957 (2025). 10.1002/advs.202503957 PMC12442690 40557600

[52] Wlodarski, T. et al. Bayesian reweighting of biomolecular structural ensembles using heterogeneous cryo-EM maps with the cryoENsemble method. *Sci. Rep.* **14**, 18149 (2024). 10.1038/s41598-024-68468-7 PMC11300795 39103467

[53] Mitropoulou, A. N. et al. The ribosome directs nascent chains through two folding-dependent pathways. Preprint at *bioRxiv* 10.1101/2025.04.08.647855 (2025).

[54] Galmozzi, C. V. et al. Proteome-wide determinants of co-translational chaperone binding in bacteria. *Nat. Commun.* **16**, 4361 (2025). 10.1038/s41467-025-59067-9 PMC12065913 40348781

[55] Fowler, S. B. et al. Mechanical unfolding of a titin Ig domain: structure of unfolding intermediate revealed by combining AFM, molecular dynamics simulations, NMR and protein engineering. *J. Mol. Biol.* **322**, 841–849 (2002). 10.1016/s0022-2836(02)00805-7 12270718

[56] Fowler, S. B. & Clarke, J. Mapping the folding pathway of an immunoglobulin domain: structural detail from phi value analysis and movement of the transition state. *Structure* **9**, 355–366 (2001). 10.1016/s0969-2126(01)00596-2 11377196

[57] Lindorff-Larsen, K., Piana, S., Dror, R. O. & Shaw, D. E. How fast-folding proteins fold. *Science* **334**, 517–520 (2011). 10.1126/science.1208351 22034434

[58] Wright, C. F., Lindorff-Larsen, K., Randles, L. G. & Clarke, J. Parallel protein-unfolding pathways revealed and mapped. *Nat. Struct. Biol.* **10**, 658–662 (2003). 10.1038/nsb947 12833152

[59] Schwaiger, I., Kardinal, A., Schleicher, M., Noegel, A. A. & Rief, M. A mechanical unfolding intermediate in an actin-crosslinking protein. *Nat. Struct. Mol. Biol.* **11**, 81–85 (2004). 10.1038/nsmb705 14718927

[60] Bhatia, S., Krishnamoorthy, G. & Udgaonkar, J. B. Mapping distinct sequences of structure formation differentiating multiple folding pathways of a small protein. *J. Am. Chem. Soc.* **143**, 1447–1457 (2021). 10.1021/jacs.0c11097 33430589

[61] Radford, S. E., Dobson, C. M. & Evans, P. A. The folding of hen lysozyme involves partially structured intermediates and multiple pathways. *Nature* **358**, 302–307 (1992). 10.1038/358302a0 1641003

[62] Zaidi, F. N., Nath, U. & Udgaonkar, J. B. Multiple intermediates and transition states during protein unfolding. *Nat. Struct. Biol.* **4**, 1016–1024 (1997). 10.1038/nsb1297-1016 9406552

[63] Schwaiger, I., Schleicher, M., Noegel, A. A. & Rief, M. The folding pathway of a fast-folding immunoglobulin domain revealed by single-molecule mechanical experiments. *EMBO Rep.* **6**, 46–51 (2005). 10.1038/sj.embor.7400317 PMC1299227 15608615

[64] Khushoo, A., Yang, Z., Johnson, A. E. & Skach, W. R. Ligand-driven vectorial folding of ribosome-bound human CFTR NBD1. *Mol. Cell* **41**, 682–692 (2011). 10.1016/j.molcel.2011.02.027 PMC3095512 21419343

[65] Clarke, J., Cota, E., Fowler, S. B. & Hamill, S. J. Folding studies of immunoglobulin-like β-sandwich proteins suggest that they share a common folding pathway. *Structure* **7**, 1145–1153 (1999). 10.1016/s0969-2126(99)80181-6 10508783

[66] Duttler, S., Pechmann, S. & Frydman, J. Principles of cotranslational ubiquitination and quality control at the ribosome. *Mol. Cell* **50**, 379–393 (2013). 10.1016/j.molcel.2013.03.010 PMC3886275 23583075

[67] Keshwani, M. M. et al. Cotranslational cis-phosphorylation of the COOH-terminal tail is a key priming step in the maturation of cAMP-dependent protein kinase. *Proc. Natl Acad. Sci. USA* **109**, E1221–E1229 (2012). 10.1073/pnas.1202741109 PMC3356610 22493239

[68] Szeto, S. G. Y., Williams, E. C., Rudner, A. D. & Lee, J. M. Phosphorylation of filamin A by Cdk1 regulates filamin A localization and daughter cell separation. *Exp. Cell. Res.* **330**, 248–266 (2015). 10.1016/j.yexcr.2014.10.024 25445790

[69] Oh, W. J. et al. mTORC2 can associate with ribosomes to promote cotranslational phosphorylation and stability of nascent Akt polypeptide. *EMBO J.* **29**, 3939–3951 (2010). 10.1038/emboj.2010.271 PMC3020639 21045808

[70] Lang, B., Streit, J. O., Kriwacki, R. W., Christodoulou, J. & Babu, M. M. Protein dynamics at different timescales unlock access to hidden post-translational modification sites. Preprint at *bioRxiv* 10.1101/2025.06.25.661537 (2025).

[71] Urrutia, J. et al. An epilepsy-causing mutation leads to co-translational misfolding of the Kv7.2 channel. *BMC Biol.* **19**, 109 (2021). 10.1186/s12915-021-01040-1 PMC8138981 34020651

[72] Shishido, H., Yoon, J. S., Yang, Z. & Skach, W. R. CFTR trafficking mutations disrupt cotranslational protein folding by targeting biosynthetic intermediates. *Nat. Commun.* **11**, 4258 (2020). 10.1038/s41467-020-18101-8 PMC7450043 32848127

[73] Cassaignau, A. M. et al. A strategy for co-translational folding studies of ribosome-bound nascent chain complexes using NMR spectroscopy. *Nat. Protoc.* **11**, 1492–1507 (2016). 10.1038/nprot.2016.101 27466710

[74] Deckert, A. et al. Structural characterization of the interaction of α-synuclein nascent chains with the ribosomal surface and trigger factor. *Proc. Natl Acad. Sci. USA* **113**, 5012–5017 (2016). 10.1073/pnas.1519124113 PMC4983817 27092002

[75] Delaglio, F. et al. NMRPipe: a multidimensional spectral processing system based on UNIX pipes. *J. Biomol. NMR* **6**, 277–293 (1995). 10.1007/BF00197809 8520220

[76] Bezanson, J., Edelman, A., Karpinski, S. & Shah, V. B. Julia: a fresh approach to numerical computing. *SIAM Rev.* **59**, 65–98 (2017).

[77] Schanda, P., Kupce, E. & Brutscher, B. SOFAST-HMQC experiments for recording two-dimensional heteronuclear correlation spectra of proteins within a few seconds. *J. Biomol. NMR* **33**, 199–211 (2005). 10.1007/s10858-005-4425-x 16341750

[78] Barducci, A., Bussi, G. & Parrinello, M. Well-tempered metadynamics: a smoothly converging and tunable free-energy method. *Phys. Rev. Lett.* **100**, 020603 (2008). 10.1103/PhysRevLett.100.020603 18232845

[79] Piana, S., Donchev, A. G., Robustelli, P. & Shaw, D. E. Water dispersion interactions strongly influence simulated structural properties of disordered protein states. *J. Phys. Chem. B* **119**, 5113–5123 (2015). 10.1021/jp508971m 25764013

[80] Piana, S., Robustelli, P., Tan, D., Chen, S. & Shaw, D. E. Development of a force field for the simulation of single-chain proteins and protein–protein complexes. *J. Chem. Theory Comput.* **16**, 2494–2507 (2020). 10.1021/acs.jctc.9b00251 31914313

[81] Abraham, M. J. et al. GROMACS: high performance molecular simulations through multi-level parallelism from laptops to supercomputers. *SoftwareX* **1–2**, 19–25 (2015).

[82] consortium, P. Promoting transparency and reproducibility in enhanced molecular simulations. *Nat. Methods* **16**, 670–673 (2019). 10.1038/s41592-019-0506-8 31363226

[83] Tribello, G. A., Bonomi, M., Branduardi, D., Camilloni, C. & Bussi, G. PLUMED 2: new feathers for an old bird. *Comput. Phys. Commun.* **185**, 604–613 (2014).

[84] McCoy, A. J., Fucini, P., Noegel, A. A. & Stewart, M. Structural basis for dimerization of the *Dictyostelium* gelation factor (ABP120) rod. *Nat. Struct. Biol.* **6**, 836–841 (1999). 10.1038/12296 10467095

[85] Bussi, G., Donadio, D. & Parrinello, M. Canonical sampling through velocity rescaling. *J. Chem. Phys.* **126**, 014101 (2007). 10.1063/1.2408420 17212484

[86] Berendsen, H. J. C., Postma, J. P. M., Vangunsteren, W. F., Dinola, A. & Haak, J. R. Molecular dynamics with coupling to an external bath. *J. Chem. Phys.* **81**, 3684–3690 (1984).

[87] Parrinello, M. & Rahman, A. Polymorphic transitions in single-crystals—a new molecular-dynamics method. *J. Appl. Phys.* **52**, 7182–7190 (1981).

[88] Hess, B., Bekker, H., Berendsen, H. J. C. & Fraaije, J. G. E. M. LINCS: a linear constraint solver for molecular simulations. *J. Comput. Chem.* **18**, 1463–1472 (1997).

[89] Darden, T., York, D. & Pedersen, L. Particle mesh Ewald—an *N*·log(*N*) method for Ewald sums in large systems. *J. Chem. Phys.* **98**, 10089–10092 (1993).

[90] Melis, C., Bussi, G., Lummis, S. C. & Molteni, C. Trans-cis switching mechanisms in proline analogues and their relevance for the gating of the 5-HT_3_ receptor. *J. Phys. Chem. B* **113**, 12148–12153 (2009). 10.1021/jp9046962 PMC2733763 19663504

[91] Tiwary, P. & Parrinello, M. A time-independent free energy estimator for metadynamics. *J. Phys. Chem. B* **119**, 736–742 (2015). 10.1021/jp504920s 25046020

[92] Noel, J. K. et al. SMOG 2: a versatile software package for generating structure-based models. *PLoS Comput. Biol.* **12**, e1004794 (2016). 10.1371/journal.pcbi.1004794 PMC4786265 26963394

[93] Best, R. B., Hummer, G. & Eaton, W. A. Native contacts determine protein folding mechanisms in atomistic simulations. *Proc. Natl Acad. Sci. USA* **110**, 17874–17879 (2013). 10.1073/pnas.1311599110 PMC3816414 24128758

[94] Tiana, G. & Camilloni, C. Ratcheted molecular-dynamics simulations identify efficiently the transition state of protein folding. *J. Chem. Phys.* **137**, 235101 (2012). 10.1063/1.4769085 23267502

[95] Wang, F. et al. All-atom simulations reveal how single-point mutations promote serpin misfolding. *Biophys. J.* **114**, 2083–2094 (2018). 10.1016/j.bpj.2018.03.027 PMC5961751 29742402

[96] Tucker, M. R., Piana, S., Tan, D., LeVine, M. V. & Shaw, D. E. Development of force field parameters for the simulation of single- and double-stranded DNA molecules and DNA–protein complexes. *J. Phys. Chem. B* **126**, 4442–4457 (2022). 10.1021/acs.jpcb.1c10971 PMC9234960 35694853

[97] Hopkins, C. W., Le Grand, S., Walker, R. C. & Roitberg, A. E. Long-time-step molecular dynamics through hydrogen mass repartitioning. *J. Chem. Theory Comput.* **11**, 1864–1874 (2015). 10.1021/ct5010406 26574392

[98] Daura, X. et al. Peptide folding: when simulation meets experiment. *Angew. Chem. Int. Ed. Engl.* **38**, 236–240 (1999).

[99] McGibbon, R. T. et al. MDTraj: a modern open library for the analysis of molecular dynamics trajectories. *Biophys. J.* **109**, 1528–1532 (2015). 10.1016/j.bpj.2015.08.015 PMC4623899 26488642

[100] Michaud-Agrawal, N., Denning, E. J., Woolf, T. B. & Beckstein, O. MDAnalysis: a toolkit for the analysis of molecular dynamics simulations. *J. Comput. Chem.* **32**, 2319–2327 (2011). 10.1002/jcc.21787 PMC3144279 21500218

[101] Huang, J. et al. CHARMM36m: an improved force field for folded and intrinsically disordered proteins. *Nat. Methods* **14**, 71–73 (2017). 10.1038/nmeth.4067 PMC5199616 27819658

[102] Yang, D. T., Gronenborn, A. M. & Chong, L. T. Development and validation of fluorinated, aromatic amino acid parameters for use with the AMBER ff15ipq protein force field. *J. Phys. Chem. A* **126**, 2286–2297 (2022). 10.1021/acs.jpca.2c00255 PMC9014858 35352936

[103] Debiec, K. T. et al. Further along the road less traveled: AMBER ff15ipq, an original protein force field built on a self-consistent physical model. *J. Chem. Theory Comput.* **12**, 3926–3947 (2016). 10.1021/acs.jctc.6b00567 PMC4980686 27399642

[104] van der Spoel, D., van Maaren, P. J., Larsson, P. & Timneanu, N. Thermodynamics of hydrogen bonding in hydrophilic and hydrophobic media. *J. Phys. Chem. B* **110**, 4393–4398 (2006). 10.1021/jp0572535 16509740

[105] Luzar, A. & Chandler, D. Hydrogen-bond kinetics in liquid water. *Nature* **379**, 55–57 (1996).

[106] Solomon, I. & Bloembergen, N. Nuclear magnetic interactions in the HF molecule. *J. Chem. Phys.* **25**, 261–266 (1956).

[107] Meng, E. C. et al. UCSF ChimeraX: tools for structure building and analysis. *Protein Sci.* **32**, e4792 (2023). 10.1002/pro.4792 PMC10588335 37774136

[108] Chan, S. et al. Molecular dynamics simulations of co-translational protein folding intermediates on the ribosome. *Zenodo*10.5281/zenodo.16601044 (2025).

[109] Chan, S. et al. NMR dataset—Chan et al. 2026: Structures of protein folding intermediates on the ribosome. *Zenodo*10.5281/zenodo.19210765 (2026). PMC13275320 42304112

[110] Chan, S. shschan/NMR-fit: v1.0.0. *Zenodo*10.5281/zenodo.15169088 (2025).

[111] Streit, J. julian-streit/RingCurrents19F: v1.0.0. *Zenodo*10.5281/zenodo.15173797 (2025).

