# Supplementary material for: Structures of protein folding intermediates on the ribosome
Source: Nat Struct Mol Biol. 2026 Jun 16;33(6):962–72. doi: 10.1038/s41594-026-01814-7 (PMC13275320; doi:10.1038/s41594-026-01814-7)
Supplement: Supplementary file 1 — Supplementary Notes 1–9 and Tables 1–5. [file 41594_2026_1814_MOESM1_ESM.pdf]

nature structural & molecular biology

Supplementary information https://doi.org/10.1038/s41594-026-01814-7

Structures of protein folding intermediates on the ribosome

In the format provided by the authors and unedited

Supplementary Information

Supplementary Note 1: ^{19}F NMR of intermediate states on the ribosome

The presence and number of broad ^{19}F NMR resonances attributable to intermediate states was confirmed through a series of analyses and additional experiments. In addition to quantitative lineshape analyses in the frequency domain (**Figure 1h**, **Extended Data Figure 2a**), the number of peaks fitted in each spectrum was confirmed by statistical tests of fits performed on data analysed in the time domain[1] (**Methods, Supplementary Table 3**). The RNC spectra were additionally compared against those measured for RNCs of a fully unfolded, folding-incompetent mutant (FLN5+47 Tyr719Glu) labelled at the same site (**Extended Data Figure 2a**), which showed a narrower linewidth for the unfolded state (compared to the putative intermediate state resonances, **Supplementary Table 1**). We also incubated FLN5+47 tfmF718 671Phe RNCs in mild denaturant (2.5 M urea, **Extended Data Figure 2a**). Under these conditions, the folding equilibrium is expected to be shifted, and indeed, four NMR peaks were observed with the additional resonance attributable to the unfolded state, having a narrow linewidth (~11 Hz) and a random coil chemical shift (**Extended Data Figure 2a**). The remaining resonances showed reduced integrals (i.e. populations) and line broadening as expected (**Supplementary Tables 1-2**), the latter resulting from reduced interactions between the nascent protein and ribosome surface in urea, and which improved resolution between the overlapped resonances. Finally, the two broad resonances were also found at a shorter RNC length, with broader linewidths resulting from increased ribosome surface interactions closer to the ribosome as expected[2] (**Extended Data Figure 2a**). Similar analyses and experiments were performed for the remaining ^{19}F-label sites (**Extended Data Figure 2a, Supplementary Tables 1-3**). Collectively, the data and analyses confirm the presence of broad resonances attributable to two distinct intermediate states across the ^{19}F NMR spectra of RNCs.

Supplementary Note 2: Ratchet-and-pawl simulations and starting structures of unbiased MD simulations

We used all-atom MD simulations as an orthogonal means to assess the coTF intermediate structures and to obtain starting structures for subsequent unbiased MD simulations. We initially performed ratchet-and-pawl (rMD) simulations[3,4] using the DES-Amber force-field in explicit solvent[5-7] (**Methods**) to model the sequence of folding events from an unfolded

FLN5+47 RNC to a native-like conformation (**Supplementary Videos 1-2**, **Extended Data Figure 6**). Despite specifying a native target structure, we note that non-native contacts and features can also be captured by rMD simulations[8,9]. While rMD cannot predict thermodynamic properties of folding in different environments (e.g., on/off the ribosome), we used these sampled transition paths to obtain putative insights into the structures of metastable folding intermediates.

Analysis of the folding trajectories showed a largely hierarchical folding pathway, where β-strand pairs are progressively formed (**Extended Data Figure 6b-d**). Crucially, we identified a local minimum within the transition path energy landscape, corresponding to an intermediate state. The structural ensemble possessed a native-like core comprising the C- to G-strands, and was found on-pathway and prior to the folding of the remaining N-terminal A-B strands to form the fully native state (**Extended Data Figure 6b-d**). Additional calculations show that native G-strand folding is thermodynamically dependent on the isomerisation state of the conserved natively cis-Pro742 (**Extended Data Figure 5**); within this intermediate, Pro742 is therefore expected to adopt a cis-conformation, consistent with experimental measurements showing its strong destabilisation by the Pro742Ala mutation[1]. Indeed, free energy calculations of Pro742 isomerisation were used to derive a $\Delta\Delta G_{N\text{-}U,cis\text{-}trans}$ (i.e., the energetic contribution of cis Pro742 to the folding free energy compared to the trans state). The obtained value is quantitatively consistent with the experimental value for $\Delta\Delta G_{N\text{-}U}$ comparing wild-type and P742A FLN5 (3.70 ± 0.38 versus 4.00 ± 0.10 kcal mol^{-1}, respectively, **Extended Data Figure 5n**). The observed structural features for the intermediate sampled in the rMD simulations thus appeared to align most closely with NMR data of the I1 state (**Figure 2a-b**, and ref.[1]), and we therefore assessed this intermediate state further by sampling the structures and dynamics from the folding trajectories in additional MD simulations (**Figure 3, Extended Data Figure 7**).

In contrast, we could not identify a local energy minimum corresponding to a potential I2 state (**Extended Data Figure 6b-f**), likely due to methodological limitations of the rMD approach, for example, previously reported overstabilisation of local, short-range contacts[8]. This suggests that rMD is less sensitive to intermediate states that only differ from the native state by a few local contacts (i.e., structural elements with low contact order like the F-G strand

pair of FLN5). However, given the structural similarity of I2 to a folding intermediate populated by an isolated, truncated variant of FLN5[10] (**Figure 2c-d**), as also found from previous coarse-grained structure-based models[1], we instead used previously determined NMR structures of the isolated intermediate[10], having a disordered G-strand and Pro742 in the trans conformation[10] (**Extended Data Figure 5**) as starting models of MD simulations. We tethered the isolated intermediate to the ribosome ensuring the same sequence as FLN5+47 (**Methods**). Extensive sampling of the structure and dynamics of the I1 and I2 models were subsequently performed using unbiased all-atom MD simulations (**Figure 3, Extended Data Figure 7, Supplementary Videos 3-4**)

Lastly, we note that the observed folding pathway in rMD simulations and sampling of the I1 intermediate state was robust with respect to the choice of biasing force constant (**Extended Data Figure 6i-k**).

Supplementary Note 3: Assessing intermediate structure distance distributions against chemical shift data

To assess our structural ensembles, we calculated distance distributions between pairs of residues that were used for ^{19}F-labelling (typically C_β-C_β atomic distances), and compared these analyses to both those of the native structure[11] and also the experimental chemical shifts, as the latter are engineered to probe the inter-residue contact/distance of the label pair (**Figures 1f-h**, **Extended Data Figure 2a**). This simple analysis rationalised almost all of the observed chemical shifts.

More accurate comparisons can be made by explicitly modelling the ^{19}F-label pair (tfmF and the accompanying aromatic residue) and calculating the predicted geometric factor $(1-3\cos^2\theta)/r^3$, where r and θ are the distance and angle of the CF_3 group in tfmF relative to the aromatic ring. We first modelled the native and I1 intermediate state with ^{19}F-label pairs across the F-G strands. Both label pairs (726tfmF 746Phe and 728tfmF 744His) yielded similar geometric factors for the I1 and native states (**Extended Data Figure 7k**), as also reflected by their inter-residue C_β-C_β distances measured without explicit ^{19}F-label pairs (**Figure 3h**) and by the experimental data (chemical shifts and bihistidine metal binding, **Figures 1-2**). In contrast, assessing the D-E strand contacts (710tfmF 716His and 718tfmF

706His), we found substantially reduced (>50%) geometric factors for I1 relative to the native state (**Extended Data Figure 7k**) for both labelling sites, but native-like C_β-C_β (C_α for Gly716) distances calculated for their structures without explicit ^{19}F-label pairs (**Figure 3h**). These calculations indicate that the D-E strands form native-like secondary structures in I1, with more subtle sidechain conformational changes that result in reduced ring current interactions (relative to native state), and accounting for their random coil chemical shifts.

For natively buried probes (727tfmF, 715tfmF) whose chemical shifts report on specific side chain-amide backbone contacts (**Extended Data Figure 3**), we calculated hydrogen bond propensities (**Extended Data Figure 7l-m**) and found that native interactions remained in I2 structures, but not in I1, reflective of their extent of structure formation and indeed their chemical shifts.

Supplementary Note 4: Ribosome interactions, cross-linking experiments and cryo-EM fits

From the MD simulations, we find that the calculated rotational correlation times of I1 and I2 and are slower compared to the native state (**Extended Data Figure 7k**), which is reflected experimentally by their increased line broadening of their ^{19}F NMR resonances[1,12] (relative to the native state, **Supplementary Table 1**); we note ^{19}F CEST and $R_{1\rho}$ measurements exclude chemical exchange contributions to the broadening[2]. Locally enhanced binding occurs at their more positively charged N-termini and the loop between the B-C strands, biasing their orientations with the ribosome (**Extended Data Figure 7h**), consistent with charge reversal mutations in these regions (Lys646Glu Lys680Glu) showing destabilisation of both I1 and I2 (ref.[1]). The detached C-terminal G-strand in I2 also interacts more strongly with the ribosome surface (**Extended Data Figure 7h**), a region that has been experimentally shown to bind and stabilise unfolded FLN5 on the ribosome[2].

We explored the presence of intermolecular interactions further by directly examining specific contacts between nascent chain residues and ribosomal protein uL24 that lines the exit tunnel using biochemical cross-linking assays. Our structures showed that all states (I1, I2, N) do not substantially contact uL24 via the N-terminal residue Ala648 (<5% within 2 nm**, Extended Data Figure 8d**); accordingly, we did not detect cross-links between FLN5+47 RNC Ala648Cys and uL24 (Gly90Cys) on CRISPR-modified ribosomes (**Extended Data Figure 8e**). In contrast, cross-links between uL24 (Gly90Cys) and FLN5+47 RNC labelled at

Asp699Cys were found, and could be rationalised by the increased contact (~8% within 2 nm) as observed in the I1 structures (**Extended Data Figure 8d-e**). Thus, these experiments also showed good agreement between (un)observed crosslinks, and intermolecular contacts calculated from the structural ensembles (**Extended Data Figure 8d-e**).

Our coTF intermediate structural ensembles were further assessed against cryogenic electron microscopy (cryo-EM) data. We have previously obtained cryo-EM maps of FLN5+47 and fitted natively folded FLN5 conformations to the densities[13]. However, slow chemical exchange of the native state with both I1 and I2 (ref.[1]) indicates that each conformation likely contributes to the observed cryo-EM densities. Therefore, we firstly compared cross-correlations between single structures within our three structural ensembles (native[11], and I1 and I2 states) with the maps; high cross-correlations were obtained when fitting the intermediate state structures, which were similar or better to those obtained using native state conformations (**Extended Data Figure 8a**), supporting the validity of our models. In an orthogonal approach, we used the cryo-EM maps to reweight the structural ensembles of I1, I2, and N using cryoENsemble[14]. Cross-correlations improved when combining all three ensembles together for reweighting (**Extended Data Figure 8b-c**), compared to individual ensembles (**Extended Data Figure 8c**), indicating that the densities most likely derive from heterogenous populations of RNCs of different conformational states, therefore crucially showing that addition of our I1 and I2 models improved the overall fit.

Altogether, the NMR linewidths, biochemical data and cryo-EM densities support the determined ensembles of intermediate structures, their ribosome interaction sites, dynamics, and orientations.

Supplementary Note 5: ^{19}F PRE measurements as validation of structural models

The paramagnetic effect results in substantial line broadening in the ^{19}F NMR spectrum of the RNC (**Figure 4c, Extended Data Figure 9b**), as expected, and indicative of Ni(II) binding to the nascent protein as designed. While it is evident from the raw spectra that there is only a minimal PRE effect on the I1 resonance, with a significantly greater effect on the I2 state, the increased line broadening results in severe overlap of resonances, complicating quantitative lineshape analyses. We note that this could not be alleviated by improving the signal-to-noise; indeed, the paramagnetic spectra of the RNCs are each summed from 5-6

separate samples (~100 h total acquisition time per PRE-label). Restraints were therefore required to fit lineshapes to the paramagnetic spectrum. We calculated the expected PRE rate of the native state in FLN5+47, using the experimental PRE rate measured for the isolated, natively folded protein (**Extended Data Figure 9a**) and scaled this with the rotational correlation time on the RNC, since the native structure is the same on/off the ribosome[11,15]. The rotational correlation time was determined using linewidths measured from the diamagnetic spectra (**Extended Data Figure 9c**) and a previously empirically determined correlation between these two parameters[12,15]. The expected PRE rate was then converted to a linewidth (with propagated errors) of the native state resonance in the paramagnetic spectrum, permitting free fits of the I1 and I2 lineshapes (**Figures 4c, Extended Data Figure 9b**). We then converted the measured linewidths of I1 and I2 to a PRE rate (**Extended Data Figure 9d**) and distance (**Figure 4d**). Distances were calculated using the rotational correlation time (measured in the same manner as for the native state), and assuming that the interaction vector in the molecular frame is the same across I1, I2, and the native state (**Methods**). From these calculations, we find the distance between 655tfmF and 673Cys-MTSL in I2 to be native-like, as expected from the 655tfmF chemical shift (probing Phe675, **Figure 1f**), validating our approach.

We compared the experimental distances to those calculated from the determined structural ensembles. As the rotamer distribution of the flexible MTSL spin label cannot be accurately determined (even when explicitly modelled), we instead calculated the distance between OH of 655tfmF and the C_β of the residue bearing the spin label. Comparison between the experimental and calculated distances for the native state (**Figure 4d**) indicate a small systematic (scaled) discrepancy, with the calculated distance being slightly underestimated for 675Cys-MTSL label, but overall good agreement, showing greater distances for I1 (relative to native) but native-like distances for I2.

The dramatic structural differences of I1 and I2 compared to the native state (~40% amino acids detached from the folded core for I1), together with their slow chemical exchange, show that they constitute bona fide structural states rather than substates of the native ensembles (e.g., minor terminal fraying).

Supplementary Note 6: Parallel folding pathways

The structures of the intermediates reveal a common folded core, comprising the C-F strands, with distinct conformations at their termini, having either native-like or detached β-strands. The progressive formation of β-strands in different temporal orders suggests that I1 and I2 populate parallel folding pathways towards the native state, as seen in folding off the ribosome[16,17].

The structural interpretation of parallel folding pathways is further supported by several experimental, computational, and literature datasets. Firstly, quantitative ^{19}F NMR spectra show simultaneous population of I1 and I2 at different FLN5 RNC lengths (**Figure 1d**, and ref.[1]), in contrast to the clear step-wise, sequential population of I1 before I2 observed for lengths of HRAS on the ribosome[12]. The ability to alter the flux in folding to selectively stabilise I1, using bihistidine metal chelation (**Figure 2**) or TF binding (**Figure 5**), or I2, by the Pro742Ala mutation[12], is also consistent with parallel folding routes. Our rMD simulations (**Extended Data Figure 6**) also map trajectories from the unfolded to I1 directly to the native states, and the absence of I2 in any folding pathway suggests a parallel route that cannot be captured by such methods.

To directly probe interchange between different conformational states, we performed chemical exchange saturation transfer (CEST) measurements of FLN5+34 (previously) and FLN5+47 RNC (this work, **Extended Data Figure 1b-c**). Notwithstanding the limited signal-to-noise sensitivity of such measurements, we find exchange between all states to occur slower than quantifiable by CEST (including U-N), with the potential exception of I2-N exchange, occurring at 1.3 ± 1.1 and 4.9 ± 1.1 s^{-1} for FLN5+34 and FLN5+47 RNC respectively (**Extended Data Figure 1b-c**). The reversible, faster interchange of these states suggest that I2 is not an off-pathway species. Indeed, I2 structurally resembles the intermediate state of a truncated variant of isolated FLN5, which has been found to convert directly between both its unfolded and natively folded states[10]. A comparative analysis to isolated folding pathways can also be made for I1, whose detached A-B strands of an otherwise native-like conformation resembles that of an obligate intermediate of FLN4 detected by atomic force microscopy (AFM), and was found to directly convert from the unfolded to native states without populating alternative (e.g. I2-like) intermediates[18].

Moreover, previous studies have found distinct subset of contacts formed in folding transition states or intermediates to correlate with distinct folding pathways, for multiple protein systems, including monellin[19], lysozyme[20], titin I27 (also studied in this work, and ref.[17]), and barstar[21].

Collectively, these experimental and computational data alongside comparisons to isolated protein pathways are more likely to support I1 and I2 being along parallel rather than sequential pathways.

Supplementary Note 7: Intermediate structures of I27

We performed all-atom MD simulations to model structures of an isolated I27 intermediate analogue[22]. The intermediate structure comprises a well-folded A'-G core with a detached A-strand, as expected[22,23] (**Extended Data Figure 10d**), permitting closer interactions between A'-G strands; this is reflected by quantitatively stronger ring current interactions between 14tfmF and 87His (relative to native state, **Extended Data Figure 10e**), and which therefore rationalises the observed highly shielded ^{19}F chemical shift of the isolated and ribosome-bound intermediate (**Figures 6d, Extended Data Figure 10c**). Indeed, agreement between another ^{19}F chemical shift of I27 RNCs with calculated ring current contacts across the isolated intermediate models (59tfmF, **Extended Data Figure 10**) suggest that the same intermediate structure is populated on the ribosome. Meanwhile, the chemical shifts of the remaining intermediate (I1) state resonances (**Extended Data Figure 10c**) indicate a less ordered structure, although overall maintaining a native-like hydrophobic core[12] (**Extended Data Figure 10c**), with inter-strand contacts identical to those observed in FLN5 I1 and thus suggestive of a similar structure.

Supplementary Note 8: Partial conservation of coTF intermediates of I27

In contrast to the filamin domains, titin I27 adopts a distinct I2 intermediate and likely folds sequentially on the ribosome (**Figure 6d-e**), consistent with its smaller size, altered topology, and lack of the conserved cis-proline. Thus, the I2 intermediate structure and proposed parallel folding pathways identified here appear to be (only) conserved within filamins. This

conservation contrasts with refolding studies of isolated filamin domains, which show distinct mechanisms[24], and highlights the ribosome's role in thermodynamically buffering sequence variation[12] and biasing similar folds toward a common co-translational pathway. Interestingly, I27 has been reported to refold via two distinct, parallel transition states and pathways, in contrast to a sequential pathway on the ribosome (this work), and whose flux is altered with denaturant concentration and mutations[17]; in a similar manner, the ribosome appears to alter the flux towards specific protein folding pathways, and which can be further modulated by interactions with molecular chaperones (**Figure 5**).

Supplementary Note 9: Thermodynamic basis of coTF intermediates

The thermodynamic basis for stable coTF intermediates originates from the destabilisation of the unfolded state[12], and more extensively, folded states[11], via long-range electrostatic interactions with the ribosome. As the folded regions of partially folded intermediates typically have a lower net negative charge (compared to the whole domain), they are likely to be less destabilised electrostatically. Hierarchical folding via intermediates may therefore be one means to minimise repulsive negatively charged globular structures near the ribosome surface, without complete unfolding of the nascent protein (**Supplementary Table 5**). Our structures also highlight the range of intermediate conformational states that can be stabilised. The high degree of disorder in the I1 coTF intermediate indicates that conformational entropy substantially contributes to its thermodynamic stability. In contrast, the stability of the more native-like I2 structure is likely to benefit from larger enthalpic contributions from intra- and intermolecular interactions. The distinct structures of I1 and I2, yet similar folding free energies[1], indicate that the loss of extensive solvation of the unfolded state to form (partially) folded structures can be compensated through a complex array of different entropic or enthalpic contributions.

In multi-domain proteins, structurally expanded (relative to native states) intermediate states may also thermodynamically stabilise preceding (fully translated) domains, since this would effectively increase the distance of the preceding domain from the repulsive ribosome surface[11], while also limiting exposure of misfolding-prone unfolded states.

Label	RNC	N (Hz)	±	I2 (Hz)	±	I1 (Hz)	±	U (Hz)	±
655tfmF	FLN5+110	12.2	0.4	67.1	3.6	207.1	21.6	-	-
655tfmF	FLN5+47	20.3	1.9	147.3	18.4	272.7	20.3	-	-
673tfmF 716His	FLN5+47	13.9	2.8	119.2	22.8	207.8	39.7	-	-
710tfmF 716His	FLN5+47	25.6	2.2	344.8	30.6	127.9	17.3	-	-
718tfmF 671Phe	FLN5+47	17.9	0.5	185.7	10.9	184.3	25.2	-	-
	FLN5+47 in urea	11.3	1.3	80.3	8.7	138.3	39.7	10.8	2.7
	FLN5+34	54.9	4.0	516.1	47.4	167.3	42.9	-	-
	FLN5+34 in urea	55.8	3.7	549.7	383.1	119.9	27.3	21.2	2.5
	FLN5+34	-	-	-	-	-	-	42.3	0.8
718tfmF 706His	FLN5+47	24.2	0.7	276.7	11.4	162.8	15.9	-	-
	FLN5+47 in urea	26.8	4.4	85.6	22.5	152.3	11.5	17.6	0.5
726tfmF 746Phe	FLN5+47	16.9	0.9	281.5	16.9	128.9	6.5	-	-
	FLN5+47 in urea	37.5	5.3	129.4	34.4	148.1	19.5	51.5	12.0
	FLN5+34	57.0	3.4	255.2	21.7	415.6	27.8	-	-
	FLN5+34 in urea	58.7	18.0	133.3	10.6	219.9	92.9	37.1	10.8
	FLN5+34	-	-	-	-	-	-	184.0	4.5
728tfmF 744His	FLN5+47	25.2	1.5	218.7	17.3	147.9	10.1	-	-
732tfmF	FLN5+47	28.2	1.8	351.2	23.6	-	-	112.4	47.8
	FLN5+47 in urea	14.9	1.4	229.5	21.6	-	-	79.6	5.0
	FLN5+34	42.7	4.3	684.5	132.1	-	-	203.4	56.4
	FLN5+34 in urea	9.9	4.3	379.9	30.9	-	-	78.1	7.9
	FLN5+34	-	-	-	-	-	-	267.5	11.1
715tfmF	FLN5+67	66.2	2.2	365.5	27.8	281.9	14.2	12.8	1.1
727tfmF	FLN5+67	36.0	1.0	227.2	24.5	176.4	14.5	21.8	11.6

Supplementary Table 1 Linewidth measurements from lineshape analyses of FLN5 RNCs. Linewidths determined from lineshape analysis of spectra shown in Figure 1 and Extended Data Figure 2. Errors were determined by bootstrapping of residuals from lineshape fits.

Label	RNC	N	±	I2	±	I1	±	U	±
655tfmF	FLN5+110	0.38	0.01	0.46	0.03	0.16	0.01	-	-
655tfmF	FLN5+47	0.19	0.02	0.38	0.06	0.44	0.03	-	-
673tfmF 716His	FLN5+47	0.17	0.03	0.50	0.09	0.34	0.05	-	-
710tfmF 716His	FLN5+47	0.19	0.01	0.53	0.06	0.27	0.03	-	-
718tfmF	FLN5+47	0.28	0.01	0.56	0.03	0.16	0.02	-	-
	FLN5+47 in urea	0.21	0.02	0.52	0.05	0.20	0.04	0.07	0.01
	FLN5+34	0.33	0.02	0.51	0.09	0.17	0.04	-	-
	FLN5+34 in urea	0.32	0.02	0.11	0.08	0.30	0.08	0.27	0.04
718tfmF 706His	FLN5+47	0.25	0.01	0.59	0.03	0.16	0.01	-	-
	FLN5+47 in urea	0.15	0.03	0.22	0.06	0.32	0.02	0.31	0.01
726tfmF	FLN5+47	0.19	0.01	0.36	0.02	0.45	0.03	-	-
	FLN5+47 in urea	0.24	0.05	0.33	0.11	0.28	0.07	0.14	0.04
	FLN5+34	0.24	0.01	0.27	0.02	0.49	0.05	-	-
	FLN5+34 in urea	0.12	0.04	0.59	0.07	0.20	0.11	0.08	0.03
728tfmF 744His	FLN5+47	0.24	0.01	0.26	0.02	0.51	0.04	-	-
732tfmF	FLN5+47	0.22	0.01	0.67	0.11	-	-	0.11	0.04
	FLN5+47 in urea	0.14	0.01	0.45	0.04	-	-	0.42	0.02
	FLN5+34	0.18	0.02	0.51	0.20	-	-	0.31	0.10
	FLN5+34 in urea	0.03	0.01	0.61	0.07	-	-	0.36	0.03
715tfmF	FLN5+67	0.34	0.01	0.23	0.02	0.31	0.02	0.12	0.01
727tfmF	FLN5+67	0.46	0.01	0.27	0.03	0.25	0.02	0.02	0.01

Supplementary Table 2 Fractional populations from lineshape analyses of FLN5 RNCs. Populations determined from integrals of lineshapes fitted for spectra shown in Figure 1 and Extended Data Figure 2. Errors were determined by bootstrapping of residuals from lineshape fits.

Label	RNC	Time-domain fit BIC		
		2-state	3-state	4-state
673tfmF 716His	FLN5+47	22215	**22206**	22228
710tfmF 716His	FLN5+47	24960	**24901**	24907
718tfmF	FLN5+47	23313	**23258**	23282
	FLN5+47 in	18665	18057	**17979**
	FLN5+34	20136	**20140**	20160
	FLN5+34 in	20770	**17149**	17161
718tfmF 706His	FLN5+47	23640	**23427**	*
	FLN5+47 in	22571	21910	**21732**
726tfmF	FLN5+47	23912	**23360**	23382
	FLN5+47 in	21906	21902	**21852**
	FLN5+34	23919	**23814**	23815
	FLN5+34 in	21137	21121	**21080**
728tfmF 744His	FLN5+47	23012	**22722**	22742
732tfmF	FLN5+47	**19339**	19355	*
	FLN5+47 in	17263	**17141**	*
	FLN5+34	**19267**	19287	*
	FLN5+34 in	14687	**14619**	*
715tfmF	FLN5+67	30314	28851	**28310**
727tfmF	FLN5+67	29544	29192	**29160**

Supplementary Table 3 Time-domain analysis. Bayesian information criterion (BIC) values calculated for NMR data, displayed in the frequency domain in Figure 1 and Extended Data Figure 2, analysed in the time-domain using different number of possible resonances, as previously described[1]. The lowest BIC values (in bold) indicate the most likely model. (*) indicates no reasonable fit could be made.

I1 cluster	Population (%)	I2 Cluster	Population (%)
Cluster_0001	22.5	Cluster_0001	25.4
Cluster_0002	11.1	Cluster_0002	10.3
Cluster_0003	8.8	Cluster_0003	5.3
Cluster_0004	5.0	Cluster_0004	4.1
Cluster_0005	4.9	Cluster_0005	2.8
Cluster_0006	3.7	Cluster_0006	2.4
Cluster_0007	3.4	Cluster_0007	2.1
Cluster_0008	2.4	Cluster_0008	1.8
Cluster_0009	2.2	Cluster_0009	1.7
Cluster_0010	2.1	Cluster_0010	1.3
Cluster_0011	1.9	Cluster_0011	1.0
Cluster_0012	1.7	Cluster_0012	1.0
Cluster_0013	1.4	Cluster_0013	0.8
Cluster_0014	1.3	Cluster_0014	0.8
Cluster_0015	1.2	Cluster_0015	0.7
Cluster_0016	1.2	Cluster_0016	0.7
Cluster_0017	1.1	Cluster_0017	0.7
Cluster_0018	1.0	Cluster_0018	0.7
Cluster_0019	1.0	Cluster_0019	0.6
Cluster_0020	1.0	Cluster_0020	0.6

Supplementary Table 4 Populations of top 20 clusters. Obtained with a RMSD cut-off of 0.2 and 0.1 nm for I1 and I2, respectively.

Net charge at pH 7.5 (residues)	FLN5	FLN4	FLNa21	I27
Full-length	-9 (K646-G750)	-13 (P547-A648)	-7 (G2236-P2328)	-6 (L1-L89)
I1 (C-G strand folded)	-5 (F691-G750)	-8 (F591-A648)	-7 (L2271-P2328)	-1 (V30-L89)
I2 (A-F strand folded for filamins, A'-G strand folded for I27)	-10 (K646-L733)	-13 (P547-L631)	-4 (G2236-F2311)	-5 (V11-L89)

Supplementary Table 5 Net charge of proteins studied and their intermediate state folded fragments. Lys/Arg are considered to have a charge of +1, and Asp/Glu have a charge of -1. The favourable ribosome interactions for I2 mediated by FLN5 residues Lys646/Lys680 are structurally conserved in FLN4 (Lys553/Arg583), and FLNa21 (Lys2240/Arg2264). Parentheses in the table indicate residues of folded regions.

References

1 Chan, S. H. S. *et al.* The ribosome stabilizes partially folded intermediates of a nascent multi-domain protein. *Nature Chemistry* **14**, 1165-1173, doi:10.1038/s41557-022-01004-0 (2022).

2 Cassaignau, A. M. E. *et al.* Interactions between nascent proteins and the ribosome surface inhibit co-translational folding. *Nat Chem* **13**, 1214-1220, doi:10.1038/s41557-021-00796-x (2021).

3 Camilloni, C., Broglia, R. A. & Tiana, G. Hierarchy of folding and unfolding events of protein G, CI2, and ACBP from explicit-solvent simulations. *J Chem Phys* **134**, 045105, doi:10.1063/1.3523345 (2011).

4 Tiana, G. & Camilloni, C. Ratcheted molecular-dynamics simulations identify efficiently the transition state of protein folding. *J Chem Phys* **137**, 235101, doi:10.1063/1.4769085 (2012).

5 Piana, S., Robustelli, P., Tan, D., Chen, S. & Shaw, D. E. Development of a Force Field for the Simulation of Single-Chain Proteins and Protein-Protein Complexes. *J Chem Theory Comput* **16**, 2494-2507, doi:10.1021/acs.jctc.9b00251 (2020).

6 Piana, S., Donchev, A. G., Robustelli, P. & Shaw, D. E. Water dispersion interactions strongly influence simulated structural properties of disordered protein states. *J Phys Chem B* **119**, 5113-5123, doi:10.1021/jp508971m (2015).

7 Tucker, M. R., Piana, S., Tan, D., LeVine, M. V. & Shaw, D. E. Development of Force Field Parameters for the Simulation of Single- and Double-Stranded DNA Molecules and DNA-Protein Complexes. *J Phys Chem B* **126**, 4442-4457, doi:10.1021/acs.jpcb.1c10971 (2022).

8 Wang, F. *et al.* All-Atom Simulations Reveal How Single-Point Mutations Promote Serpin Misfolding. *Biophys J* **114**, 2083-2094, doi:10.1016/j.bpj.2018.03.027 (2018).

9 Ahn, M. *et al.* Amyloid Forming Human Lysozyme Intermediates are Stabilized by Non-Native Amide-π Interactions. *Advanced Science* **n/a**, e03957, doi:https://doi.org/10.1002/advs.202503957 (2025).

10 Waudby, C. A. *et al.* Systematic mapping of free energy landscapes of a growing filamin domain during biosynthesis. *Proc Natl Acad Sci U S A* **115**, 9744-9749, doi:10.1073/pnas.1716252115 (2018).

11 Streit, J. O. *et al.* Long-range electrostatic forces govern how proteins fold on the ribosome. *bioRxiv*, 2025.2002.2010.637539, doi:10.1101/2025.02.10.637539 (2025).

12 Streit, J. O. *et al.* The ribosome lowers the entropic penalty of protein folding. *Nature* **633**, 232-239, doi:10.1038/s41586-024-07784-4 (2024).

13 Mitropoulou, A. N. *et al.* The ribosome directs nascent chains through two folding-dependent pathways. *bioRxiv*, 2025.2004.2008.647855, doi:10.1101/2025.04.08.647855 (2025).

14 Wlodarski, T. *et al.* Bayesian reweighting of biomolecular structural ensembles using heterogeneous cryo-EM maps with the cryoENsemble method. *Sci Rep* **14**, 18149, doi:10.1038/s41598-024-68468-7 (2024).

15 Burridge, C. *et al.* Nascent chain dynamics and ribosome interactions within folded ribosome-nascent chain complexes observed by NMR spectroscopy. *Chem Sci* **12**, 13120-13126, doi:10.1039/d1sc04313g (2021).

16 Lindorff-Larsen, K., Piana, S., Dror, R. O. & Shaw, D. E. How fast-folding proteins fold. *Science* **334**, 517-520, doi:10.1126/science.1208351 (2011).

17 Wright, C. F., Lindorff-Larsen, K., Randles, L. G. & Clarke, J. Parallel protein-unfolding pathways revealed and mapped. *Nat Struct Biol* **10**, 658-662, doi:10.1038/nsb947 (2003).

18 Schwaiger, I., Kardinal, A., Schleicher, M., Noegel, A. A. & Rief, M. A mechanical unfolding intermediate in an actin-crosslinking protein. *Nat Struct Mol Biol* **11**, 81-85, doi:10.1038/nsmb705 (2004).

19 Bhatia, S., Krishnamoorthy, G. & Udgaonkar, J. B. Mapping Distinct Sequences of Structure Formation Differentiating Multiple Folding Pathways of a Small Protein. *J Am Chem Soc* **143**, 1447-1457, doi:10.1021/jacs.0c11097 (2021).

20 Radford, S. E., Dobson, C. M. & Evans, P. A. The folding of hen lysozyme involves partially structured intermediates and multiple pathways. *Nature* **358**, 302-307, doi:10.1038/358302a0 (1992).

21 Zaidi, F. N., Nath, U. & Udgaonkar, J. B. Multiple intermediates and transition states during protein unfolding. *Nature Structural Biology* **4**, 1016-1024, doi:10.1038/nsb1297-1016 (1997).

22 Fowler, S. B. *et al.* Mechanical Unfolding of a Titin Ig Domain: Structure of Unfolding Intermediate Revealed by Combining AFM, Molecular Dynamics Simulations, NMR and Protein Engineering. *Journal of Molecular Biology* **322**, 841-849, doi:https://doi.org/10.1016/S0022-2836(02)00805-7 (2002).

23 Fowler, S. B. & Clarke, J. Mapping the folding pathway of an immunoglobulin domain: structural detail from Phi value analysis and movement of the transition state. *Structure* **9**, 355-366, doi:10.1016/s0969-2126(01)00596-2 (2001).

24 Schwaiger, I., Schleicher, M., Noegel, A. A. & Rief, M. The folding pathway of a fast-folding immunoglobulin domain revealed by single-molecule mechanical experiments. *Embo Rep* **6**, 46-51, doi:10.1038/sj.embor.7400317 (2005).
