# Supplementary material for: Structures of protein folding intermediates on the ribosome
Source: Nat Struct Mol Biol. 2026 Jun 16;33(6):962–72. doi: 10.1038/s41594-026-01814-7 (PMC13275320; doi:10.1038/s41594-026-01814-7)
Supplement: Supplementary file 2 — Reporting Summary [file 41594_2026_1814_MOESM2_ESM.pdf]

nature portfolio

Corresponding author(s): John Christodoulou

Last updated by author(s): Mar 25, 2026

Reporting Summary

Nature Portfolio wishes to improve the reproducibility of the work that we publish. This form provides structure for consistency and transparency in reporting. For further information on Nature Portfolio policies, see our Editorial Policies and the Editorial Policy Checklist.

Statistics

For all statistical analyses, confirm that the following items are present in the figure legend, table legend, main text, or Methods section.

n/a	Confirmed	
☐	☒	The exact sample size (n) for each experimental group/condition, given as a discrete number and unit of measurement
☐	☒	A statement on whether measurements were taken from distinct samples or whether the same sample was measured repeatedly
☒	☐	The statistical test(s) used AND whether they are one- or two-sided<br>*Only common tests should be described solely by name; describe more complex techniques in the Methods section.*
☒	☐	A description of all covariates tested
☐	☒	A description of any assumptions or corrections, such as tests of normality and adjustment for multiple comparisons
☐	☒	A full description of the statistical parameters including central tendency (e.g. means) or other basic estimates (e.g. regression coefficient) AND variation (e.g. standard deviation) or associated estimates of uncertainty (e.g. confidence intervals)
☒	☐	For null hypothesis testing, the test statistic (e.g. F, t, r) with confidence intervals, effect sizes, degrees of freedom and P value noted<br>*Give P values as exact values whenever suitable.*
☐	☒	For Bayesian analysis, information on the choice of priors and Markov chain Monte Carlo settings
☒	☐	For hierarchical and complex designs, identification of the appropriate level for tests and full reporting of outcomes
☒	☐	Estimates of effect sizes (e.g. Cohen's d, Pearson's r), indicating how they were calculated

Our web collection on statistics for biologists contains articles on many of the points above.

Software and code

Policy information about availability of computer code

Data collection	NMR data were recorded using Topspin 3.5pl2, pulse sequences available on https://github.com/shschan. MD simulations were performed and processed with GROMACS version 2021.3.<br>MD was performed (with GROMACS version 2020), PLUMED (version 2.6) and SMOG (version 2.3). For model building and visualisation, PyMol (version 2.3) and ChimeraX (version 1.9) were used.
Data analysis	NMR data were analysed using CCPN (version 2.4), nmrPipe (version 11.7), Julia (version 1.5), and MATLAB (R2017b, The MathWorks Inc.), codes are available on github.com/shschan/NMR-fit. Python analyses utilised version 3.7. Python scripts used to predict aromatic ring interactions for fluorinated protein variants are available on Github (https://github.com/julian-streit/RingCurrents19F). CryoENsemble was performed using the source code on github.com/dydymos/cryoENsemble.

For manuscripts utilizing custom algorithms or software that are central to the research but not yet described in published literature, software must be made available to editors and reviewers. We strongly encourage code deposition in a community repository (e.g. GitHub). See the Nature Portfolio guidelines for submitting code & software for further information.

Data

Policy information about availability of data

All manuscripts must include a data availability statement. This statement should provide the following information, where applicable:

- Accession codes, unique identifiers, or web links for publicly available datasets
- A description of any restrictions on data availability
- For clinical datasets or third party data, please ensure that the statement adheres to our policy

Data supporting the findings of this study are included in the article, source data, and extended data. The structural ensembles of FLN5+47 I1 and I2 and NMR data are available on Zenodo (https://doi.org/10.5281/zenodo.16601045; https://doi.org/10.5281/zenodo.19210765). This study made use of the following public datasets deposited in the PDB (https://wwww.rcsb.org): 1QFH, 1TIT, 1WLH, and 2W0P.

Research involving human participants, their data, or biological material

Policy information about studies with human participants or human data. See also policy information about sex, gender (identity/presentation), and sexual orientation and race, ethnicity and racism.

Reporting on sex and gender	N/A
Reporting on race, ethnicity, or other socially relevant groupings	N/A
Population characteristics	N/A
Recruitment	N/A
Ethics oversight	N/A

Note that full information on the approval of the study protocol must also be provided in the manuscript.

Field-specific reporting

Please select the one below that is the best fit for your research. If you are not sure, read the appropriate sections before making your selection.

☒ Life sciences ☐ Behavioural & social sciences ☐ Ecological, evolutionary & environmental sciences

For a reference copy of the document with all sections, see nature.com/documents/nr-reporting-summary-flat.pdf

Life sciences study design

All studies must disclose on these points even when the disclosure is negative.

Sample size	No samples sizes were predetermined. Samples sizes were chosen according to standards generally accepted in the protein folding/structural & computational biology fields. NMR experiments were summed from multiple experiments (generally >20) until signal/noise was sufficiently high, which is typical for NMR studies. All samples undergo rigorous biochemical and NMR quality control measurements, as described in Methods. For PRE-NMR experiments, we performed multiple (n=6) biological repeats (independent sample purifications) and reproduced the data within uncertainty of the measurements.
Data exclusions	No data were excluded.
Replication	All independent attempts to replicate the NMR data were successful. The MD ensembles were concatenated from 15 independent trajectories initiated from different initial coordinates and velocities.
Randomization	N/A, as typical for NMR and structural biology studies. Experiments and simulations were rationally designed to be systematic and answer specific technical and biological questions and therefore randomization was not applicable. All experiments and simulations were performed under well-controlled conditions.
Blinding	N/A, as typical for NMR and structural biology studies. Our data analysis was systematic without any possible prior knowledge about the result and, thus, blinding was not applicable.

Reporting for specific materials, systems and methods

We require information from authors about some types of materials, experimental systems and methods used in many studies. Here, indicate whether each material, system or method listed is relevant to your study. If you are not sure if a list item applies to your research, read the appropriate section before selecting a response.

Materials & experimental systems

n/a	Involved in the study	
☐	☒	Antibodies
☒	☐	Eukaryotic cell lines
☒	☐	Palaeontology and archaeology
☒	☐	Animals and other organisms
☒	☐	Clinical data
☒	☐	Dual use research of concern
☒	☐	Plants

Methods

n/a	Involved in the study	
☒	☐	ChIP-seq
☒	☐	Flow cytometry
☒	☐	MRI-based neuroimaging

Antibodies

Antibodies used	Anti-histidine tag (1:5000 dilution, Invitrogen MA1-21315-HRP, lot WK337821) for western blotting.
Validation	Western blot visualisation as described on the manufacturers' websites:<br>The anti-histidine antibody was verified by relative expression and cell treatment to confirm specificity to the antigen.

Plants

Seed stocks	N/A
Novel plant genotypes	N/A
Authentication	N/A
