# Supplementary material for: Structures of protein folding intermediates on the ribosome
Source: Nat Struct Mol Biol. 2026 Jun 16;33(6):962–72. doi: 10.1038/s41594-026-01814-7 (PMC13275320; doi:10.1038/s41594-026-01814-7)
Supplement: Supplementary file 3 — Peer Review file [file 41594_2026_1814_MOESM3_ESM.pdf]

nature portfolio

Peer Review File

Structures of protein folding intermediates on the ribosome

Corresponding Author: Professor John Christodoulou

Version 0:

Decision Letter:

11th Dec 2025

Dear Professor Christodoulou,

Thank you again for submitting your manuscript "Structures of protein folding intermediates on the ribosome" to the journal. I am writing on behalf of the handling editor of your manuscript, Dr. Osman who is currently sick, out of office and did not intend for our decision to be delayed, therefore sending the editorial decision was delegated to me.

As indicated in our original communication, we recruited new relevant reviewers in Molecular Dynamics Simulations, who are also experts in translation and ribosomal biology. These co-reviewing Reviewers are numbered as Reviewer #4 and #5 and assessed the manuscript together. We asked them to comment on the previous remarks of Reviewer #2 as well as your responses to these concerns.

You will see that these two co-reviewing experts are in principle positive but retain a few concerns that require convincing answers and changes in a final minor revision. In particular, they raise relevant questions about ED9c and how it fits with other data, about the purpose of the kinetic model in ED1, and about the approximation of direct rates. They also recommend adding missing relevant explanations in different Method subsections and comparative analyses between this dataset and a previously published one. Finally, we ask that you please address the reviewers' remaining concerns in full, including textually addressing all minor points raised and streamlining the manuscript.

Please also be sure to respond to all concerns of the referees in full in a designated, stand alone point-by-point response and highlight all changes in the revised manuscript text file. We expect to see your revised manuscript within 1-2 months. We are committed to providing a fair and constructive peer-review process; please do not hesitate to contact us if you would like to discuss the reviews or anticipate any delay with the resubmission. If you cannot send it within this time, please contact us to discuss an extension; we will still consider your revision provided that no similar work has been accepted for publication at NSMB or published elsewhere.

REPORTING AND DATA AVAILABILITY: Please provide updated files as follows with your revision. As you already know, we put great emphasis on ensuring that the methods and statistics reported in our papers are correct and accurate.

1. REPORTING SUMMARY: Please provide an updated file:

https://www.nature.com/documents/nr-reporting-summary.pdf

This form is a dynamic ‘smart pdf’ and must be downloaded and completed in Adobe Reader.

2. REPORTING OF STRUCTURAL DATA: If there are additional or modified structures in the revision, please submit the corresponding PDB validation reports with your other files, and use the figshare integration system to provide access to maps, half-maps, and models.

Manuscripts reporting new structures should contain a table summarizing structural and refinement statistics. Please include these tables for new cryo-EM or -ET (https://www.nature.com/documents/nr-tables-cryo-em.doc and modifying this file for ET), NMR (https://www.nature.com/documents/nr-tables-nmr.doc) and X-ray crystallography (https://www.nature.com/documents/nr-tables-xray.doc). To facilitate assessment of the quality of the structural data, a stereo image of a portion of the electron density map (for crystallography papers) or of the superimposed lowest energy structures (>10; for NMR papers) should be provided with the submitted manuscript. If the reported structure represents a novel overall

fold, a stereo image of the entire structure (as a backbone trace) should also be provided. For cryo-EM structures, a representative micrograph showing individual particles should be provided in the figures alongside a processing workflow (please see our Editorial for more information: https://www.nature.com/articles/s41594-025-01567-9).

DATA DEPOSITION: We require deposition of coordinates (and, in the case of crystal structures, structure factors) into the Protein Data Bank with the designation of immediate release upon publication (HPUB). Electron microscopy-derived density maps and coordinate data must be deposited in EMDB and released upon publication. Deposition and immediate release of NMR chemical shift assignments are requested. To avoid delays in publication, dataset accession numbers must be supplied with the revised manuscript and appropriate release dates must be indicated at the galley proof stage.

3. REPORTING OF LIGHT MICROSCOPY DATA: For any revision that includes light microscopy data, we ask our authors to please include a completed light microscopy reporting table [https://www.nature.com/documents/Light_microscopy_reporting_table.xlsx] to ensure the methods are described thoroughly. The table will be available to reviewers and

ultimately published, should the manuscript be accepted at the journal. When submitting the revised version of your manuscript, please pay close attention to our href="https://www.nature.com/nature-portfolio/editorial-policies/image-integrity">Digital Image Integrity Guidelines.

4. CODE AND COMPUTATIONAL WORK: If newly developed, unpublished code was used in this work, it must be provided with the revision and access to it must be disclosed in the Code Availability Statement in the manuscript. Please also provide the completed software submission checklist. This form is a dynamic 'smart pdf' and must be downloaded and completed in Adobe Reader, instead of opening it in a web browser: https://www.nature.com/documents/nr-software-policy.pdf

If molecular dynamics (MD) simulations were performed, please refer to this Editorial and provide a completed MD simulations checklist with your revision: https://www.nature.com/articles/s42003-023-04653-0

Lastly, if it applies, please complete and upload the completed machine learning checklist with your submission for re-review: https://www.nature.com/documents/machine-learning-checklist.pdf

5. SOURCE GEL IMAGES: Unprocessed scans should be provided for all gels and western blots presented in figures and should be clearly labelled with the figure number in the file title. The source gel images should be uncropped, unmodified images, with molecular weight markers. Uncropped, unmodified gel images from existing experimental repeats, in addition to the data shown in the figures, can also be presented in source data. Please provide these files as PDF with your revision.

Please ensure that all control panels for gels and western blots are appropriately described as loading or IP controls. Lastly, please ensure that all images in the paper are checked for duplication of panels and for splicing of gel lanes.

6. SOURCE NUMERICAL DATA: we urge authors to provide, in tabular form in excel files, the data underlying the graphical representations used in figures. This is to further increase transparency in data reporting, as detailed in this editorial (http://www.nature.com/nsmb/journal/v22/n10/full/nsmb.3110.html). Please provide one excel file per figure max, with one panel per tab. When submitting files, the title field should indicate which figure the source data pertains to.

7. DATA AVAILABILITY: this journal strongly supports public availability of data. All data used in accepted papers should be available via a public data repository, or alternatively, as Supplementary Information. If data can only be shared on request, please explain why in your Data Availability Statement and in the correspondence with your editor.

For some data types, deposition in a public repository is mandatory as detailed below:

https://www.nature.com/nature-research/editorial-policies/reporting-standards#availability-of-data

Finally, please ensure that you retain unprocessed data and metadata files after publication, ideally archiving data in perpetuity, as these may be requested during the peer review and production process or after publication if any issues arise.

EXTENDED DATA FIGURES

When re-submitting your manuscript, please ensure that any supplementary figures and tables that are crucial to the manuscript's conclusions are converted into Extended Data figures and tables to increase visibility of these data. Extended Data figures and tables are online-only (present in the online PDF and full-text HTML versions of the paper), peer-reviewed display items that provide essential background to the article but are not included in the main article due to space constraints. A maximum of ten Extended Data display items (figures and tables) is permitted.

While we encourage the use of color in preparing figures, please note that this will incur a charge to partially defray the cost of printing. Information about color charges can be found at http://www.nature.com/nsmb/authors/submit/index.html#costs

Nature Structural & Molecular Biology is committed to improving transparency in authorship. As part of our efforts in this direction, we are now requesting that all authors identified as 'corresponding author' on published papers create and link their Open Researcher and Contributor Identifier (ORCID) with their account on the Manuscript Tracking System (MTS), prior to acceptance. This applies to primary research papers only. ORCID helps the scientific community achieve unambiguous attribution of all scholarly contributions. You can create and link your ORCID from the home page of the MTS by clicking on 'Modify my Springer Nature account'. For more information please visit please visit www.springernature.com/orcid.

Please use the link below to submit your revised manuscript and related files:

Link Redacted

Note: This URL links to your confidential home page and associated information about manuscripts you may have submitted, or that you are reviewing for us. If you wish to forward this email to co-authors, please delete the link to your homepage.

We look forward to seeing the revised manuscript and thank you for the opportunity to review your work.

Sincerely,

Dimitris Typas
Senior Editor
Nature Structural & Molecular Biology
ORCID: 0000-0002-8737-1319

on behalf of

Sara Osman, Ph.D.
Senior Editor, Nature Structural & Molecular Biology
Nature Portfolio

Reviewers' Comments:

Reviewer #4 (Remarks to the Author):

In their manuscript "Structures of protein folding intermediates on the ribosome", Chan et al. characterized the structures of protein folding intermediates during co-translational folding (coTF) on the ribosome. By integrating 19F NMR spectroscopy with molecular dynamics (MD) simulations, and biochemical methods, they provide atomistic structural ensembles of two distinct coTF intermediates (I1 and I2) of FLN5 that are in quantitative agreement with the available experimental data.

This report is carried out with the involvement of an early career researcher as encouraged by the Editor. For that reason, we write in the first person plural.

The I1 and I2 partially folded intermediates were already identified by the authors in a previous publication (Chan et al., Nature Chemistry, 2022). Also in their 2022 paper the 19F NMR findings were complemented with MD simulations, both coarse grained (CG) and all-atom. Specifically, they used CG and density-fitting MD simulations with structure-based models.
The CG models provided structures that were only qualitatively in agreement with the 19F NMR data of I2. From the density-fitting MD simulations, they observed that the cryo-EM data corroborate the proposed intermediate structures.

In this manuscript, atomistic simulations with the DES-Amber force field were used instead of CG structure-based models. This allowed the authors to propose atomistic models of I1 and I2 corroborated by their experimental data. To sample coTF intermediates, due to the long timescales of the process, they first used biased simulations (ratchet-and-pawl MD) from which they observed structures that they attributed to intermediate I1. These structures were used as starting conformations for unbiased MD simulations. The starting conformations for the MD simulations of the I2 state were instead built based on previous NMR data.

The authors assessed the validity of the MD structural ensembles against data from 19F NMR spectra, cross-linking experiments and cryo-EM maps. The comparisons were quantitative and systematic, providing a solid foundation to address a central question of the study: How can the I1 and I2 co-translational folding intermediates be structurally defined at atomistic resolution?

Although the time scales accessed by MD do not allow a comprehensive sampling of the full coTF landscape, the two intermediates reported here are well characterized. This atomistic characterization, validated by extensive comparison to experimental data, represents the principal novelty of this study and marks the main advance over previous coarse-grained models.

In addition, the authors proposed that:

I1 and I2 are populated along two parallel folding pathways. As the data reported in this manuscript cannot fully support this statement, the addition of lines 564-566 and of Supplementary Note 6 is a necessary clarification.

Pro742 in FLN5 acts as a molecular switch of the G-strand folding, biasing the folding flux between I1 (cis-Pro742, native G-strand fold) and I2 (trans-Pro742, disordered G-strand). They support this observation by WT-METAD simulations, which suggest that the cis conformation is energetically preferred in the native G-strand fold.
This regulatory role seems to be, however, not entirely novel, as the 2022 study had already demonstrated that substituting Pro742 with alanine, which disfavors the cis conformation, led to destabilization of I1. What would strengthen the mechanistic insight is a direct comparison of $\Delta\Delta G$ values between the simulation-derived energetic landscapes presented here and the $\Delta\Delta G$ values reported in the 2022 paper.

Overall, we consider this manuscript suitable for publication in Nature Structural & Molecular Biology. However, we would like to raise a few additional concerns that should be addressed prior to publication.

Regarding the comparison with the cryo-EM maps: if in AP1 and P2 states the I1 state has a lower population than I2 (Extended Figure 9b), why is the posterior of I1 higher than the one of I2 in Extended Figure 9c?

The purpose of the kinetic model in Extended Figure 1 is not clear, especially considering that none of the models seem to reproduce the experiential data. In addition, the approximation of all direct rates to kf and the linear interpolation of the length-dependent kf seems arbitrary and not sufficiently justified.

Overall, the manuscript could benefit from a tighter focus on the main results, while some of the secondary results could be trimmed or better contextualized.
Additionally, we recommend that the authors add a brief explanation to each subsection in the Methods of the question(s) specifically addressed with this method. This is currently done inconsistently and, given the large number of different methods, would help clarify and make it easier for the reader to understand how each technique supports the main findings.

Minor corrections:

Figure 3, line 331 : “b, Lowest energy …” should be replaced with “d, Lowest energy …”
Extended Figure 5: in the description l and m should be inverted
Supplementary Note2, line 1075: the phrasing “local minimum within the kinetic energy landscape” has not been replaced yet.

Reviewer #5 (Remarks to the Author):

I co-reviewed this manuscript with one of the reviewers who provided the listed reports. This is part of the Nature Structural & Molecular Biology initiative to facilitate training in peer review and to provide appropriate recognition for Early Career Researchers who co-review manuscripts.

Version 1:

Decision Letter:

Our ref: NSMB-A51971A

29th Jan 2026

Dear Dr. Christodoulou,

Thank you for submitting your revised manuscript "Structures of protein folding intermediates on the ribosome" (NSMB-A51971A). It has now been seen by the original referee and their comments are below. The reviewers find that the paper has improved in revision, and therefore we'll be happy in principle to publish it in Nature Structural & Molecular Biology, pending minor revisions to satisfy the referees' final requests and to comply with our editorial and formatting guidelines.

We are now performing detailed checks on your paper and will send you a checklist detailing our editorial and formatting requirements in the next few weeks. Please do not upload the final materials and make any revisions until you receive this additional information from us.

To facilitate our work at this stage, it is important that we have a copy of the main text as a word file. If you could please send

along a word version of this file as soon as possible, we would greatly appreciate it; please make sure to copy the NSMB account (cc'ed above).

Thank you again for your interest in Nature Structural & Molecular Biology Please do not hesitate to contact me if you have any questions.

Sincerely,
Sara

Sara Osman, Ph.D.
Senior Editor
Nature Structural & Molecular Biology

Reviewer #4 (Remarks to the Author):

All of my concerns have been sufficiently addressed and, from my side the manuscript, can be accepted for publication.

Reviewer #5 (Remarks to the Author):

I co-reviewed this manuscript with one of the reviewers who provided the listed reports. This is part of the Nature Structural & Molecular Biology initiative to facilitate training in peer review and to provide appropriate recognition for Early Career Researchers who co-review manuscripts.

Open Access This Peer Review File is licensed under a Creative Commons Attribution 4.0 International License, which permits use, sharing, adaptation, distribution and reproduction in any medium or format, as long as you give appropriate credit to the original author(s) and the source, provide a link to the Creative Commons license, and indicate if changes were made.
In cases where reviewers are anonymous, credit should be given to 'Anonymous Referee' and the source.
The images or other third party material in this Peer Review File are included in the article’s Creative Commons license, unless indicated otherwise in a credit line to the material. If material is not included in the article’s Creative Commons license and your intended use is not permitted by statutory regulation or exceeds the permitted use, you will need to obtain permission directly from the copyright holder.
To view a copy of this license, visit https://creativecommons.org/licenses/by/4.0/

Response to reviewers
Structures of protein folding intermediates on the ribosome, Chan et al.

We are grateful to both reviewers for their time and effort to provide us with their valuable and carefully considered feedback to improve our manuscript. We attach a revised version of the manuscript (changes in blue), and our point-by-point response to the reviewers' comments (also in blue) is detailed below.

Reviewer #4 (Remarks to the Author):

In their manuscript "Structures of protein folding intermediates on the ribosome", Chan et al. characterized the structures of protein folding intermediates during co-translational folding (coTF) on the ribosome. By integrating 19F NMR spectroscopy with molecular dynamics (MD) simulations, and biochemical methods, they provide atomistic structural ensembles of two distinct coTF intermediates (I1 and I2) of FLN5 that are in quantitative agreement with the available experimental data.

This report is carried out with the involvement of an early career researcher as encouraged by the Editor. For that reason, we write in the first-person plural.

The I1 and I2 partially folded intermediates were already identified by the authors in a previous publication (Chan et al., Nature Chemistry, 2022). Also in their 2022 paper the 19F NMR findings were complemented with MD simulations, both coarse grained (CG) and all-atom. Specifically, they used CG and density-fitting MD simulations with structure-based models. The CG models provided structures that were only qualitatively in agreement with the 19F NMR data of I2. From the density-fitting MD simulations, they observed that the cryo-EM data corroborate the proposed intermediate structures.

In this manuscript, atomistic simulations with the DES-Amber force field were used instead of CG structure-based models. This allowed the authors to propose atomistic models of I1 and I2 corroborated by their experimental data. To sample coTF intermediates, due to the long timescales of the process, they first used biased simulations (ratchet-and-pawl MD) from which they observed structures that they attributed to intermediate I1. These structures were used as starting conformations for unbiased MD simulations. The starting conformations for the MD simulations of the I2 state were instead built based on previous NMR data.

The authors assessed the validity of the MD structural ensembles against data from 19F NMR spectra, cross-linking experiments and cryo-EM maps. The comparisons were quantitative and systematic, providing a solid foundation to address a central question of the study: How can the I1 and I2 co-translational folding intermediates be structurally defined at atomistic resolution?

Although the time scales accessed by MD do not allow a comprehensive sampling of the full coTF landscape, the two intermediates reported here are well characterized. This atomistic characterization, validated by extensive comparison to experimental data, represents the principal novelty of this study and marks the main advance over previous coarse-grained models.

We thank the reviewers for recognising the main advance present in our study, namely the extensive characterisation of folding intermediates on the ribosome for the first time at atomistic resolution and quantitatively validated by extensive experimental data, including inter-residue contacts extracted by an entirely novel approach by ^{19}F NMR.

In addition, the authors proposed that:

I1 and I2 are populated along two parallel folding pathways. As the data reported in this manuscript cannot fully support this statement, the addition of lines 564-566 and of Supplementary Note 6 is a necessary clarification.

Thank you for confirming this view - we completely agree.

Pro742 in FLN5 acts as a molecular switch of the G-strand folding, biasing the folding flux between I1 (cis-Pro742, native G-strand fold) and I2 (trans-Pro742, disordered G-strand). They support this observation by WT-METAD simulations, which suggest that the cis conformation is energetically preferred in the native G-strand fold. This regulatory role seems to be, however, not entirely novel, as the 2022 study had already demonstrated that substituting Pro742 with alanine, which disfavors the cis conformation, led to destabilization of I1. What would strengthen the mechanistic insight is a direct comparison of ΔΔG values between the simulation-derived energetic landscapes presented here and the ΔΔG values reported in the 2022 paper.

We thank the reviewer for this comment and indeed, we have previously reported the regulatory role of Pro742. As suggested, we have therefore calculated a value for $\Delta\Delta G_{N\text{-}U,cis\text{-}trans}$ (i.e., difference in folding free energy between the cis and trans state). Within a thermodynamic cycle detailed in panel n in the revised Extended Data Figure 5, we now show that with the computed (via WT-METAD) $\Delta G_{cis\text{-}trans}$ values for the unfolded peptide and native state we obtain a $\Delta\Delta G_{N\text{-}U,cis\text{-}trans}$ of 3.70 ± 0.38 kcal mol^{-1}, which is quantitatively consistent with the experimental value for the $\Delta\Delta G_{N\text{-}U}$ comparing wild-type with P742A (4.00 ± 0.10 kcal mol^{-1}). This reinforces the mechanistic insight of the regulatory role of P742, and the strong contribution of P742 cis-trans isomerisation to both the local and global stability of the protein. We have now added a note to this effect in Supplementary Note 2.

Overall, we consider this manuscript suitable for publication in Nature Structural & Molecular Biology. However, we would like to raise a few additional concerns that should be addressed prior to publication.

Thank you for the overall positive feedback.

Regarding the comparison with the cryo-EM maps: if in AP1 and P2 states the I1 state has a lower population than I2 (Extended Figure 9b), why is the posterior of I1 higher than the one of I2 in Extended Figure 9c?

The reviewers are correct that, after reweighting with the AP1 and P2 cryo-EM maps, the population of I1 is lower than that of I2 (Extended Data Figure 9b). However, Extended Data Fig. 9c does not report state populations; instead, it shows the cross-correlation value between each individual state (I1, I2, or N) and the cryo-EM maps, evaluated before and after reweighting.

This analysis was performed to illustrate that the overall agreement between the experimental maps and the simulated ensembles improves when all three states are included during reweighting. Importantly, a higher cross-correlation for a single state does not imply that this state will be the most populated in the reweighted ensemble. In ensemble-based reweighting, a conformation that best matches the experimental data in isolation may still carry a low statistical weight when multiple states are considered simultaneously. This typically reflects the fact that the experimental data are consistent with an average over several conformations rather than a single dominant state. For example, an ensemble average of two states (here N and I2), each of which individually agrees only moderately with the map, can yield a better overall fit than either state alone. We have added a note to the figure legend of Extended Data Fig. 9c to clarify this distinction.

The purpose of the kinetic model in Extended Figure 1 is not clear, especially considering that none of the models seem to reproduce the experiential data. In addition, the approximation of all direct rates to kf and the linear interpolation of the length-dependent kf seems arbitrary and not sufficiently justified.

We thank the reviewers for the opportunity to clarify the purpose and scope of the kinetic model shown in Extended Data Figure 1. The aim of this model is not to reproduce the experimental equilibrium populations measured by ^{19}F NMR, but rather to assess whether the folding intermediates I1 and I2 are expected to be populated on the ribosome during *real-time translation*, a regime that is not accessible experimentally by ^{19}F NMR. Indeed, we used the experimental

equilibrium populations to build the real-time translation model. We have clarified this motivation explicitly in the main text (revised lines 96-99). Consequently, we do not expect quantitative agreement between the modelled populations, which are obtained under non-equilibrium conditions with a finite translation rate, and the experimental data, which correspond to equilibrium conditions (i.e., a translation rate of zero).

We have also clarified the assumptions underlying the kinetic model in the Methods section (from line 704). Specifically, we use the experimentally measured apparent folding rate of the isolated protein, k_f (Waudby et al., ref. 11), as a conservative upper bound (i.e. fastest) for folding transitions since our CEST NMR measurements show that folding is decelerated on (compared to off) the ribosome (Extended Data Figure 1b-c). Any additional intermediate state-related transitions that are not directly resolved experimentally would necessarily occur on faster timescales in order to yield the observed apparent folding rate. Moreover, previous experimental work has shown that folding rates increase more rapidly than predicted by linear interpolation as a function of linker length (Liu et al., ref. 34). Our use of linear interpolation therefore represents a deliberately conservative approximation that underestimates folding rates. As such, we do not expect that adopting more realistic, non-linear length dependencies would alter the qualitative conclusions of the model.

Finally, varying k_f has an effect equivalent to varying the translation rate, since the competition between these rates determines the extent to which intermediates are populated during translation. As shown in Extended Data Fig. 1a, even under conditions corresponding to a fourfold increase in translation speed (or equivalently a fourfold decrease in k_f), the intermediates I1 and I2 remain kinetically accessible during real-time translation. Taken together, this analysis supports the conclusion that FLN5 folding intermediates observed at equilibrium are expected to form during real-time translation across a broad and conservative range of kinetic conditions.

Overall, the manuscript could benefit from a tighter focus on the main results, while some of the secondary results could be trimmed or better contextualized.

We appreciate this opportunity and have now streamlined the text throughout, including by:

- focusing on our main result in the revised, shortened Abstract,
- reducing the length of our discussion of the TF experiments (and folding pathways) in the main text, lines 438-451, and
- de-emphasising our secondary conclusions (including parallel folding pathways) by rewording and reducing the length of our Discussion (for example, lines 558-573, 605-607).

Additionally, we recommend that the authors add a brief explanation to each subsection in the Methods of the question(s) specifically addressed with this method. This is currently done inconsistently and, given the large number of different methods, would help clarify and make it easier for the reader to understand how each technique supports the main findings.

We thank the reviewers for bringing this to our attention and have added a brief explanation for all techniques now (see for example, lines 650-651, 666, 695, etc.).

Minor corrections:

Figure 3, line 331 : “b, Lowest energy …” should be replaced with “d, Lowest energy …”
Extended Figure 5: in the description l and m should be inverted
Supplementary Note2, line 1075: the phrasing “local minimum within the kinetic energy landscape” has not been replaced yet.

Thank you for pointing out these minor errors, we have now corrected them.

Version 2:

Decision Letter:

27th Apr 2026

Dear Dr. Christodoulou,

We are now happy to accept your revised paper "Structures of protein folding intermediates on the ribosome" for publication as an Article in Nature Structural & Molecular Biology.

Acceptance is conditional on the manuscript's not being published elsewhere and on there being no announcement of this work to the newspapers, magazines, radio or television until the publication date in Nature Structural & Molecular Biology.

Over the next few weeks, your paper will be copyedited to ensure that it conforms to Nature Structural & Molecular Biology style. Once your paper is typeset, you will receive an email with a link to choose the appropriate publishing options for your paper and our Author Services team will be in touch regarding any additional information that may be required.

After the grant of rights is completed, you will receive a link to your electronic proof via email with a request to make any corrections within 48 hours. If, when you receive your proof, you cannot meet this deadline, please inform us at rjsproduction@springernature.com immediately.

You will not receive your proofs until the publishing agreement has been received through our system.

Due to the importance of these deadlines, we ask that you please let us know now whether you will be difficult to contact over the next month. If this is the case, we ask you provide us with the contact information (email, phone and fax) of someone who will be able to check the proofs on your behalf, and who will be available to address any last-minute problems.

To assist our authors in disseminating their research to the broader community, our SharedIt initiative provides all co-authors with the ability to generate a unique shareable link that will allow anyone (with or without a subscription) to read the published article. Recipients of the link with a subscription will also be able to download and print the PDF.

As soon as your article is published, you can generate your shareable link by entering the DOI of your article here: http://authors.springernature.com/share. Corresponding authors will also receive an automated email with the shareable link

Note the policy of the journal on data deposition: http://www.nature.com/authors/policies/availability.html.

Your paper will be published online soon after we receive proof corrections and will appear in print in the next available issue. You can find out your date of online publication by contacting the production team shortly after sending your proof corrections.

You may wish to make your media relations office aware of your accepted publication, in case they consider it appropriate to organize some internal or external publicity. Once your paper has been scheduled you will receive an email confirming the publication details. This is normally 3-4 working days in advance of publication. If you need additional notice of the date and time of publication, please let the production team know when you receive the proof of your article to ensure there is sufficient time to coordinate. Further information on our embargo policies can be found here: https://www.nature.com/authors/policies/embargo.html

You can now use a single sign-on for all your accounts, view the status of all your manuscript submissions and reviews, access usage statistics for your published articles and download a record of your refereeing activity for the Nature journals.

If you have not already done so, we strongly recommend that you upload the step-by-step protocols used in this manuscript

to the Protocol Exchange. Protocol Exchange is an open online resource that allows researchers to share their detailed experimental know-how. All uploaded protocols are made freely available, assigned DOIs for ease of citation and fully searchable through nature.com. Protocols can be linked to any publications in which they are used and will be linked to from your article. You can also establish a dedicated page to collect all your lab Protocols. By uploading your Protocols to Protocol Exchange, you are enabling researchers to more readily reproduce or adapt the methodology you use, as well as increasing the visibility of your protocols and papers. Upload your Protocols at www.nature.com/protocolexchange/. Further information can be found at www.nature.com/protocolexchange/about.

An online order form for reprints of your paper is available at https://www.nature.com/reprints/author-reprints.html. Please let your coauthors and your institutions' public affairs office know that they are also welcome to order reprints by this method.

Authors may need to take specific actions to achieve compliance with funder and institutional open access mandates. If your research is supported by a funder that requires immediate open access (e.g. according to Plan S principles or the NIH public access policy) then you should select the gold OA route, and we will direct you to the compliant route where possible. Because authors warrant under our subscription licensing terms that they haven’t committed to licensing any version of their article under a licence inconsistent with the terms of our agreement – including the applicable embargo period – publication under the subscription model isn’t suitable for authors whose funders require no embargo.

In approximately 10 business days you will receive an email with a link to choose the appropriate publishing options for your paper and our Author Services team will be in touch regarding any additional information that may be required.

You will not receive your proofs until the publishing agreement has been received through our system.

If you have any questions about our publishing options, costs, Open Access requirements, or our legal forms, please contact ASJournals@springernature.com

Sincerely,
Sara

Sara Osman, Ph.D.
Senior Editor
Nature Structural & Molecular Biology

Click here if you would like to recommend Nature Structural & Molecular Biology to your librarian:
http://www.nature.com/subscriptions/recommend.html#forms

Open Access This Peer Review File is licensed under a Creative Commons Attribution 4.0 International License, which

permits use, sharing, adaptation, distribution and reproduction in any medium or format, as long as you give appropriate credit to the original author(s) and the source, provide a link to the Creative Commons license, and indicate if changes were made.
In cases where reviewers are anonymous, credit should be given to 'Anonymous Referee' and the source.
The images or other third party material in this Peer Review File are included in the article’s Creative Commons license, unless indicated otherwise in a credit line to the material. If material is not included in the article’s Creative Commons license and your intended use is not permitted by statutory regulation or exceeds the permitted use, you will need to obtain permission directly from the copyright holder.
To view a copy of this license, visit https://creativecommons.org/licenses/by/4.0/
